# Systematic review and consensus conceptual model of meaningful symptoms and functional impacts in early Parkinson’s Disease

**DOI:** 10.1038/s41531-025-00907-2

**Published:** 2025-04-03

**Authors:** Jennifer R. Mammen, Jamie L. Adams, Rikki Mangrum, Yuge Xiao, William Barbosa, Mirinda Tyo, Christopher Redmond, Cheryl Carter, Kelly Cifelli, Robert Cifelli, Hope Maruzo, Jim Meeker, Gerry Shultz, Colbren Thomas, Claire Bale, Evan Davies, Catherine M. Kopil, Connie Marras, Tiago Mestre, Thomas Morel, Tanya Simuni, Glenn T. Stebbins, Daniel Weintraub, Diane Stephenson

**Affiliations:** 1 University of Massachusetts, Dartmouth, College of Nursing and Health Sciences, Dartmouth, MA USA; 2 Center for Health + Technology, University of Rochester Medical Center, Rochester, NY USA; 3 Department of Neurology, University of Rochester Medical Center, Rochester, NY USA; 4 Vector Psychometric Group, LLC, Chapel Hill, NC USA; 5 The Michael J Fox Foundation for Parkinson’s Research, New York City, NY USA; 6 People affected by Parkinson’s – patient and family advisory panel, New York City, NY USA; 7 Parkinson’s UK, London, UK; 8 Roche, Basel, Switzerland; 9 University of Toronto, Toronto, Canada; 10 Parkinson’s disease and Movement Disorders Center, Division of Neurology, Department of Medicine, The Ottawa Hospital Research Institute, The University of Ottawa Brain and Research Institute, Ottawa, ON Canada; 11 UCB, Department of Patient Centred Outcomes Research, Bulle, Switzerland; 12 Northwestern University Feinberg School of Medicine, Chicago, IL USA; 13 Department of Neurological Sciences, Rush University Medical Center, Chicago, IL USA; 14 University of Pennsylvania, School of Medicine, Department of Psychiatry, Philadelphia, PA USA; 15 Critical Path Institute, Tucson, AZ USA

**Keywords:** Neurological manifestations, Research data, Parkinson's disease

## Abstract

A comprehensive, patient-centered conceptual model of early Parkinson’s is lacking and is greatly needed. A systematic review and meta-synthesis of qualitative and quantitative research was conducted by a multi-stakeholder taskforce using JBI Mixed Methods Review criteria and GRADE-CERQual standards for assessment of evidence. Over 340 symptoms and impacts were identified across ten symptom domains (Movement, Cognitive, Psychiatric, Sleep, Sensory, Speech, Digestive, Urinary, Sexual, Autonomic) and two impact domains (Physical and Psychosocial functioning). A wide range of motor and non-motor symptoms were present in early disease, with strongest support for tremor, dexterity, gait, stiffness, slow movements, cognitive, mood, and sleep alterations, urinary dysfunction, constipation, pain, and fatigue. These affected mobility, self-concept, coping, effort of living, interactions and important activities, with evidence of many understudied concepts. This model offers the most comprehensive catalogue of symptoms and impacts in Parkinson’s to date and will support clinical practice and endpoint selection for therapeutic trials.

## Introduction

Effective treatments to halt or delay progression of Parkinson’s disease (PD) are urgently needed by patients and families^1^. However, development of new drugs is a time and resource intensive process accompanied by more failures than successes^2^. This is especially true for diseases with wide heterogeneity in symptom expression and unclear biological mechanisms of progression, such as PD^3^. Phenotypic variability makes selection of pertinent outcomes for trials particularly challenging, as different symptoms or functional impacts may be more (or less) important to different people at different points throughout their disease course^4,5^. Yet, the success of clinical trials is dependent on having clinical outcome assessments (COA) that are sensitive to treatment effects rather than natural variations in disease progression or situational context^6,7^. This has created a critical need-to-know, with reasonable certainty, what experiences are typically most important to the majority of people with PD at specified stages of disease (i.e., what—who—when). This summative, contextually-defined knowledge of individuals’ lived experiences is essential to development of outcome measures that are meaningful from a real-world perspective and in alignment with the regulatory landscape^8,9^. Recent qualitative work has greatly enhanced understanding of the lived experiences of people with Parkinson’s^4,10–13^; however there is no comprehensive nor widely accepted patient-centric conceptual model that can be used to guide the field. For this reason, following the 2022 PD Endpoints Roundtable^14^, a global taskforce of experts and patient representatives was convened to develop a consensus-based conceptual model of meaningful symptoms and functional impacts for early PD from systematic review of the literature. The taskforce goals were to create a comprehensive yet parsimonious model that (1) aligns with current Food Drug Administration (FDA) guidance for patient-focused drug development (PFDD)^6–9^, (2) can support future research, practice, and clinical trials, (3) and is adaptable to emerging knowledge and later-stages of disease. This paper reports methodological approaches and findings of the taskforce.

## Results

### Sample characteristics

A total of 88 studies in early PD were utilized for the concept identification phase^4,10–13,15–97^. Of these, 56 sources were in PD < 3 years since diagnosis^4,10–13,15,16,18,19,21,22,27,29–36,38,39,43,45,46,48–50,52,54,55,57,59,60,62,66–70,73,76–79,82–88,92,95–97^. After pooling for same sample studies within this latter subset, a total of 38 unique samples were identified and used to derive frequencies for the final model. All qualitative studies (Tier 1; 6 unique samples from 7 studies) were from the UK, USA, and Canada with predominantly white participants (93–100%)^4,10–13,15,16^. Three reported bothersomeness; four reported prevalence; one reported both. Sample sizes in qualitative studies ranged from 20 to 134 with one very large sample study of 8536 participants (Fox Insight/PD PROP)^15^. Tier 2 (*N* = 13) and Tier 3 (*N* = 19) quantitative sources included studies from UK, USA, Canada, Italy, Korea, Serbia, Thailand, Germany, India, China, Singapore, and the Netherlands. Distribution of race/ethnicity was generally not reported. Samples sizes for quantitative studies ranged from 54 to 921 participants. The mean age range for all studies in all Tiers was 57–68 years. Gender distribution ranged from 40–74% male, most commonly around 60%. In 13 of 38 unique samples (34%), participants were taking PD medications (range 4–100%; mean LEDD 50–544 mg/day). However, medication use was not specified in 18% (7/38). Hoehn & Yahr score (H&Y) was reported by 27/38 samples, with mean H&Y < 2 for all, but only 12/27 (44%) with H&Y ≤2 when factoring +2 SD. MDS-UPDRS III (motor) was reported in 25/38 studies and ranged from 9.2–27.0, which is consistent with early PD^98^. Detailed characteristics for each study included in the review are presented in the Supplementary Data.

### Concept characteristics

Approximately 340 symptoms and impacts were identified from 88 publications, as shown in Tables 1–12. Substantial variability was observed in terminology and classification of concepts, with certain concepts inconsistently classified as motor vs. non-motor, (e.g., restless leg, constipation, drooling, voice changes, swallowing), impact vs. symptom (e.g., anxiety, depression, frustration), or listed twice under both symptom and impact (e.g., handwriting, anxiety). Diverse terminology was commonly used to describe conceptually similar ideas (e.g., depressed mood, feelings of sadness, negative feelings and emotions). Definitions were rarely provided for terms, requiring reviewers to infer what a concept likely comprised from common language use or from the context in the report (e.g., thermoregulation indicative of heat/cold intolerance vs. body temperature).

**Table 1:**
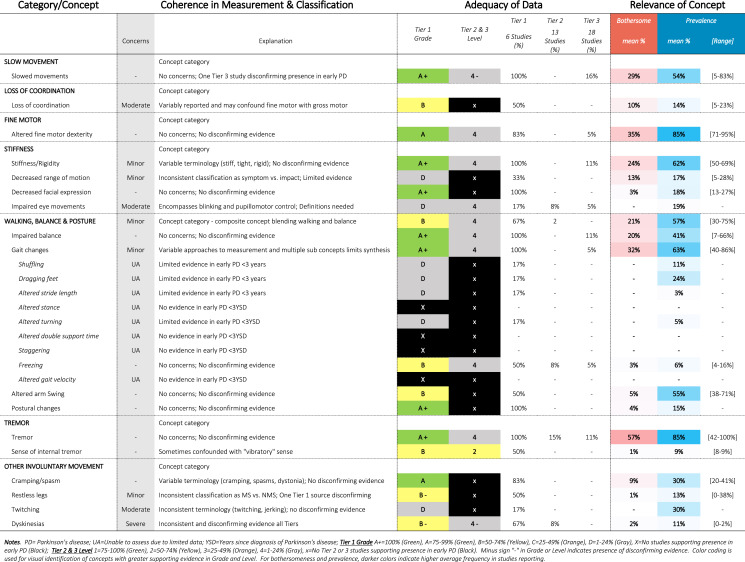
SOFT Report **-** Movement Domain

**Table 2:**
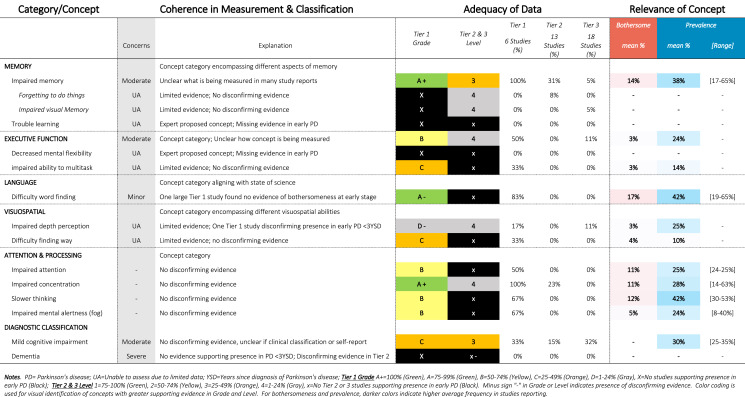
SOFT Report - Cognitive Domain

**Table 3:**
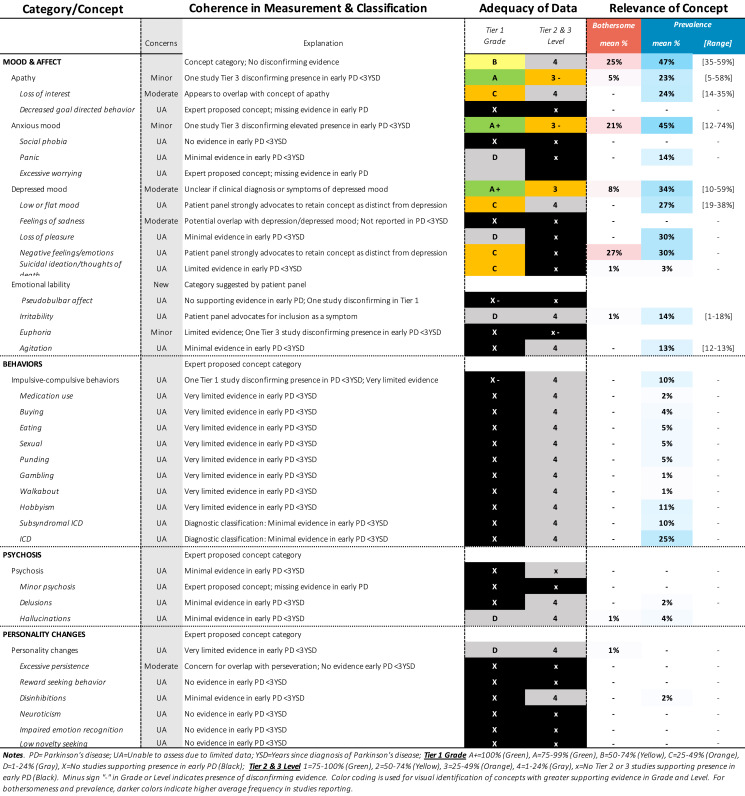
SOFT Report - Psychiatric Domain

**Table 4:**
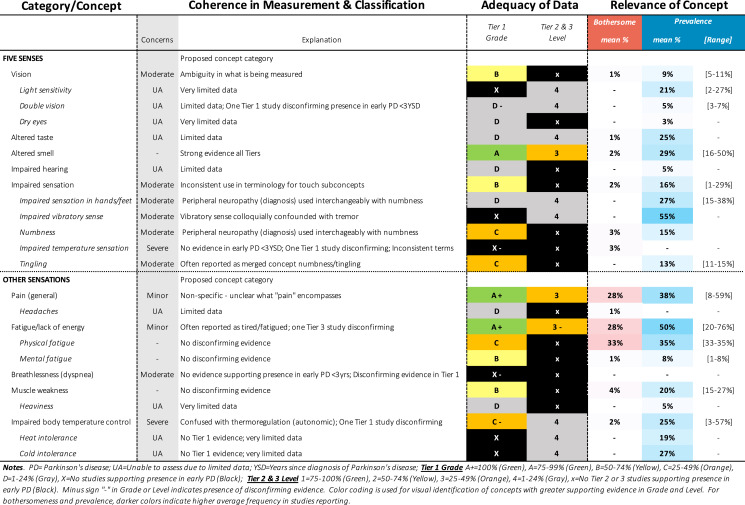
SOFT Report - Sensory Domain

**Table 5:**
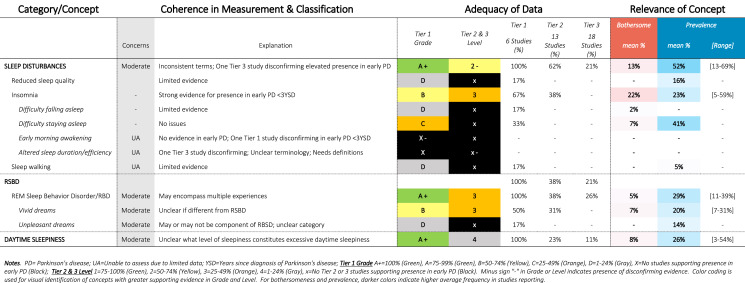
SOFT Report - Speech Domain

**Table 6:**
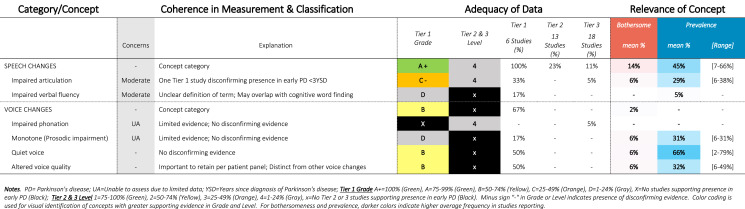
SOFT Report - Sleep Domain

**Table 7:**
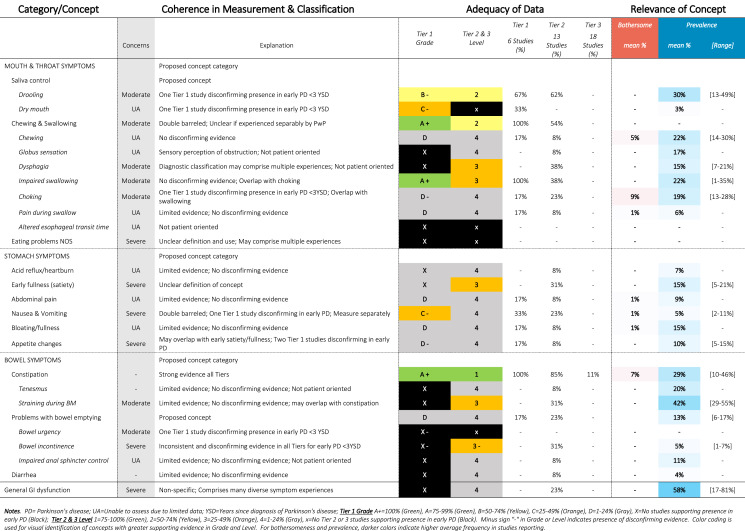
SOFT Report - Digestive Domain

**Table 8:**
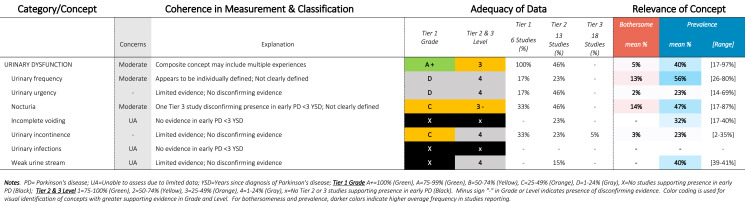
SOFT Report - Urinary Domain

**Table 9:**
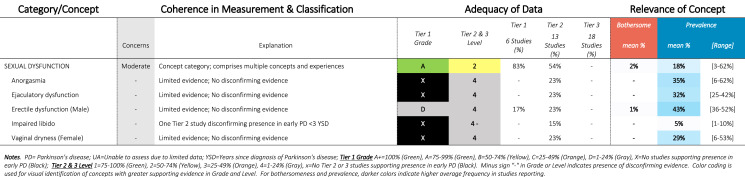
SOFT Report - Sexual Domain

**Table 10:**
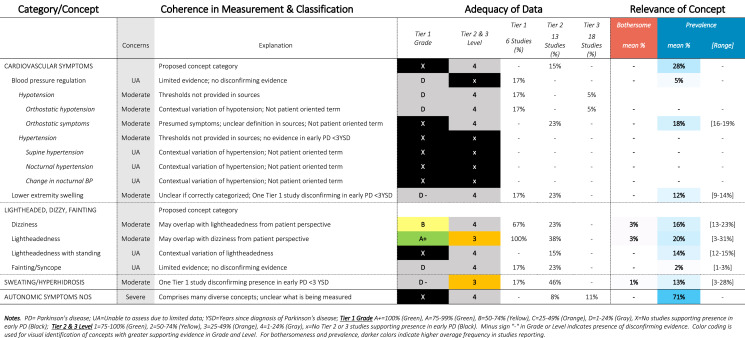
SOFT Report - Autonomic Domain

**Table 11:**
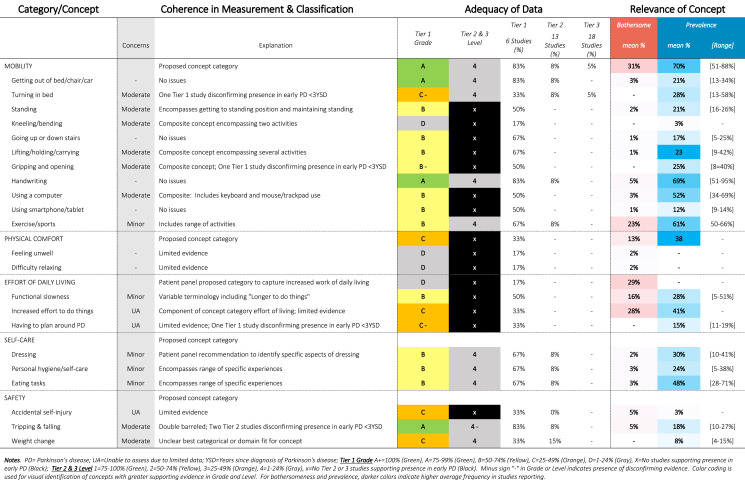
SOFT Report - Physical Functioning Domain

**Table 12:**
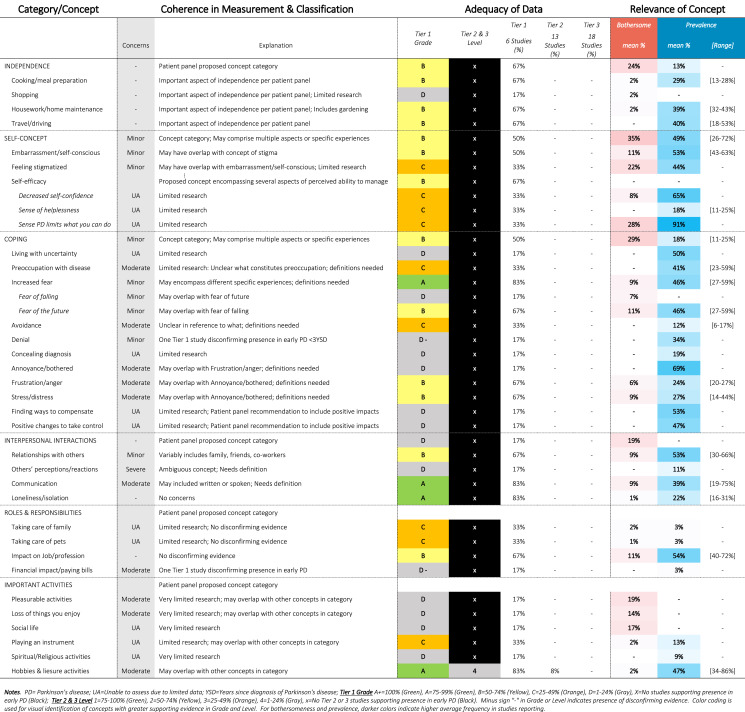
SOFT Report - Psychosocial Functioning Domain

### Consensus conceptual model schema

**Fig. 1:**
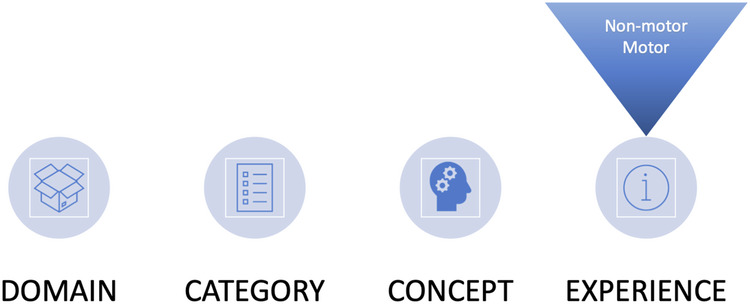
Conceptual model schema.

**Fig. 2:**
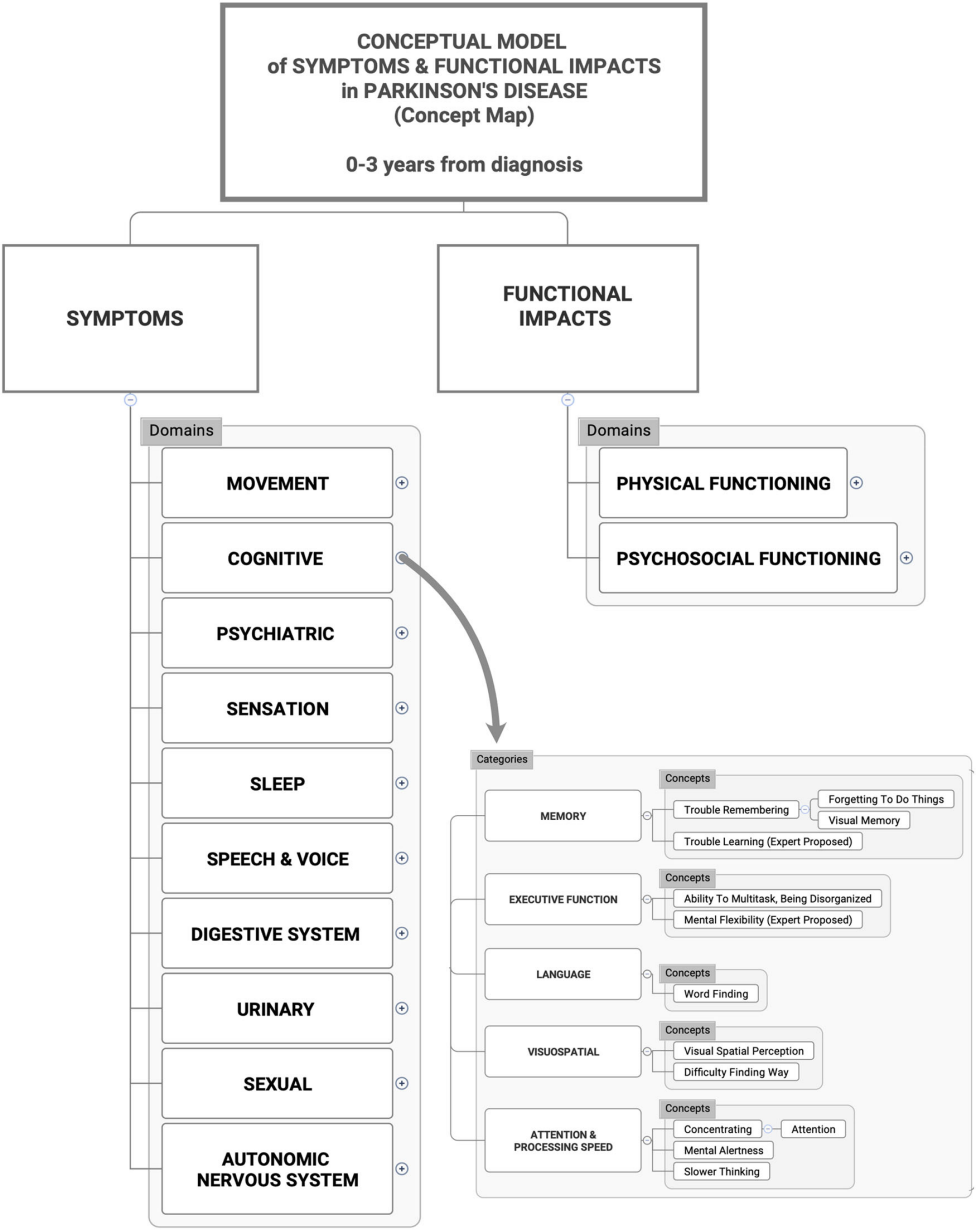
Map of conceptual model domains.

Concepts were organized using a primary classification schema of **Domain—Category—Concept—Experience**, with secondary classification of motor or non-motor occurring at the measurement level, as depicted in Fig. 1. Ten systems-based symptom domains were identified (Movement, Cognitive, Psychiatric, Sleep, Sensory, Speech, Digestive, Urinary, Sexual, Autonomic) in addition to two impact domains (Physical functioning; Psychosocial functioning). A sample map of the top-level conceptual schema is presented in Fig. 2 for the cognitive domain, with individual concept maps for each domain presented in Supplementary Figs. 1–14. Comprehensive data tables including the frequencies by source are presented in the Supplementary Data. Working definitions for each symptom and impact concept in the model are presented in Supplementary Tables 1 and 2.

### SOFT reports

Synthesis of Findings Tables (Tables 1–12) are presented for each symptom by domain. SOFT reports show (1) issues of coherence in measurement and classification of concepts; (2) adequacy of data supporting conclusions; and (3) the relevance of each concept based on prevalence and the extent to which the concept was bothersome in early PD < 3 years since diagnosis. From the SOFT reports, the most meaningful motor symptoms of early PD appeared to include tremor, fine motor difficulties, gait & balance changes, stiffness, and slow movements—all of which were observed to be prevalent (54–85%) and bothersome (24–57%) within 3 years from diagnosis. The SOFT reports also highlighted multiple non-motor symptoms that were common and important to people with early disease. These included cognitive and speech changes (e.g., word finding); mood changes such as anxiety, depressed mood, or negative feelings/emotions; alterations in sleep; sensory changes (e.g., increased pain and fatigue); urinary dysfunction; and digestive system changes (e.g., choking, constipation).

In addition to identifying common symptoms and impacts, multiple gap areas were observed, most often in the impact domains. Early evidence suggests that impact on mobility-related activities, such as physical exercise, may be a high priority area in early PD (70% prevalence; 31% bothersome). Other concepts, such as “Effort of Living” were comparatively new, with no data on prevalence (Tier 1B, 2, 3) but good Tier 1 A evidence (29% bothersome). Other concepts that may be relevant at this stage include impacts on self-concept (35% bothersome), personal coping (29% bothersome), interpersonal interactions (e.g., relationships with others), sense of independence, profession, and hobbies—among others.

## Discussion

The consensus conceptual model presented here is the most comprehensive catalogue of meaningful symptoms and impacts in PD, based on literature to date. It is also the first study to provide evidence-based SOFT report cards with a range of metrics about key concepts by domain, which will be helpful for patients, clinicians, and researchers. This effort expands on prior models derived from individual studies^4,10,12,13^. Corresponding with a recent systematic review by Tosin et al.^99^, we found strongest support for movement, cognitive, mood, speech, and sleep-related symptoms. Top motor symptoms were tremor and fine motor difficulties, followed by gait, stiffness, and slowed movements. Of non-motor symptoms, sleep changes, fatigue, anxiety, slowed thinking, voice/speech changes, and trouble word finding were common. Cumulatively, our findings suggest future treatments and clinical trials might need to address concepts from more than one domain to adequately reflect early PD experiences. Yet, while providing evidence in support of established concepts, this report highlights multiple concepts that appear meaningful but insufficiently studied, as was evident from the SOFT reports. Thus, further research will be needed before definitive conclusions can be made as to which concepts are most universally relevant at this stage. Ultimately, reevaluation of existing outcome measures will be needed to determine the extent to which these measures reflect what matters in early PD.

We believe the SOFT reports presented here can support the evidence-based selection of concepts for research or clinical assessment. However, these reports should be used thoughtfully due to limitations inherent in sampling, data collection, and reporting processes of primary sources. For example, methodological issues in the primary sources could have resulted in over, under, or inconsistent reporting, which in turn would have affected aggregated frequencies. Characterization of samples using H&Y and MDS-UPDRS might lack sensitivity in early disease, particularly with respect to non-motor symptoms^100–102^. Other factors, such as narrow age ranges (57–68 years), low frequency of medication use, underlying disease severity, or even participation in clinical trials may differ from standard clinical populations. Thus, the term “SOFT report” is intentional and serves as a reminder that data are not conclusive and should be treated as an estimation rather than an exact measurement. Future work will help achieve more precise understanding of concepts and a parsimonious set of core outcomes for early-PD.

At present, many gap areas exist, where concepts had insufficient data to justify inclusion or exclusion in the final model. It is important to note that absence of supporting data does not indicate a concept is not meaningful. For instance, “gait changes” appears highly relevant in early PD, with little data to indicate which aspects of walking are problematic. In contrast, disconfirming evidence alongside confirming evidence raises questions about concept cohesiveness and adequacy of prior measurement, as was found with reports of dyskinesia in early PD. Thus, particular attention should be paid to concepts with limited or discrepant evidence, to define scope and determine relevance. Identifying specific experiences related to concepts will lead to greater consistency in concept definitions and downstream measurement approaches.

For the reasons discussed above, no single metric in the SOFT reports should be used in isolation to justify selection of COI. Consideration should be given to all metrics, including estimates of prevalence, evidence of relevance in early PD, and total weight of evidence justifying conclusions. Linking symptom concepts to specific functional impairments might also be helpful, allowing for triangulated assessments (e.g., what the person can or cannot do because of fatigue). Other considerations, such as measurability, expectations for change within the time frame of the trial (e.g., 6–18 months), anticipated susceptibility to treatment, and context of use are also important. For example, “stigma” may be relevant to PwP, but unlikely to change rapidly with treatment. Other important points to consider are universality and participant characteristics such as age, life-stage, sex, gender, geographic location, and culture, which can alter the meaningfulness of concepts. For example, the impact of PD on driving may be different for people in rural areas than for urban residents, which could affect the suitability of the concept for geographically diverse trials. Similarly, COIs may be sex specific (e.g., erectile dysfunction) and not equally applicable to both sexes. It is also important to consider normative values, social biases, and recall. Some experiences may be difficult to remember accurately over time, while others may be subject to social desirability bias (e.g., reluctance to report compulsive behaviors). Ability to tell if a symptom is attributable to PD is also important, as was highlighted by the patient panel.

Key strengths of this study include exhaustive review and meta-synthesis of diverse studies, inclusion of stakeholders throughout the model-building process, and use of an iterative, consensus-based design. We believe this has contributed to a maximally inclusive model reflective of current science, with an intuitive and easily understood interface, which will contribute to clinical care and early PD trials. However, several areas remain in need of substantial future work. Foremost, there remains a marked lack of diversity in PD research^103^. We were unable to evaluate similarities or differences in symptoms and impacts between age groups, sexes, or cultures. All Tier 1 evidence was elicited from the UK, USA, and Canada, with samples that were >93% white, with only marginally greater diversity in Tier 2 and 3. Future work should aim to expand knowledge of meaningful symptoms and functional impacts in culturally, geographically, racially, sex, and gender diverse populations^6–9^. Research to understand the impact of early PD on patients and families might also be warranted, as only one study in the review reported the perspectives of family members separately from patient perspectives^16^.

A second call to action is for harmonization of concepts and concept definitions, which is needed for synergy in future work. However, the schema proposed here is not intended to be prescriptive, but to support use, consistency, and forward momentum. As such, all terms in the present model have been given working definitions (Supplementary Tables 1–2) derived from evidence-based resources or common language usage^104–106^. It is expected this will evolve over time and that both model schema and definitions may require revision or refinement as the field matures. Revisions should be made cautiously and grounded in rigorous evidence, with careful attention to existing items to minimize redundancy. Where possible, researchers will benefit by building on prior work. When needed, clear and compelling justification should be provided for alternate terms. In selecting “best” terms and groupings, reflecting the experiences of the people living with PD should remain the top priority. For example, autonomic symptoms often overlapped with other categories (e.g., Gastrointestinal). Where this occurred, domain assignment was determined by consensus (patient + expert) with preference to patient-friendly groupings. Thus, where possible, lay-friendly terms will be preferred over complex technical terms (e.g., slow movements vs. bradykinesia).

Finally, in addition to refinement of concepts, intermittent re-evaluation will be needed to ensure alignment with emerging biological staging systems for neuronal synuclein disease (NSD)^107^. In the present model, studies relied on time since clinical PD diagnosis, and findings might not be fully translatable to biologically-staged NSD. Best approximation is likely to NSD Stage 3 or 4 (mild symptoms and slight to mild functional impairment)^107^. As such, the proposed model should be understood as “best fit now” to prevent loss of momentum while striving for increasing harmonization. Future work will enable better understanding of what is important at each stage and selection of stage-appropriate COI and COA for clinical trials.

In conclusion, a widely accepted consensus conceptual model is an essential step in development of meaningful and reliable fit-for-purpose COAs for clinical trials in early PD. Future work should aim to reevaluate the adequacy of existing measures to capture what matters to PwP and their families based on this new evidence. Collaborative efforts, leveraging prior work, and consensus on key concepts will be crucial to advancing the science, reducing effort duplication, and ultimately developing the disease-modifying treatments that are so desperately needed. We believe the methods and findings described here will help address gaps in outcome measure development and serve as an exemplar for future conceptual model development beyond PD.

## Methods

**Fig. 3:**
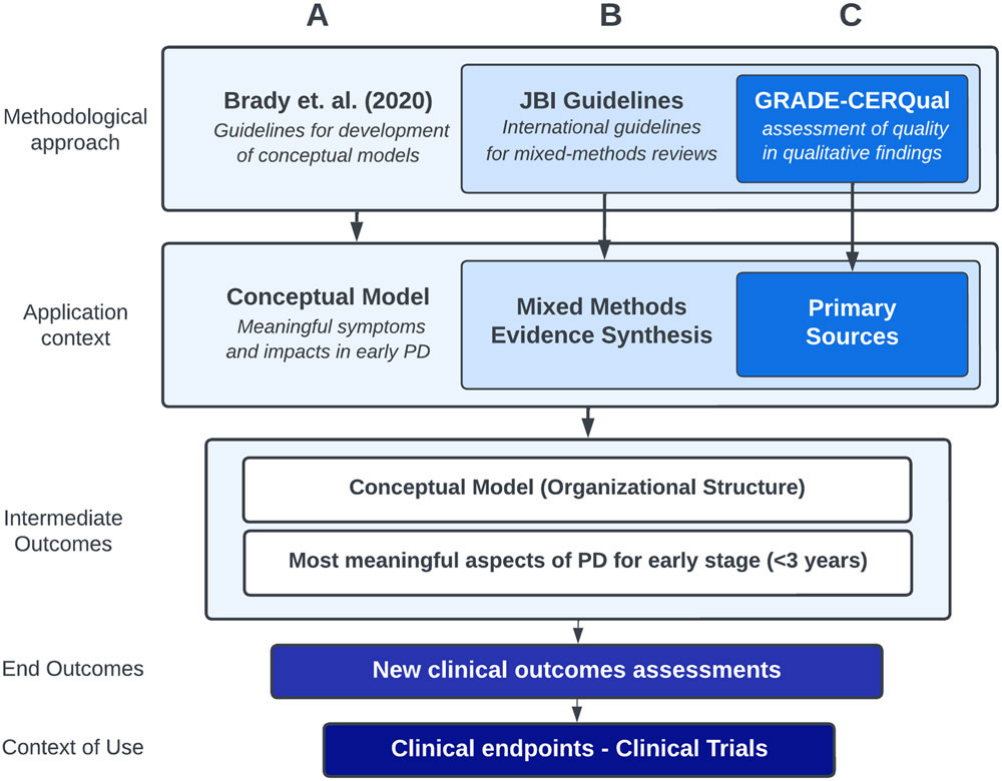
Approach to development of the consensus model.

Best-practice guidelines were followed for each stage of model building, including systematic review of the literature, mixed-methods evidence synthesis, and assessment of evidence quality^108–115^. These are shown in Fig. 3 and described below.

### Approach to systematic review & model development

Guidelines proposed by Brady et al.^109^ were used for the development of conceptual models, which entailed: (1) identifying resources (e.g., existing models, stakeholders, and literature-based sources), (2) considering the broad array of possible factors identified from resources, (3) narrowing down factors for inclusion on the basis of theory, stakeholder perspectives, and evidence^109^. A 14-person, multi-stakeholder taskforce convened March of 2023, together with a 9-person patient and family advisory panel (Table 13), to develop the methods and approach to the systematic review of the literature (Step 1). Stakeholders included people affected by PD (patients and families), researchers, clinicians, PD advocacy groups, industry, and regulatory agencies (FDA). The purpose of the literature review was to identify all reported symptoms and functional impacts of early PD (Step 2), with ultimate intent to identify which concepts are most meaningful for early-stage disease, to inform the final model (Step 3).

**Table 13:** Stakeholder participation in development of the consensus conceptual model

Stakeholder group	Investment
**People affected by PD** • Patients • Families (Spouse/partners, children, close friends)	• ***Taskforce member***: PwP representative at monthly taskforce meetings to consult on approach, progress, and co-author final product • ***Advisory panel*:** Ethnically and gender diverse 9-member patient advisory panel convened to (A) review and advise on approach developed by taskforce; (B) provide iterative real-time feedback on model structure, terminology, presentation, and potential uses; (C) review and provide feedback on final manuscript and results; (D) co-authors on final manuscript. Panel characteristics:
**Clinicians** (Neurology, PCP, Nursing, Speech/PT)	**Multi-stakeholder taskforce convened monthly to:** • Co-develop approach • Monitor progress • Advise on data extraction and meta-synthesis • Critique and iteratively revise model and outputs • Co-author presentations, publications **Public Review Period** • One-month public review period to provide feedback on final report.
**Researchers** • Model developers; PD staging experts, Topical experts	
**PD Advocacy Groups** • Michael J Fox Foundation • Parkinson’s UK	
**Industry** • Roche, AbbVie, GSK, Roche, Denali, UCB	
**Representatives of professional agencies** • Critical Path for Parkinson’s • Movement Disorders Society	
**Regulatory** • FDA • National Council on Aging • CMS (Center for Medicare Services)	• Review and provide feedback on draft methods and results. • Review and provide feedback on final report

The taskforce elected a *convergent integrated synthesis* approach to identify meaningful symptoms and impacts in both qualitative and quantitative studies^108,116,117^. For the review, JBI Mixed Methods Review criteria^108,117^ were used to: (1) define the review question; (2) determine inclusion/exclusion criteria; (3) define the search strategy (4) systematically assess methodological quality (5) perform data extraction (6) synthesize data; and (7) present results.

### Research question: What symptoms and impacts are most meaningful in early PD?

#### Definitions of key terms

There is no formal definition of early PD and most studies to date have utilized clinical diagnosis, with variable definitions of early-stage disease ranging from 0 to upwards of 6 years. For the model, early PD was defined as less than 3 years since diagnosis (YSD) by expert consensus. The 3-year timeframe aligns with the target population of the Critical Path for Parkinson’s Consortium and with many clinical trials for early PD^118^. People affected by PD were defined as patients/people with PD (PwP) and their intimate social circle, referred to hereafter as “family.” The terms “caregiver” and “care partner” were not used as most people with early PD do not have formal caregivers, and “partner” does not encompass the scope of people affected by PD, such as children and close friends.

A concept of interest was defined as the “aspect of an individual’s clinical, biological, physical, or functional state, or experience that the assessment is intended to capture or reflect”^8^. For this model, “symptoms” were considered to be the subjective or objective physical and mental features (i.e., signs/symptoms of disease) occurring as a direct result of PD, leading or potentially leading to changes in day-to-day physical and psychosocial functioning. “Functional impacts” (hereafter “impacts”) were defined in alignment with FDA guidance as experiences occurring as a consequence of disease, such as changes in the way a person functions or feels^6^. Per patient panel and expert discussion, to be deemed “meaningful” a concept (either symptom or impact) had to show evidence of being prevalent as well as personally bothersome to people with early PD.

### Primary source inclusion/exclusion criteria

Sources were eligible for inclusion if they were: (a) primary published or unpublished qualitative, quantitative or mixed methods (MM) studies; (b) conducted within an early PD population as defined by source study authors; (c) reported any symptoms and/or impacts of early PD; and (d) contained data that were patient, family, observer, clinician reported or digitally measured. For longitudinal studies baseline measurement values were used. Studies focused on evaluating the effect of a specific medication or intervention were excluded, as were conference proceedings.

### Search strategy

For the literature review, any source with a study-defined “early PD” population was included to avoid missing potential sources during the search process. Four databases were searched as shown in Supplementary Table 3. Search ***Strategy 1*** identified sources published within 10 years that referenced early PD and symptoms or impacts anywhere in the title or abstract (search date: May 2023). ***Strategy 2*** identified sources that used early PD and common terms for qualitative research anywhere in the title or abstract *without* time limits. ***Strategy 3*** focused on reference lists of relevant review articles to identify additional sources. ***Strategy 4*** used expert consultation to identify key sources > 10 years old or unpublished relevant datasets not captured in the first two search strategies.

**Fig. 4:**
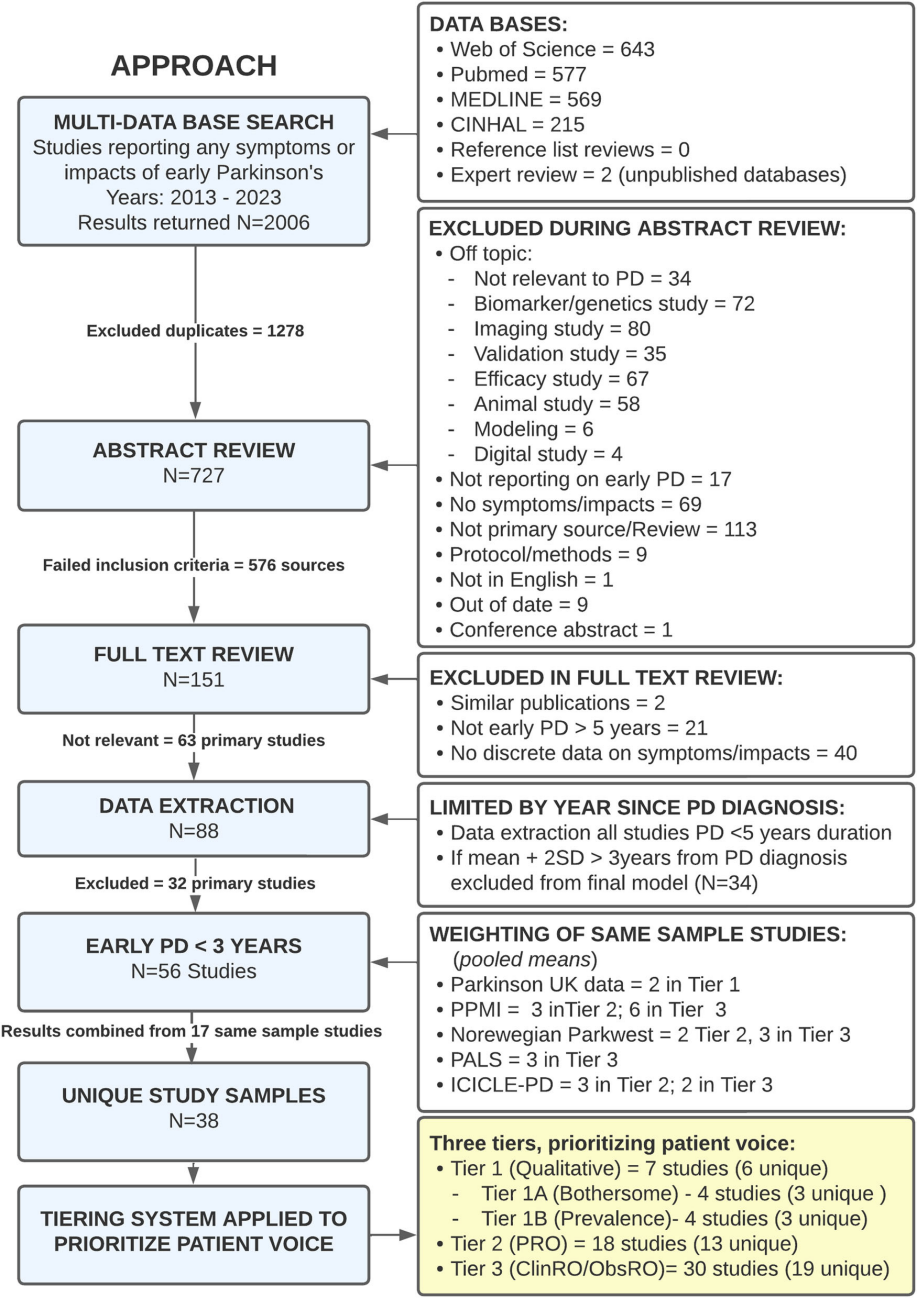
Flowchart for identification and screening of sources included in conceptual model.

As shown in Fig. 4, 2006 sources were returned, with 1301 duplicates. Abstracts were screened for 705 sources. Of these 554 were excluded and 151 were selected for full text review. Eighty-eight sources remained after eliminating studies without reportable data on symptoms or impacts within any early PD population. Of these, only 56 studies used samples that were strictly <3 years since diagnosis based on mean and SD. A complete audit trail of sources screened and included/excluded is provided in **Supplementary References**.

### Approach to data analysis

All sources that met review inclusion criteria were systematically analyzed, and findings were weighted and aggregated to enable assessment of the total quality of evidence supporting each concept in early PD, as described below. Data extraction for *concepts* was performed on all studies of early PD as defined by study authors (*N* = 89; range 0–6 years since diagnosis), however, data regarding *frequencies* of concepts was limited to PD < 3 years since diagnosis (*N* = 56). This was done to maximize identification of potential concepts with reported frequencies specific to early PD.

### Data extraction

For mixed methods synthesis, JBI guidance recommends codifying quantitative data in a manner compatible with qualitative synthesis to reduce potential for inaccuracies in meta-aggregation across methodologies^116^. Using a matrix spreadsheet (Supplementary Data), all studies were assessed individually for study aims, design, year of publication, sample size, PD stage, diagnostic criteria, years since diagnosis (mean, SD), comparison group, gender distribution, race/ethnicity, country of origin, data source, data collection instruments, PD medication use, levodopa equivalent daily dose (LEDD), Hoehn & Yahr (H&Y), Movement Disorders Society Unified Parkinson’s Disease Rating Scale (MDS-UPDRS) part III total score if reported^119^, any covariates, and a brief study synopsis.

Each source was then analyzed to extract information about symptoms or impacts of early PD. Where given, frequencies for prevalence were extracted. For studies lacking frequencies but reporting between groups comparisons for *early PD vs. Control* (e.g., normative cohort, later PD cohort), statistically significant differences were indicated as present (*) and lack of statistically significant differences indicated as not present (-). For studies reporting bothersomeness rather than prevalence, the percentage of people identifying the concept as being actively bothersome was reported.

### Development of the conceptual model schema

After content coding, all identified concepts were qualitatively analyzed to derive a best-fit conceptual schema that was intuitive, parsimonious, supported measurement consistency and reduced redundancy. Only concepts identified by systematic review were included in the modeling. Initial attempts to group by motor versus non-motor resulted in a poorly organized structure due to the large number of concepts identified and the presence of many concepts with ambiguous classification (e.g., motor vs. non-motor; symptom vs. impact). A series of 10 interactive sessions were held from September to November 2023, to solicit feedback from all stakeholders (taskforce and patient panel) and derive a consensus-based schema that was intuitive and user-friendly for clinicians, researchers, PwP and families. Sessions were held online in focus group format, with a moderator who summarized and synthesized perspectives in real time.

Using mapping approaches (Xmind) with screen sharing, concepts were clustered by relatedness and organized into logical groupings^120,121^. Where possible, full agreement was sought for all analytic decisions, with use of >80% majority vote on best groupings when 100% consensus was not achieved in online meetings. Similar concepts were merged and consensus term selected based on taskforce and patient panel agreement. Concepts that related to a broader concept were subsumed as dependent nodes to develop a branching structure moving from broad concepts to progressively more specific aspects of an experience (e.g., shuffling is an aspect of gait). All conceptually distinct items were retained in the final model schema (Supplementary Figs. 1 through 14). Conceptual distinctness and relatedness were determined by stakeholder consensus. Detailed documentation of stakeholder sessions and revisions to the schema was retained for an audit trail.

### Weighting of primary sources preparatory to metasynthesis

The PFDD guidance series prioritizes *direct report* of patient experience from the target population^9^. When this type of data is limited or the patient population has reduced ability to reliably report experiences, supporting information may be obtained from caregivers, clinicians, or other key informants^8^. Based on discussion with the patient panel and taskforce members, a three-tiered approach was chosen for classification of primary sources. This was done to allow for prioritization of patient voice and weighted synthesis of findings across diverse methodologies and data sources as described below.

Tier 1^4,10–13,15–17^ comprised qualitative or mixed methods studies that evaluated symptoms and impacts of PD using an open-ended, iterative, and patient-driven approach, in which patients and/or family were asked to freely identify what symptoms or impacts the person experienced without any constraints. Tier 1 was further subdivided to ***Tier 1*** ***A*** (studies reporting a symptom as being *bothersome* in early PD irrespective of prevalence) and ***Tier 1B*** (studies reporting symptoms as *present* in early PD irrespective of whether it is bothersome). Tier 1 sources were used as *primary evidence* for the conceptual model. Original study teams from Tier 1 sources were contacted to obtain detailed frequencies for symptoms and impacts if not fully presented in published manuscripts^4,10–12,16,122^.

Tier 2 and 3 consisted of quantitative studies in which predetermined aspects of health were measured using quantitative approaches. ***Tier 2*** included studies that evaluated symptoms and impacts using patient-reported outcome (PRO) measures, in which a limited selection of symptoms or impacts were evaluated from the patient perspective^22,23,27,33,34,36,50,52,58,60,62,66,68,72,74,77,79,81,82,84,85,87,88,90–92,94^. ***Tier 3*** included data from studies with clinician or observer reported symptoms or impacts (i.e., ClinRO, ObsRO)^18–21,24–26,28–32,35,37–49,51,53–57,59,61,63–65,67,69–71,73,75,76,78,80,83,86,89,93,95–97^. Sources reporting only cumulative scores on validated scales were excluded as they lacked discrete data on symptoms or impacts. Tier2 and 3 studies were included as *supporting evidence* due to potential for bias in symptom reporting.

#### Pooling of same sample studies

Same sample studies were defined as separate publications that reported findings from the same (identical) participant sample (Supplementary Data). Findings were pooled from same-sample studies to ensure equal weighting of concepts during meta-synthesis. For pooling, redundant findings (e.g., demographics—diagnosis of depression) were reported once, while all unique findings were retained. Thus, a total of 38 unique study samples were included in the final model.

### Aggregation of data for early PD < 3 years since diagnosis

Data aggregation was performed at the level of *unique samples* (*N* = 38), rather than at the level of individual studies so that each unique sample was represented only once in the final meta-synthesis. Only samples with data for PD < 3 years since diagnosis were included at this stage, based on the final model inclusion criteria. Data and frequencies for the full early PD sample (*N* = 89, 0–6 years since diagnosis) vs. PD < 3 years since diagnosis can be viewed in Supplementary Data.

The following metrics were calculated for each symptom and impact in early PD < 3 years since diagnosis:

1. Number and percentage of unique samples that measured a concept (within and across Tiers);
2. Average prevalence of concept (within and across Tiers 1B, 2, & 3—calculated as the sum of frequencies in all studies reporting prevalence/total number of studies reporting prevalence);
3. Number and percentage of unique samples disconfirming presence of concept (within and across all Tiers); and
4. Frequency which concept was reported as being actively bothersome (Tier 1 A; calculated as the sum of frequencies in studies reporting bothersomeness/total number of studies reporting bothersomeness).

### Assessment of quality of evidence and synthesis of findings

Next, evidence synthesis and assessment of quality was performed using GRADE-CERQual^110–115^. GRADE-CERQual is a standardized approach to assessment of confidence in the quality of evidence from qualitative studies and is endorsed by the World Health Organization and numerous government agencies for the development guidelines to shape public policy and research^123–125^. CERQual evaluates four primary areas: (1) methodological limitations, (2) coherence of findings, (3) adequacy of the data, and (4) relevance of the findings. Operationalized criteria for this are presented in Table 13. Methodological limitations were addressed via the Tiered approach, in which findings were weighted by methodology.

### Research community review

Lastly, to maximize potential for usefulness and adoption of the consensus model, the model and manuscript and all supporting files were posted online for research community review and feedback over a 1-month period (Jan-Feb 2024). Participants for the community review were solicited via the MJFF research community newsletter (distribution list *N* = ~9.5 K), the UK PD clinical Studies Group (*N* = 65) and by personal invitation to experts identified by the taskforce members, including members of FDA, EMA, MDS, CPP and content experts (*N* = 88). An online form was provided for feedback or feedback could alternately be emailed directly to taskforce members. Final minor clarifications were made to the model, figures, and manuscript on the basis of reviews, with an audit trail of changes available upon request Table 14.

**Table 14:** Operationalized criteria for assessment of quality of evidence – GRADE-CERQual

CERQual Criteria	Operationalized definition	Approach to criteria	
**Methodological limitations**	Methodological limitations in approaches to identifying symptoms and impacts that may limit what type of information was reported—e.g. use of validated measures that only collect specific data (restricted), vs. qualitative interviews allow for unrestricted reporting.	Tiering system for weighted inclusion of sources by methodological adequacy: • Tier 1: Qualitative studies collecting unrestricted data directly from report of patients and family. • Tier 2: Quantitative studies measuring specific symptoms or impacts predominantly from the patient perspective. • Tier 3: Quantitative studies measure specific symptoms or impacts from the clinician or outside observer perspective. Highest priority is given to direct patient voice with unrestricted approach to exploring symptoms or impacts. Lower priority is given to Tier 2 and 3 sources due to methodology that resulted in restricted data collection.	
**Coherence**	Assessment of the agreement of the primary studies regarding the concepts of interest. Threats to coherence include: contradictory data, divergent classification, ambiguous or conflicting descriptions.	• No concerns—consistent and coherent classification of COI across studies • Minor concerns—80–94%% coherence in classification • Moderate concerns—50–74% coherence in classification • Severe concerns—<50% coherence in classification No or minor concerns preferred.	
**Adequacy**	Adequacy is the richness and quantity of data supporting the measurable presence of a COI in the early PD population as seen in primary sources. *Primarily*: Percentage of Tier 1 sources (direct patient voice) that reported the COI as being measurably present in an early PD population. *Secondarily*: Percentage of Tiers 2 and 3 that reported the COI as being measurably present in an early PD population. Evidence from Tiers 2 and 3 alone is *insufficient proof* of adequacy if lacking evidence in Tier 1.	*Primary classification of adequacy (Tier 1):* • Grade A = Strong evidence in Tier 1 • Grade B = Moderate evidence in Tier 1 • Grade C = Limited evidence in Tier 1 • Grade D = lacking evidence in Tier 1 • Grade X = No evidence in Tier 1 *Secondary classification of adequacy (Tier 2 & 3):* • Level 1 = strong evidence in Tier 2 or 3 • Level 2 = moderate evidence in Tier 2 or 3 • Level 3 = limited evidence in Tier 2 or 3 • Level 4 = very limited evidence in Tier 2 or 3 • Level x = No evidence in Tier 2 or 3 Grade A and B evidence preferred (ex. A1, A2).	*Thresholds are based on thepercentage of studies* *reporting the concept:* • X/x = No studies reporting (Black) • 1–24% of studies = Very limited evidence (Gray) • 25–49% of studies = Limited evidence (Orange) • 50–74% of studies = Moderate evidence (Yellow) • ≥ 75% of studies = Strong evidence (Green) Color coding is used for easy visual identification of concepts with greater supporting evidence. Grade vs. Level is used to distinguish between Tier 1 vs. Tier 2 & 3 evidence. Grade and Level do not indicate the prevalence or relative bothersomeness of a concept. Concepts with low grade or level may be understudied important concepts, particularly if any evidence for bothersomeness or higher prevalence.
**Relevance**	Composite score indicating the extent to which evidence from the primary studies supports the concept as being actively bothersome to people with early PD, in addition to being commonly present in the population.	*Bothersome rating*: Average frequency (%) at which concept is reported as bothersome in early PD among studies measuring frequency of the concept. *Prevalence rating*: Estimate of presence of concept *in an early PD population* based on the average prevalence (%, Range) reported in studies that the measured the construct.	

## Supplementary information

Supplemental material - Consensus conceptual model early PD

Supporting data for consensus conceptual model of early PD

## Data Availability

Data for the consensus model literature review are included in Supplementary materials.
