# Supplementary material for: Systematic review and consensus conceptual model of meaningful symptoms and functional impacts in early Parkinson’s Disease
Source: NPJ Parkinsons Dis. 2025 Apr 3;11:65. doi: 10.1038/s41531-025-00907-2 (PMC11965473; doi:10.1038/s41531-025-00907-2)
Supplement: Supplementary file 1 — Supplemental material - Consensus conceptual model early PD [file 41531_2025_907_MOESM1_ESM.pdf]

Supplementary Figure 1. Domain Level

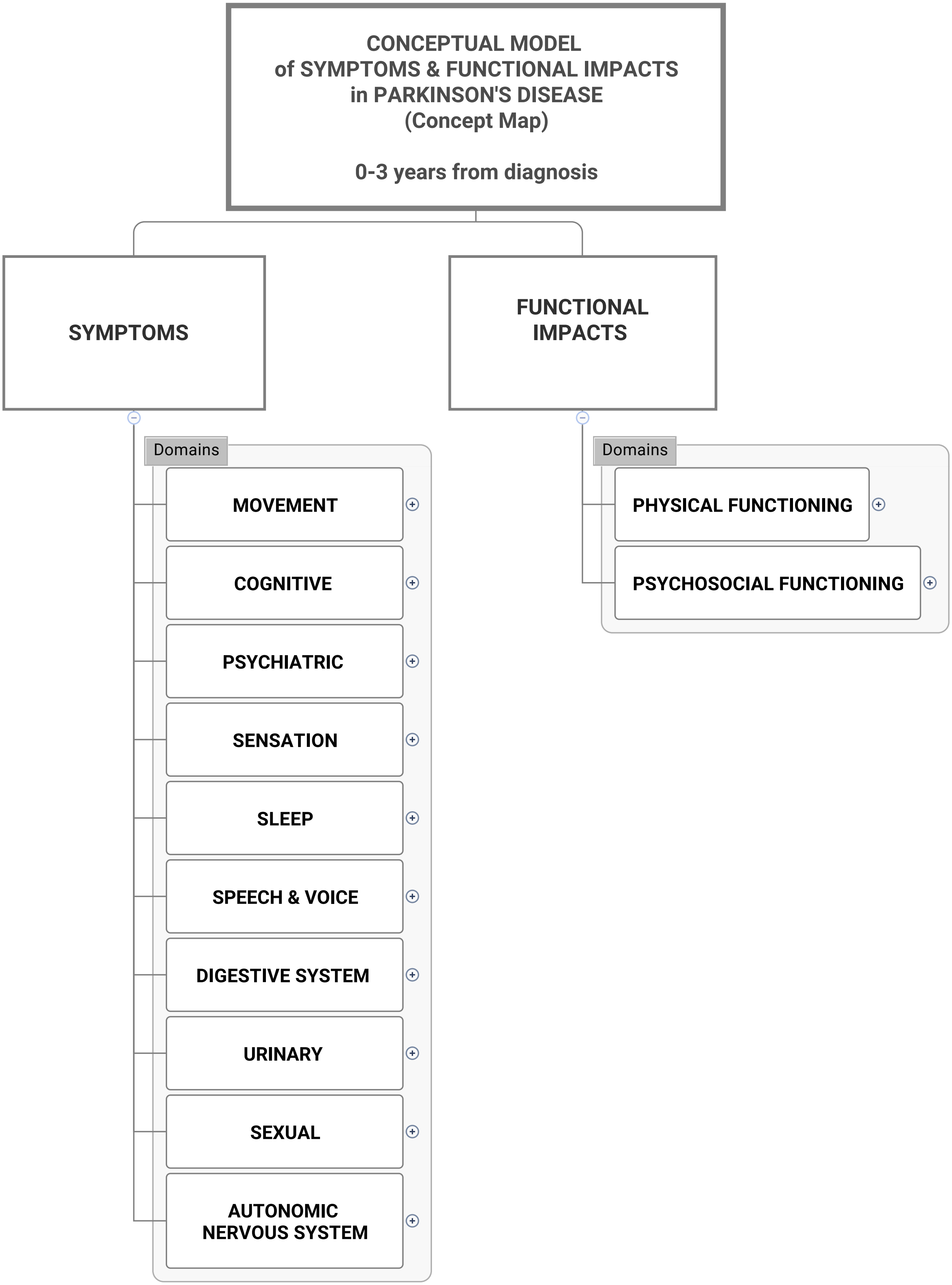

Supplementary Figure 2. Movement Domain

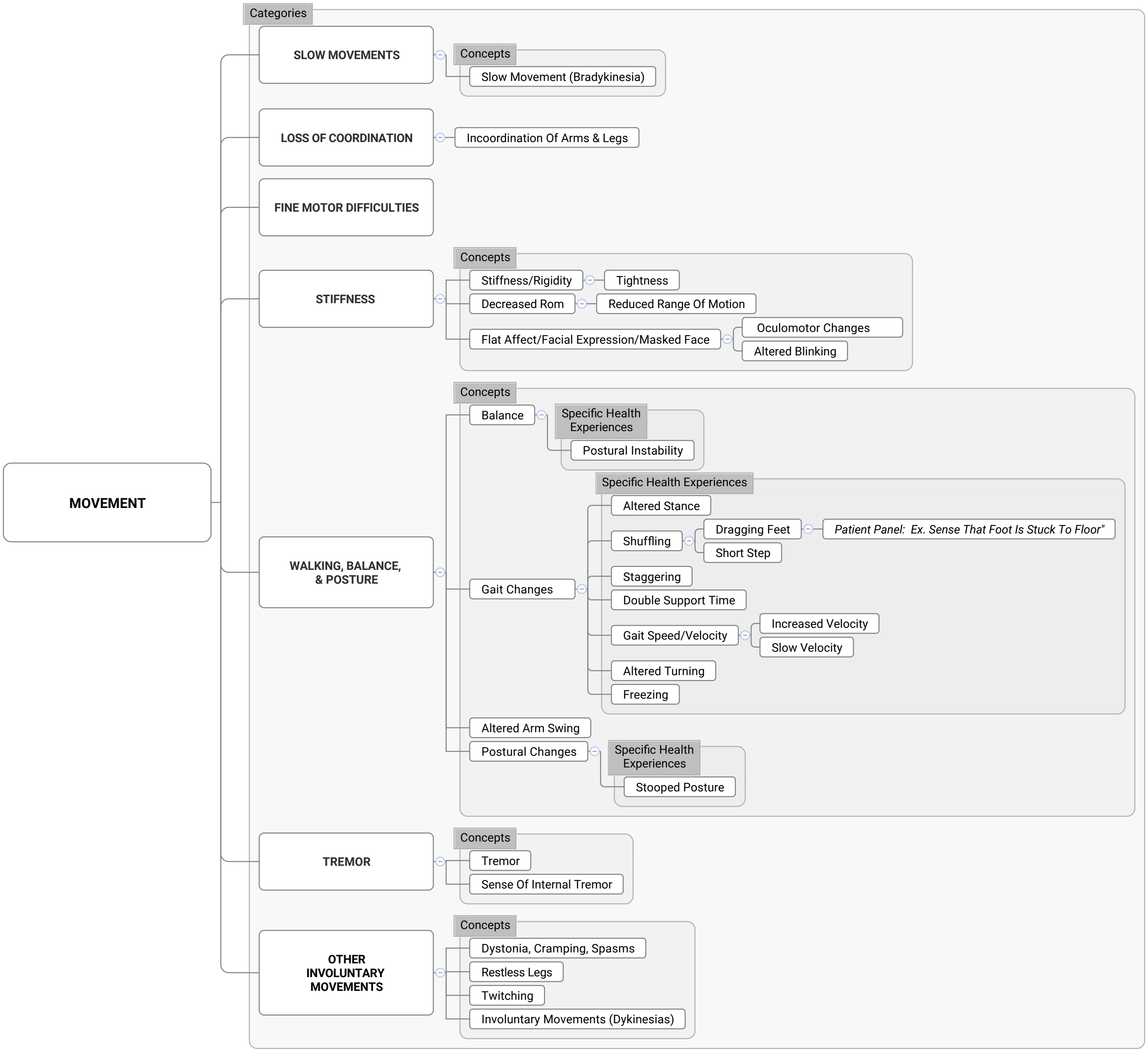

Supplementary Figure 3. Cognitive Domain

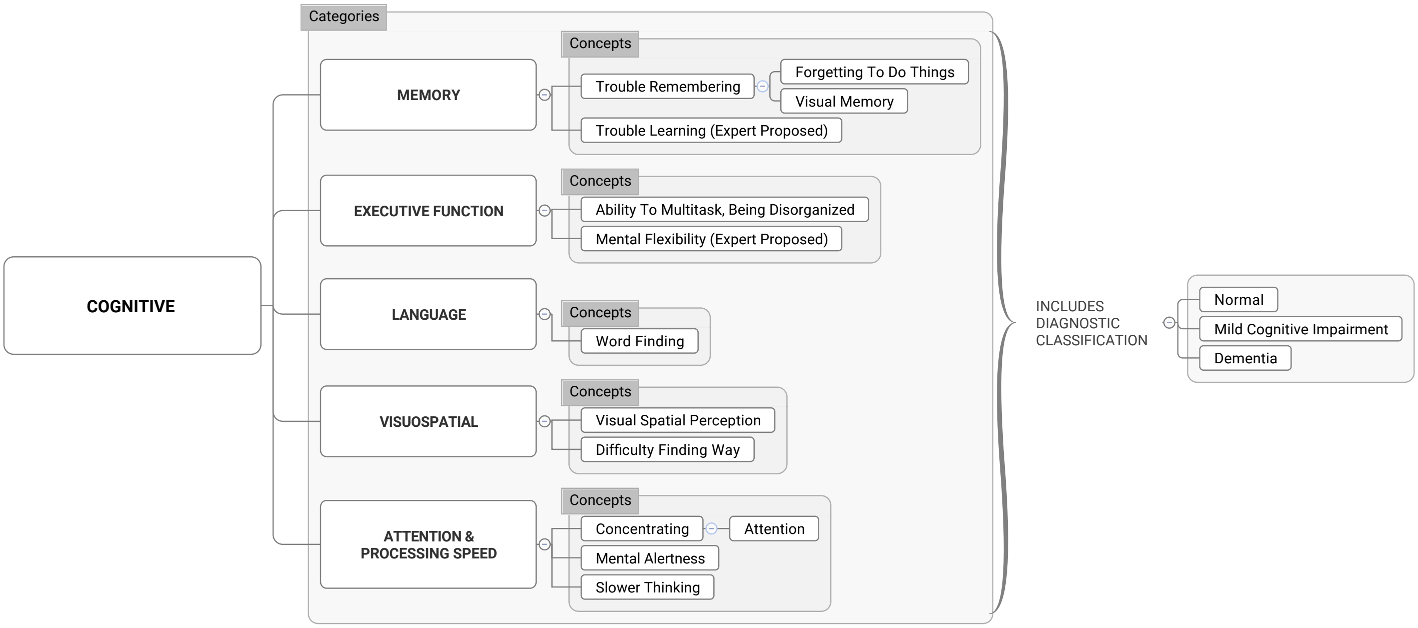

Supplementary Figure 4. Psychiatric Domain

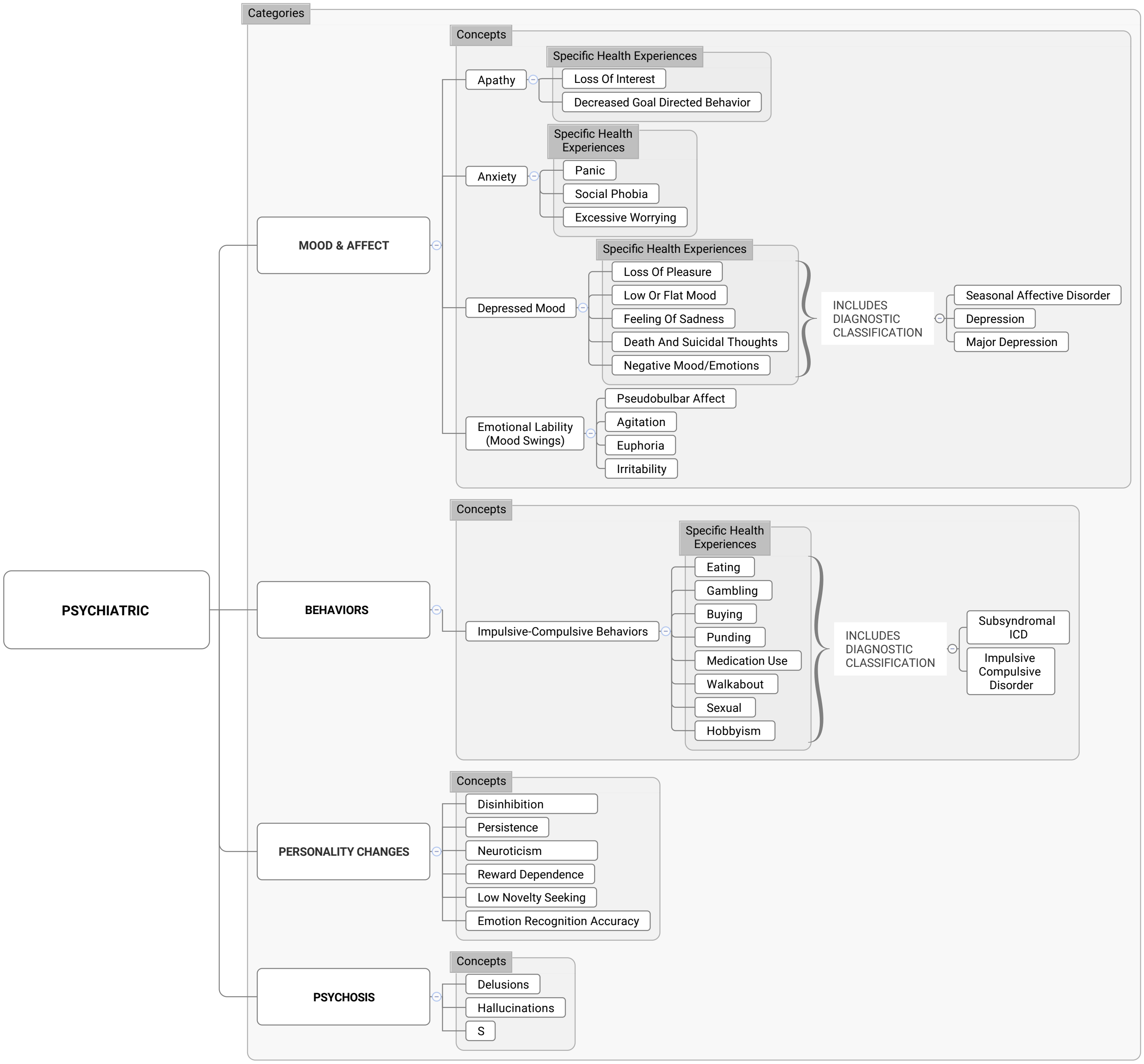

Supplementary Figure 5. Sensation Domain

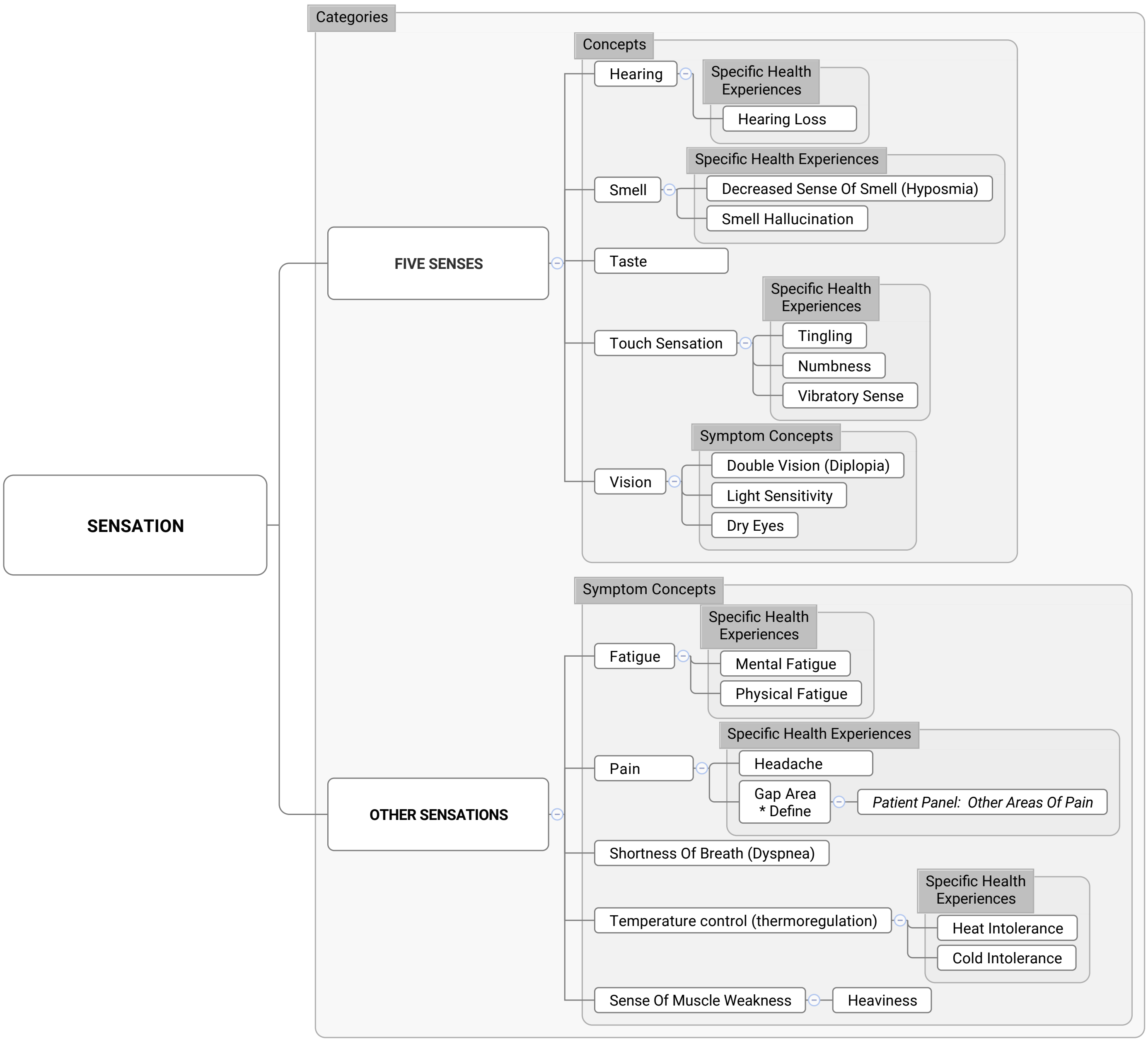

Supplementary Figure 6. Sleep Domain

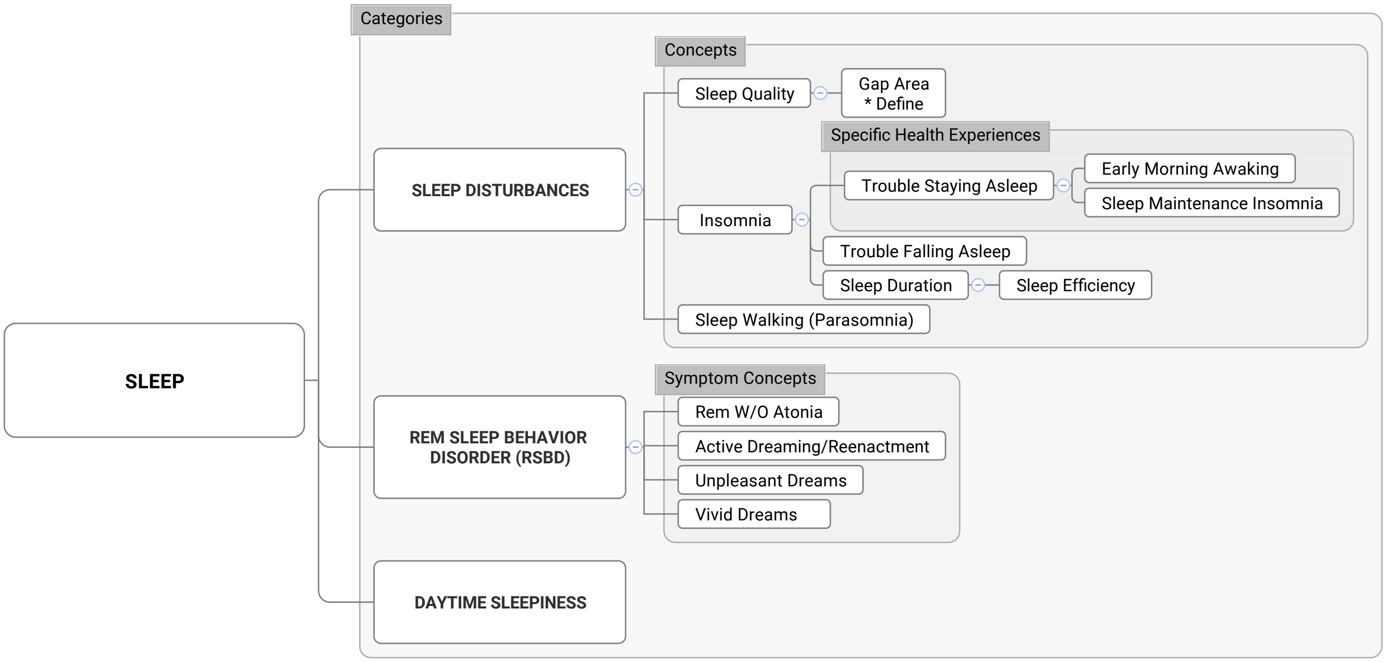

Supplementary Figure 7. Speech & Voice Domain

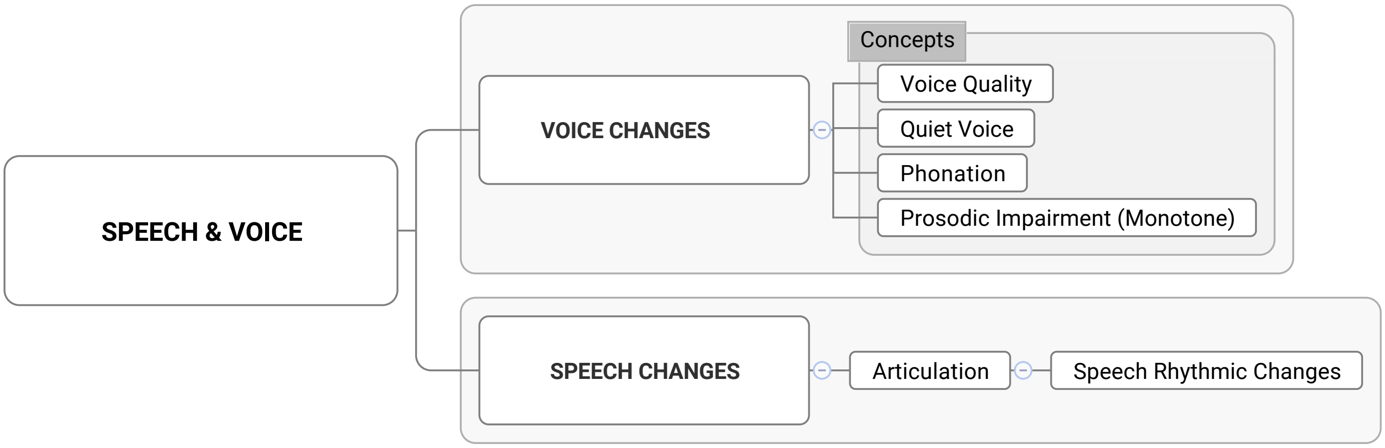

Supplementary Figure 8. Digestive Domain

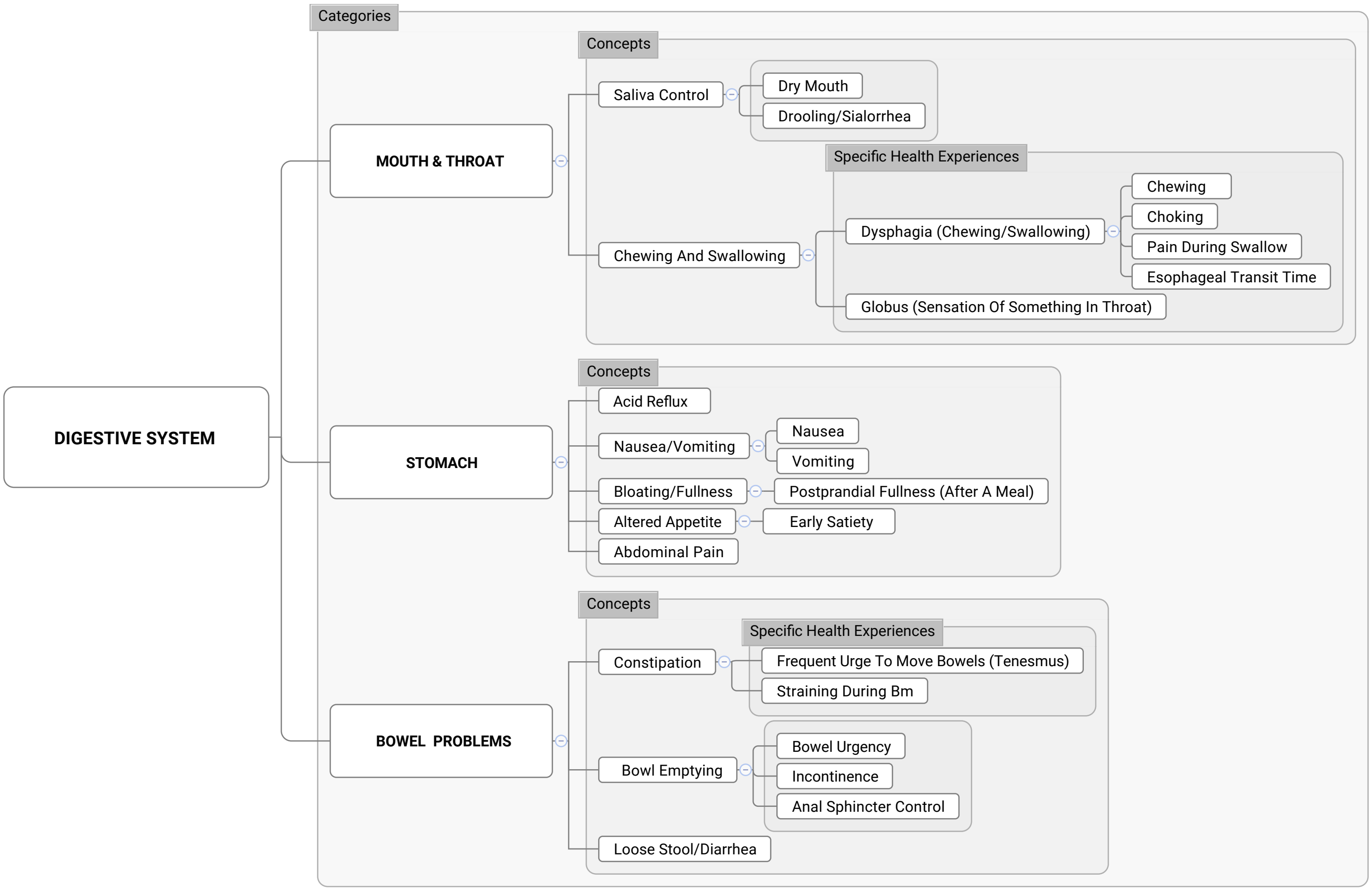

Supplementary Figure 9. Urinary Domain

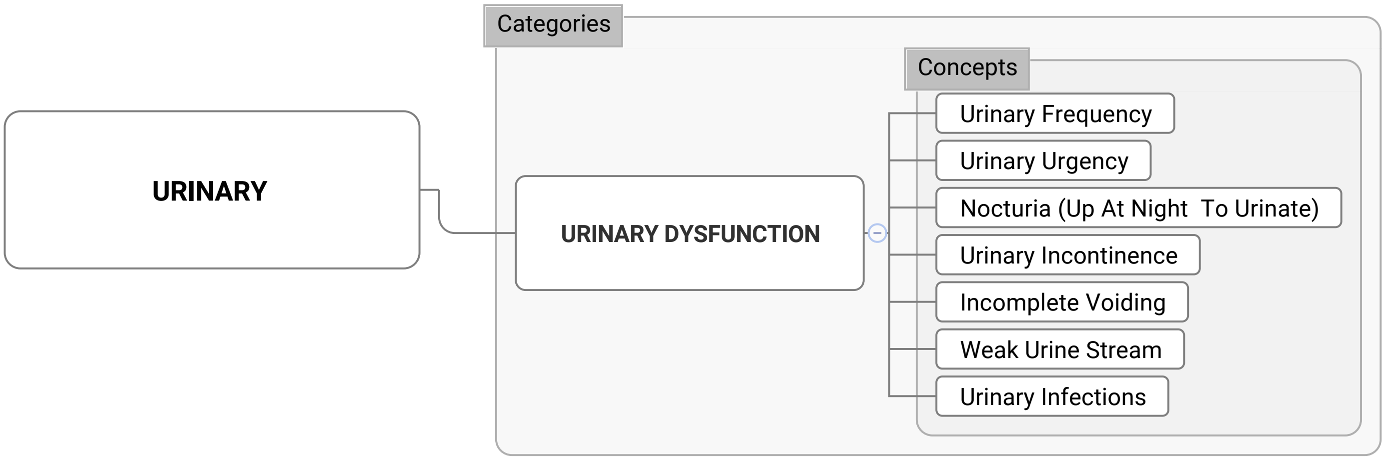

Supplementary Figure 10. Sexual Domain

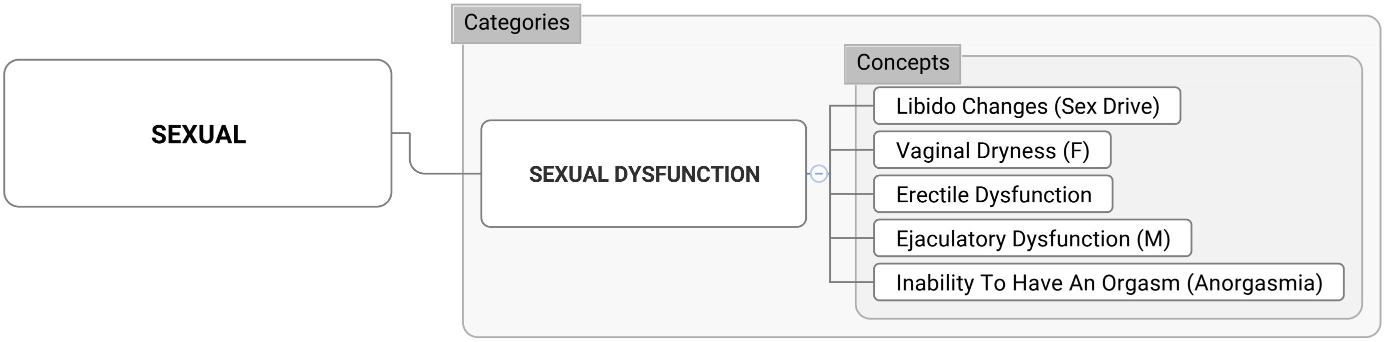

Supplementary Figure 11. Autonomic Nervous System Domain

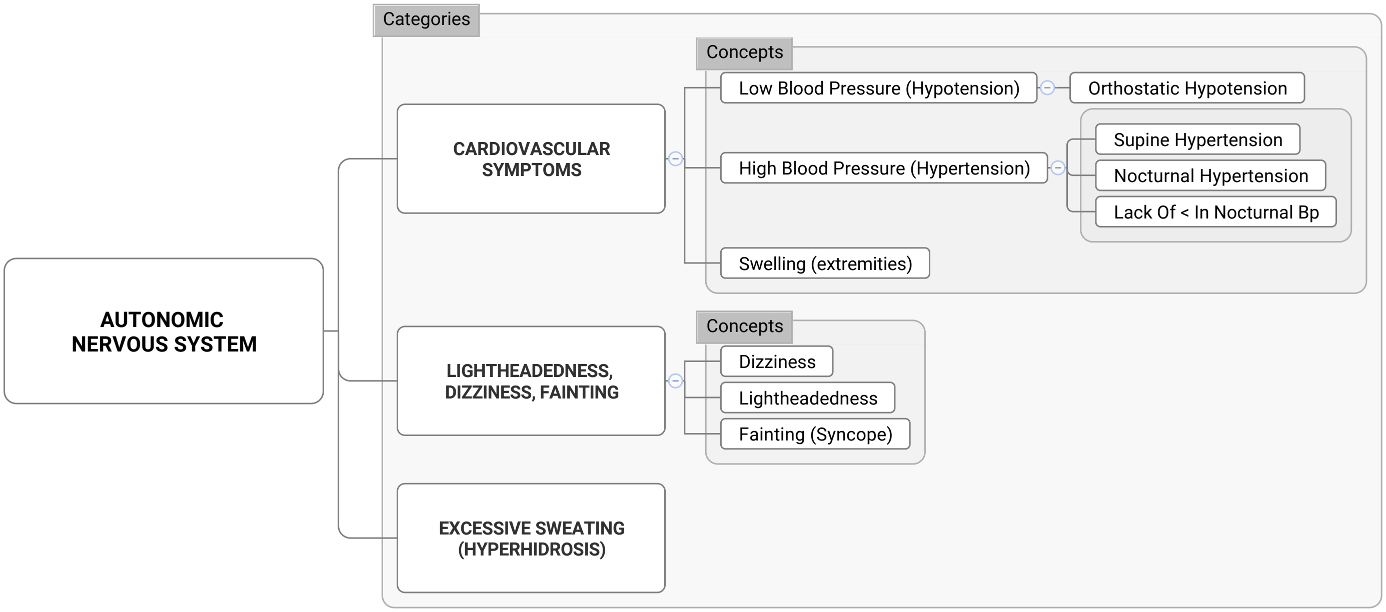

Supplementary Figure 12. Physical Functioning Domain

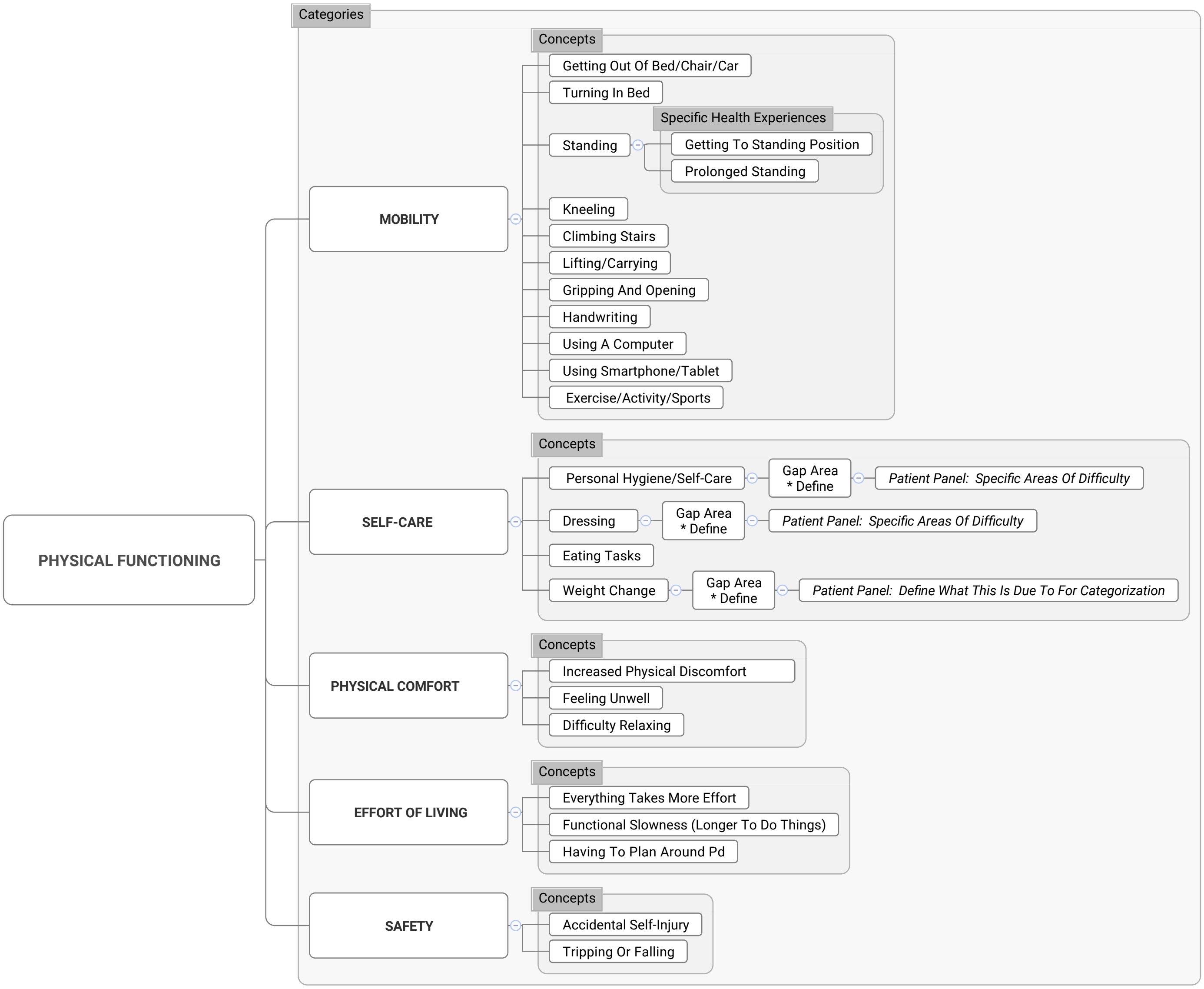

Supplementary Figure 13. Pyschosocial Functioning Domain

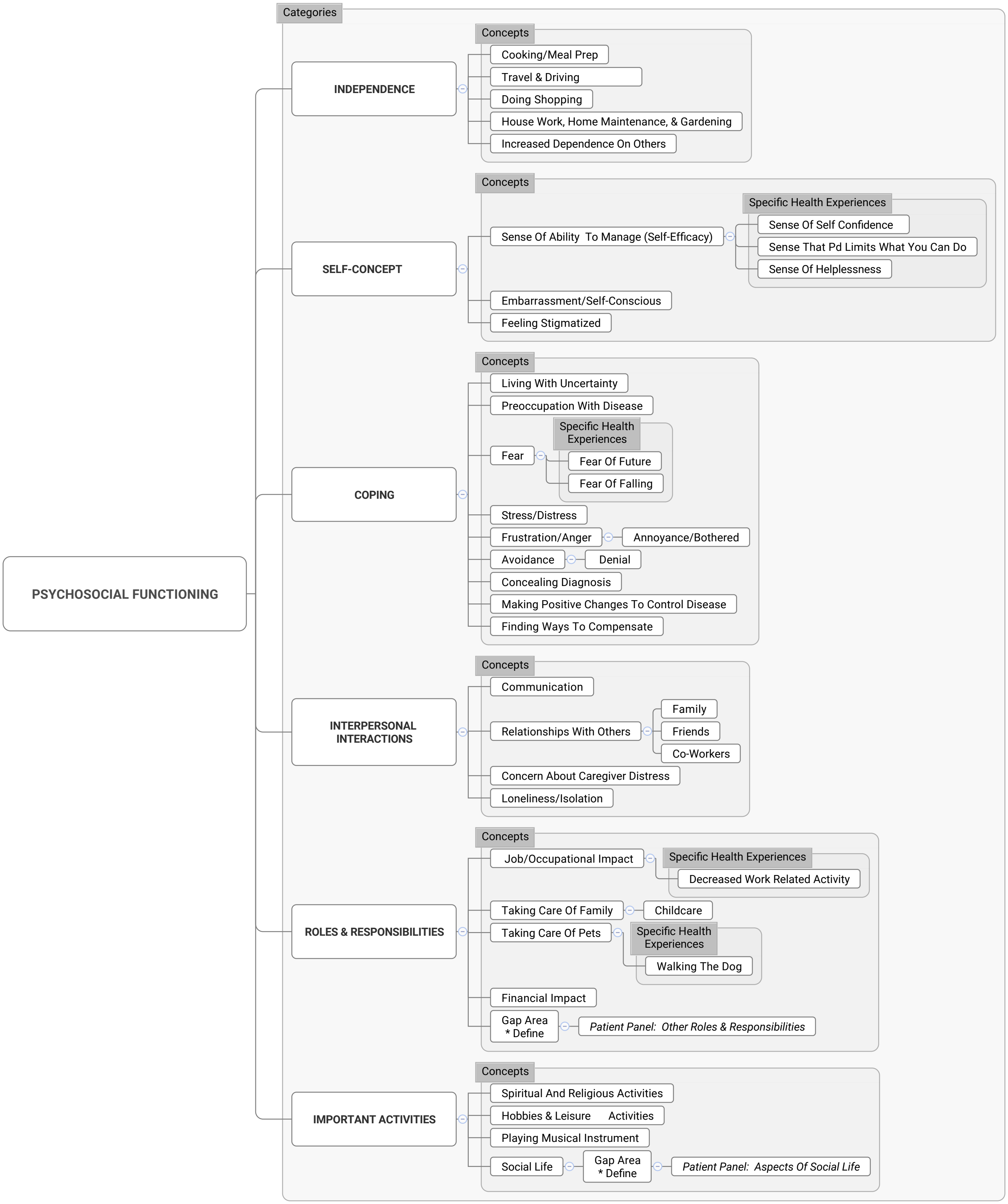

Supplementary Figure 14. Full conceptual model

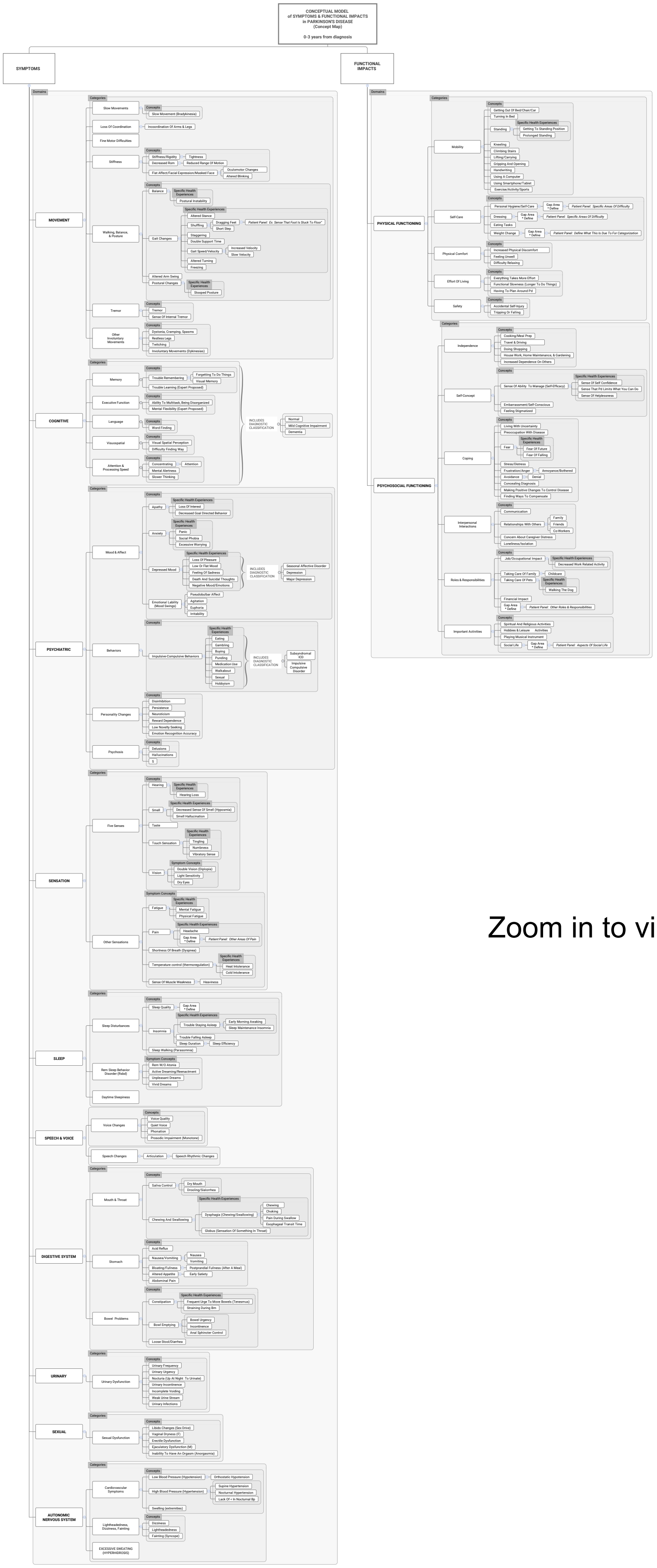

Zoom in to view details

Supplementary Table 1. Definitions of Concepts - Symptoms

Symptom term derived from literature review	Suggested modifications for use in future research	Proposed Terminology for use as an Outcome (neutral)	Proposed Definition	Definition Source	Excludes	Relevant Clinical Terminology	Patient Friendly Definition	Comments
MOVEMENT DOMAIN								
Slowed movements		Movement speed	Slowness of the limbs or generalized whole body movements including slowness performing activities	Common language and expert review	cognitive slowing	Hypokinesia, akinesia, bradykinesia	Slow Movements	
Altered fine motor dexterity		Fine motor dexterity	Impaired ability to do tasks requiring fine movement of the hand such as dressing, eating, writing, typing, etc.	Common language and expert review		Micrographia	Reduced fine Motor Control; Hand dexterity	
Loss of coordination		Coordination	Impaired coordination of extremities or trunk, may including missing target when attempting to reach for objects, bumping into things	Common language and expert review	fine motor; hand dexterity		Clumsiness, trouble with general coordination of movements	
Stiffness			Increased resistance with motion, may also be reported as stiffness or tightness	Common language and expert review	spasms, cramping, bending, twisting, dystonia	Rigidity, May included cogwheeling	Stiffness	
Decreased range of motion (ROM)		Range of Motion	Reduced ability to fully extend or flex body/extremities	Common language and expert review	stiffness, rigidity		Inability to fully extend or flex body parts	
Decreased facial expression		Facial expression	Flat affect, expressionless facial movements	Common language and expert review		Masked facies, Hypomimia	Reduced facial expressions	
Impaired eye movements		Eye movements	Altered eye muscle movements that include decreased blink rate or restriction of ocular movements	Common language and expert review		Oculomotor dysfunction	Changes in movement of eyes and eyelids, NOT including double vision	
Impaired balance		Balance	Difficulty maintaining balance while not being supported, which may or may not include requiring support to maintain balance, and may include a tendency to trip or fall	Common language and expert review		Retropulsion, Postural Instability	Impaired balance, trouble balancing	
Gait changes		Gait	Changes in quality of walking	Common language and expert review			Changes in walking	Categorical concept that may have diverse aspects and include specific experiences of shuffling, altered stride length, altered turning, double support time, etc.
Shuffling			Diminished foot clearance when walking resulting in dragging of the feet	Common language and expert review			Dragging feet when walking	
Altered stride length		Stride length	Abnormal shortened distance between steps	Common language and expert review		Festination	Shortened length of steps	
Altered stance		Stance	Abnormal ability to stand independently upright on two feet which may result in compensatory foot placement	Common language and expert review			Altered stance	
Altered turning		Turning	Interrupted or segmented turning, non-fluid changes in direction	Common language and expert review		En bloc turning	Non-fluid turns	
Altered double support time		Double support time	Amount of time spent while walking with two feet stabilized on the ground	Common language and expert review			Time spent with both feet on ground when walking	
Staggering			Unsteady gait with redirection to maintain balance resulting in deviation from walking in a straight line	Common language and expert review			Wavering, staggering	
Freezing			Failure to initiate or delay of initiation of movement, may also be the temporary arrest of ongoing movement	Common language and expert review	the 'fear of' freezing		Stuck when walking	
Altered gait velocity		Gait speed	Slowed gait with increase transit time or faster gait with decreased transit time	Common language and expert review		Festination	Change in walking speed	
Altered arm swing		Arm swing	Irregular or reduced arm swing during walking	Common language and expert review			Reduced arm swing	
Postural changes		Posture	Deviation of trunk; any change in posture resulting in person being not fully upright	Common language and expert review		Camptocormia, Pisa Syndrome	Stooped Posture, leaning	
Tremor			Oscillating, rhythmic involuntary movement of any body part	Common language and expert review	involuntary movements not specified as tremor or shaking, 'shakiness' related to an anxiety-related condition or internal sensation	Rest tremor, postural tremor, action tremor	Tremor	
Internal tremor			Invisible rhythmic sensation, may be of any body part, not visually observable, but felt	Common language and expert review	shakiness or jitteriness reported to be due to anxiety, restlessness		Inner sense of tremor	
Twitching			Brief, sudden, involuntary contraction of a group of muscle fibers	Common language and expert review		Myoclonus, fasiculations	Twitching	
Dystonia, Cramping & Spasms	Consider dividing as:<br>1. Dystonia<br>2. Cramping & Spasm		1. Abnormal posture of a body part due to involuntary sustained muscle contraction, includes leaning<br>2. Painful contractions of muscle(s)	Common language and expert review	Cramping in the context of abdominal pain or gastrointestinal discomfort	Dystonia	1. Sustained involuntary muscle contraction resulting in abnormal posture of a body part<br>2. Cramping, spasms	Patients may not be able to discriminate, and these are often reported compositely in the literature.
Dyskinesia			Involuntary writhing/dancing/swaying movement of the face, arms, legs or trunk; can be secondary to medication wearing off or peak dose effect	Common language and expert review	inner restlessness/akasthisia	Motor fluctuation, chorea, athetosis, Dyskinesia	Involuntary swaying movements	
Restless leg			Urge to move legs that is only temporarily relieved with movement, often more severe at night but may be present during the day	Common language and expert review	dyskinesias involving the legs		leg discomfort with urge to move	
COGNITIVE DOMAIN								
Impaired cognition		Cognition; cognitive abilities	Decline in cognitive abilities	Common language and expert review		Mild cognitive impairment	Changes in ability to think	
Dementia			Impairment of cognitive function leading to significant limitation in domains of living	Common language and expert review		Severe cognitive impairment	Changes in ability to think leading to difficulty doing tasks of daily life	
Impaired memory		Memory	Memory function, including ability/difficulty remembering information, orientation to time/date, and short and long term recall	Common language and expert review			Difficulty remembering information	
Forgetting to do things		Forgetfulness	An aspect of memory; Forgetting to follow up and complete desired tasks	Common language and expert review			Forgeting to do things; forgetfulness	

Symptom term derived from literature review	Suggested modifications for use in future research	Proposed Terminology for use as an Outcome (neutral)	Proposed Definition	Definition Source	Excludes	Relevant Clinical Terminology	Patient Friendly Definition	Comments
Impaired visual memory		Visual memory	Difficulties with visual recall may result in challenges navigating spaces that were once familiar	Common language and expert review			Difficulty remembering what things look like; difficulty visually recalling how to get places	
Impaired executive function		Executive function	Ability to engage in multi-level planning and decision-making to allow for successful completion of tasks	Common language and expert review		Frontal lobe function	Difficulty planning and making decisions	
Impaired ability to multitask		Multitasking	Ability to adapt and switch from one cognitive task to another or do more than one task at the same time	Common language and expert review				
Difficulty word finding		Word finding	Abilities for expressing oneself verbally or recalling intended words for conversation	Common language and expert review	difficulties understanding due to hearing impairment, dysarthria	Aphasia		
Impaired depth perception		Depth perception	Ability to judge distances or depth includes orienting onself in space and identifying spatial relationships amoung objects	Common language and expert review	freezing in doorways or thresholds	Visual spatial	Depth perception	
Difficulty finding way		Ability to find one's way	Becoming lost or disoriented with navigation	Common language and expert review				
Impaired attention		Attention	Ability to maintain focus on stimuli, tasks or conversation	Common language and expert review			Attention or trouble staying focused	
Impaired concentration		Concentration	Ability to maintain focus during task completion or questioning	Common language and expert review			Focus	
Slower thinking		Cognitive processing speed	Slowing of mental processing. Includes difficulty keeping up with conversations or slowness to respond	Common language and expert review			Slower thinking	
Impaired mental alertness		Mental alterness	Feeling mentally sharp and alert, or difficulty with cognitive processing that may include confused, muddled, or mixed up	Common language and expert review	Daytime sleepiness		Brain fog	
PSYCHIATRIC DOMAIN								
Apathy			Lack of desire, motivation, or interest to participate in hobbies, socialization, or activities of daily living	Common language and expert review			Loss of interest/motivation	
Anxious mood			Excessive worry or fear that persists often resulting in unease or avoidance behavior	Common language and expert review		Anxiety	Nervousness, feeling anxious, anxiety	
Social phobia			Excessive and irrational fear/anxiety when faced with social interaction	Common language and expert review		Specific phobia	Fear of social interaction	
Panic			Sudden uncontrollable and often irrational fear, intense worry that is often debilitating	Common language and expert review				
Depressed mood			Persistent saddness, helplessness, worthlessness, or empty mood	Common language and expert review		Depression	Feeling down or depressed	
Low or flat mood	Consider dividing as:<br>1. Low mood<br>2. Flat mood		1. Low mood that the individual does not qualify as depressed mood<br>2. Mood with little variation, lessened reactivity to emotion	Common language and expert review	Excludes feeling depressed	Blunted affect		Patients may not be able to discriminate, and these are often reported compositely in the literature.
Feeling of sadness			Feelings of sadness, may be associated with increased tearfulness	Common language and expert review	suicidal ideation	Depression	Feelings of being sad	
Loss of pleasure		Pleasure	Inability or diminished ability to feel pleasure or find enjoyment	Common language and expert review		Anhedonia	Lack of joy and pleasure	
Negative feelings & emotions			Persistent negative thoughts or feelings	Common language and expert review	excludes feeling depressed or sad or suicidal ideation	Pessimism	Negative thoughts, feeling more negative	
Suicidal ideation or thoughts of death			Thoughts of personal death either passively or with intended plan	Common language and expert review		Suicidality	Thoughts of death or suicide	
Pseudobulbar affect			Sudden uncontrollable strong emotional expression (ex. crying/laughing) at times seemingly unprovoked and not matching internal feelings	Common language and expert review		Emotional lability	Sudden laughing/crying that does not relate to your feelings	
Irritability			Easily frustrated or annoyed often over seemingly small matters	Common language and expert review			Easily frustrated or annoyed	
Euphoria			Feeling of extreme happiness or excitement	Common language and expert review			Intense happiness that may be excessive for the situation	
Agitation			Heightened restlessness/nervousness which may more easily lead to an angered response	Common language and expert review			Restless and on edge	
Impulsive behaviors			Difficulty controlling behaviors resulting in inability to resist temptation, often resulting in actions that are atypical for the person	Common language and expert review		Impulsivity	Doing things impulsively in response to urges that are difficult to control	
Medication use compulsion			Pattern of medication use of dysregulated over consumption and loss of control or urge towards increased intake	Common language and expert review		Dopamine dysregulation syndrome	Medication overuse due to an urge to take more than prescribed	
Buying compulsion			Excessive urge or tendency to shop or spend money often with adverse consequence	Common language and expert review		Oniomania	Excessive shopping urge	
Eating compulsion			Intense urge or tendency to over eat despite not being hungry	Common language and expert review		Hyperphagia	Excessive food cravings	
Sexual compulsion			Heightened focus on sexual urges or behaviors often with limited self-control	Common language and expert review		Hypersexuality	Excessive sexual urge	
Punding compulsion			Intense fascination of repetitive non-goal oriented mechanical tasks, may include assembling/disassembling and sorting	Common language and expert review	Obsessive Compulsive Disorder		Doing tasks repetitively without a reasonable goal (e.g. repetitive sorting)	
Gambling compulsion			Excessive urge or tendency to bet or gamble often with adverse consequence	Common language and expert review		Pathological gambling	Excessive urge to gamble	
Walkabout compulsion			Persisent urge to walk often aimlessly without intended destination	Common language and expert review			Excessive urge to wander about	
Hobbyism compulsion			Uncontrollable urge to carry out or pursue a hobby often prioritizing above other responsibilities	Common language and expert review			Excessive urge to do a hobby	

Symptom term derived from literature review	Suggested modifications for use in future research	Proposed Terminology for use as an Outcome (neutral)	Proposed Definition	Definition Source	Excludes	Relevant Clinical Terminology	Patient Friendly Definition	Comments
Psychosis			Condition in which thoughts, emotions, or perceptions are lost to reality and often with limited insight	Common language and expert review			Difficulty telling what is real and what is not real	
Delusions			False belief or distortion of reality that persists despite evidence to the contrary	Common language and expert review			mistaken beliefs about things that aren't real	
Hallucinations			Seeing, hearing, feeling, tasting, or smelling something that is not actually present, includes sensations of someone or something passing nearby in periphery	Common language and expert review	Misperception due to visual impairment		Seeing and hearing things that aren't real (e.g. visions or sounds)	
Smell hallucinations			Smelling something that is not present or perceived by others; Subset of hallucinations	Common language and expert review		Olfactory hallucination	Smelling things that are not there.	
Personality changes			Altered manner in how one thinks, acts, or feels that are distinctly uncharacteristic, may affect interactions with others	Common language and expert review			Personality changes	
Excessive persistence			Determination to complete tasks often with inflexibility or with fixated thought	Common language and expert review		Perseveration	Feeling compelled to keep doing something	Not enough information; present in one study.
Reward seeking behavior			Increased tendency to seek approval or respond to rewarding stimuli	Common language and expert review		Reward dependence	Reward seeking behavior	
Disinhibition			Inability to regulate behaviors or thoughts within bounds of socially acceptable behavior	Common language and expert review		Frontal lobe function	Inability to control behaviors in a way that is socially acceptable; Loss of filter	
Neuroticism			Increased distress or dissatifaction with oneself or external circumstance	Common language and expert review			Easily frazzled	
Impaired emotion recognition		Emotion recognition accuracy	Inability to successfully recognize and interpret others emotions	Common language and expert review			Trouble recognizing others feelings and emotions	
Low novelty seeking		Novelty seeking	Tendency to avoid the pursuit of new or exciting experiences and situations	Common language and expert review			Limited interest in trying new things	
SENSORY DOMAIN								
Double Vision			Seeing two of the same objects simultaneously	Common language and expert review		Diplopia	Double vision	
Light sensitivity			Discomfort when placed in bright environments	Common language and expert review		Photosensitivity	Visually sensitive to light; light sensitivity	
Dry eyes			Limited tear production which may result in eye irritation or discomfort	Common language and expert review		Xerophthalmia	Dry eyes	
Altered taste		Taste	Changes in quality of the taste of food	Common language and expert review		Gustation	Altered sense of taste	
Altered smell		Smell	Changes in quality of smell, may include food but also surrounding environment	Common language and expert review		Olfaction	Altered sense of smell	
Impaired hearing		Hearing	Changes in quality of hearing, may include distortions of sound or diminished ability to perceive/interpret sound	Common language and expert review	Does not include auditory hallucination	Auditory perception	Altered sense of hearing	
Impaired sensation		Sensation	Changes in quality of touch sensation, may include light touch, pressure, or sensory disturbance not otherwise specified below	Common language and expert review		Tactile perception	Altered touch sensation	
Impaired sensation in hands and feet			Subset of impaired sensation: Numbness or sensory disturbance most pronounced of distal arms/legs	Common language and expert review		Peripheral neuropathy	Numbness or tingling of hands/feet	
Impaired vibratory sense		Vibratory sense	Impaired ability to perceive vibratory stimuli	Common language and expert review			Loss of ability to feel vibrations (skin)	
Numbness			Diminished ability to perceive to tactile stimuli	Common language and expert review			Loss of sensation in skin	
Tingling			Sensory disturbance that may be compared to pins and needles or when an extremity 'falls asleep' following restricted blood flow	Common language and expert review			Tingling sensation in skin	
Impaired temperature sensation		Temperature sensation	Impaired ability to differentiate between hot and cold	Common language and expert review		Thermosensation	Altered ability to feel hot and cold	
Pain			Body pain or discomfort due to any cause including cramping, spasms, or neuropathy	Common language and expert review		Nociception	Pain, physical discomfort	
Headache			Head pain or discomfort due to any cause	Common language and expert review			Headache	
Fatigue - General		Energy level	Overall lack of energy not otherwise specified	Common language and expert review	sleepiness		lack of energy; feeling too tired to do things	
Fatigue - Mental		Mental energy	Sense of mental exhaustion not explained by drug effects or other psychiatric condition, includes feeling exhausted during intellectually challenging tasks or decreased capacity to sustain cognitively challenging activites	Common language and expert review	sleepiness		Feeling mentally exhausted	
Fatigue - Physical		Physical energy	Exhaustion or tiredness of the body	Common language and expert review	sleepiness		Feeling physically exhausted	
Shortness of breath			Difficult or labored breathing	Common language and expert review	Does not included proportionate exercise induced SOB	Dyspnea	Feeling abnormally short of breath	
Muscle weakness		Muscle strength	Diminished ability to produce maximal motor strength	Common language and expert review			Loss of strength; feeling weak	
Heaviness			Maximal strength is preserved but takes more effort or energy to exert force or make movement	Common language and expert review			Sensation of heavy limbs such that physical tasks require more effort	Infrequently reported and may confound with muscle weakness or fatigue
Impaired body temperature control		Body temperature control	Feeling (perception) of being either excessively hot or cold, may include generalized sensation or be specific to certain parts of the body	Common language and expert review	chills or sensory disturbances secondary to fever/infection	Thermoregulation	Difficulty managing body temperature; Feeling excessively hot or cold	
Heat intolerance			Significant discomfort when placed in warm temperature environments	Common language and expert review			Feeling abnormally hot	

Symptom term derived from literature review	Suggested modifications for use in future research	Proposed Terminology for use as an Outcome (neutral)	Proposed Definition	Definition Source	Excludes	Relevant Clinical Terminology	Patient Friendly Definition	Comments
Cold intolerance			Significant discomfort when placed in cold temperature environments	Common language and expert review			Feeling abnormally cold	
SLEEP DOMAIN								
Altered Sleep		Sleep	Changes in patterns or behaviors when trying to sleep	Common language and expert review			Sleep changes	
Sleep disturbances (general term)			Undesired interruptions or disruptions of sleep	Common language and expert review			Disturbed or interrupted sleep	
Reduced sleep quality		Sleep quality	Perception of not having a good night of sleep	Common language and expert review			Quality of sleep; sleeping well	
Sleep onset insomnia			Difficulty falling asleep at the beginning of the sleep period	Common language and expert review	difficulties initiating sleep due to restless legs		Difficulty falling asleep	
Sleep maintenance insomnia			Difficulty staying asleep; Awakening during the night with difficulty falling asleep again	Common language and expert review	brief awakenings (ex. due to trips to the bathroom) without difficulty falling back asleep		Difficulty staying asleep; interrupted sleep; broken sleep	
Early morning awakening			Waking up too early with difficulty falling back to sleep	Common language and expert review	Does not included planned early morning wakening, such as for occupation		Waking up too early	
Altered sleep duration & efficiency		Sleep duration & efficiency	Changes in how long one sleeps and overall time one maintains sleep, how restful one feels upon awakening	Common language and expert review			How long you stay asleep	
Sleep talking/walking			Vocalizations, talking, or walking during sleep without awareness of behavior	Common language and expert review	dream enactment, sleep paralysis			
REM Sleep Behavior Disorder (RBD)		REM sleep	Acting out dreams, movements or vocalizations during sleep; may only be aware due to partner report; may at times cause patient to awaken	Common language and expert review	moving in sleep without further specification	REM Sleep Behavior Disorder	Acting out dreams	
Vivid dreams			Any report of vivid or detailed dreams, dreams that seem real or can recall in great detail upon awakening	Common language and expert review			Intense memorable dreams	
Unpleasant dreams			Intense dreams, nightmares	Common language and expert review				
Daytime sleepiness			Urge or need to sleep in situations when is sleep not desired or appropriate, may lead to intentional or unintentional sleep during the day	Common language and expert review	tiredness or fatigue without needing to sleep/nap	Hypersomnia		
SPEECH & VOICE DOMAIN								
Impaired articulation		Pronunciation	Ability to form clear distinct sounds, clarity of speech production	Common language and expert review		Dysarthria	Clarity of speech	Patient confound articulation with being articulate; Pronunciation is preferred term.
Impaired phonation			Ability to produce sound from the vocal tract	Common language and expert review			Ability to produce sound	
Monotone voice			Lack of vocal fluctuation in tone or rhythm	Common language and expert review		Monotone; Prosodic impairment	Intonation/rhythm of speech	
Hypophonia			Low speech volume/amplitude, difficulty projecting voice	Common language and expert review			Quiet voice	
Altered voice quality		Voice quality	Changes in character or quality of voice not otherwise specified (e.g., raspiness)	Common language and expert review				
Impaired verbal fluency		Verbal fluency	Impaired ability to produce desired vocabulary when communicating	Common language and expert review			Ease of word production	
DIGESTIVE SYSTEM DOMAIN								
Drooling			Difficulty managing saliva with overflow out of the mouth	Common language and expert review		Sialorrhea		
Dry mouth			Sensation of inadequate saliva production	Common language and expert review		Xerostomia		
Globus sensation			Sensation of having something stuck in one's throat	Common language and expert review		Globus pharyngis		
Impaired swallowing		Swallowing	Swallowing impairment that may result in coughing/choking when trying to ingest food and drink	Common language and expert review		Aspiration; Dysphagia	Swallowing difficulties	
Altered chewing		Chewing	Changes in ability to masticate food to allow for succesful feeding	Common language and expert review		Mastication		
Pain during swallow			Pain or discomfort of any part of the oropharynx or digestive tract provoked by swallowing	Common language and expert review		Odynophagia		
Altered esophageal transit time		Esophageal transit time	Changes in the amount of time it takes for food/drink to traverse down the esophagus into the stomach	Common language and expert review			Time it takes for food to enter stomach	
Acid reflux			Upper abdominal/chest discomfort caused by stomach acid irritating the lower part of the esophagus often worsened following meals or when lying down	Common language and expert review		GERD	Heartburn	
Early fullness			Feeling full after only a small amount of food intake	Common language and expert review		Early saiety	Feeling full quickly	
Abdominal pain			Pain or discomfort attributed to the GI tract	Common language and expert review			Stomach or belly pain or ache	
Nausea & Vomiting			Sensation of stomach discomfort with concern that one may vomit or progression to vomiting	Common language and expert review		Emesis		
Bloating/Fullness			Feeling of abdominal fullness/tightness often with associated distention	Common language and expert review		Gastroparesis	Belly feeling full or large	
Appetite changes		Appetite	Changes in desire to eat, may result in either weight loss or weight gain	Common language and expert review				
Constipation			Difficulty with passage of stool, often accompanied by hardened feces	Common language and expert review		Fecal impaction		

Symptom term derived from literature review	Suggested modifications for use in future research	Proposed Terminology for use as an Outcome (neutral)	Proposed Definition	Definition Source	Excludes	Relevant Clinical Terminology	Patient Friendly Definition	Comments
Tenesmus			Urge to pass stool accompanied by pain/cramping and often unsuccessful with limited defecation	Common language and expert review	pain passing hard stool		Feeling the need to pass stool without being able to; may be painful	
Straining			Increased required force or effort exerted during bowel movements	Common language and expert review				
Bowel urgency			The need to suddenly relieve bowels to prevent accidental soiling	Common language and expert review				
Bowel incontinence			Loss of ability to hold bowels often without forewarning leading to involuntary soiling	Common language and expert review		Encopresis	Losing control of stool leading to soiling	
Impaired anal sphincter control			Impaired ability to willingly tighten or loosen one's anal sphincter to allow for coordination of bowel movements	Common language and expert review				
Diarrhea			Bowel movements of loose or watery consistency	Common language and expert review			Loose stool	
URINARY DOMAIN								
Urinary Frequency			The need to pass urine more frequently than typical or desired	Common language and expert review			Urge to urinate frequently	
Urinary Urgency			The need to suddenly relieve urine often without forewarning	Common language and expert review			Urge to urinate suddenly	
Nocturia			Awakening at night to urinate, may occur multiple times per night and disrupt sleep	Common language and expert review			Need to urinate at night	
Incomplete voiding			Inability to fully void urine from bladder	Common language and expert review			Incomplete bladder emptying	
Urinary Incontinence			Loss of ability to hold urine or regulate desired timing of urination	Common language and expert review			Loss of bladder control	
Urinary Infections			Associated infections of the urinary system	Common language and expert review		Urinary tract infection		
Weak urine stream			Diminished outflow velocity when urinating	Common language and expert review		Urinary hesitancy		
SEXUAL DOMAIN								
Sexual dysfunction		Sexual function	Difficulties with sexual response, desire, or performance not otherwise specified	Common language and expert review				
Trouble achieving orgasm		Ability to achieve orgasm	Absent, delayed, or diminished orgasm following adequate sexual stimulation	Common language and expert review		Anorgasmia	Difficulty achieving orgasm	
Ejaculatory dysfunction		Ability to ejaculate	Absent, delayed, diminished, or premature ability to ejaculate during sexual intercourse	Common language and expert review			Difficulty ejaculating or early ejaculation	
Erectile dysfunction		Ability to achieve erection	Difficulty achieving or maintaining an erection during sex	Common language and expert review		Impotence		
Impaired libido		Libido; sexual desire	Lack of interest in sexual activity	Common language and expert review			Low sex drive	
Vaginal dryness			Dryness of the vagina, often may lead to irritation or pain with intercourse	Common language and expert review		Vaginal atrophy		
AUTONOMIC NERVOUS SYSTEM DOMAIN								
Orthostatic hypotension		Blood pressure	Low blood pressure provoked with standing from sitting or lying down, may result in lightheadedness or episodes of passing out	Common language and expert review			Low blood pressure when standing	
Hypotension		Blood pressure	Low blood pressure	Common language and expert review			Low blood pressure	
Hypertension		Blood pressure	Elevated blood pressure	Common language and expert review			high blood pressure	
Supine hypertension		Blood pressure	Elevated blood pressures noted particularly when lying down	Common language and expert review			High blood pressure when lying down	
Nocturnal hypertension		Blood pressure	Elevated blood pressures noted overnight when attempting to sleep	Common language and expert review			High blood pressure overnight	
Swelling of extremities			Swelling due to retention of fluid commonly involving distal legs but may also involve arms	Common language and expert review		Peripheral edema	Swelling of arms or legs	
Dizziness			Altered sense of self perceived place, may be described as either the room or the person spinning which may cause one to lose balance	Common language and expert review	lightheadedness		Dizzy or spinning sensation	May be indistinguishable from lightheadedness from patient perspective
Lightheadedness			Altered sensation that one may faint, often positionally provoked and may improve when seated from standing or lying flat	Common language and expert review	dizziness		Faintness	May be indistinguishable from dizziness from patient perspective
Syncope			Loss of consciousness, often brief with quick recovery	Common language and expert review			Passing out	
Hyperhidrosis			Excessive sweating that may involve the entire body or specific areas such as under arms, soles, face, etc.	Common language and expert review	sweating secondary to panic, distress, or anxiety related events		Excessive sweating	

Supplementary Table 2. Definitions of Concepts - Impact

Impact Terminology Derived from Literature Review	Suggested modifications for use in future research	Proposed Alternate Terminology for use as an Outcome (neutral)	Proposed Definition	Definition Source	Excludes	Relevant Clinical Terminology	Patient Friendly Definitior	Comments
PHYSICAL FUNCTIONING DOMAIN								
MOBILITY								
Mobility			Purposeful physical movement used to navigate one's enviroment including gross simple movements, fine complex movements, and muscle coordination	Adopted from Giddens, 2017		Ambulation, dexterity	Movement; walking	
Getting out of bed/chair/car			Ability to get up from a seated position and from different heights or surfaces	Common language and expert review				Potential distinctions between these concepts may or may not be meaningful and there may be room to blend with aspects of getting to standing position.
Turning in bed			Ability to roll over and change positions in bed	Common language and expert review				
Standing	Divide as:<br>1. Standing up<br>2. Standing for a period of time		1. Ability to get to standing position; and 2. Ability to maintain erect posture for a period of time	Common language and expert review				Subconcept 1 could possibly be combined with "getting out of bed/chair" as the concept "Getting up from sitting or lying down."
Kneeling/bending	Divide as:<br>1. Kneeling<br>2. Bending		1. Use of musculoskeletal system to assume a position where the body is supported by the knees; and 2. Use of the musculoskeletal system to incline the body downward from a standing position	Common language and expert review				Combines two concepts that are not clearly related.
Going up or down stairs			1. Ability to independently walk upwards on a set of stairs or steps; and 2. Ability to independently walk downwards on a set of stairs or steps	Common language and expert review				Climbing stairs is sometimes used to assess stamina, rather than musculoskeletal function, and use is not defined in early PD literature
Lifting/holding/carrying	Divide as:<br>1. Lifting<br>2. Holding<br>3. Carrying		1. Ability to use musculoskeletal system to pick up and move an object to a different position; 2. Ability to keep an object in one's hand or arms; and 3. Ability to walk with an object while holding it	Common language and expert review				Distinguishing between the three elements may be appropriate to assessing upper limb functioning in some contexts of use when precise information about specific aspects of functioning is needed.
Gripping and opening	Divide as:<br>1. Gripping<br>2. Ability to open something		1. Ability to grasp an item and apply tension, maximum strength generated by forearm muscles; and 2. Functional ablity and muscular strength required to twist the hand or forearm and pull or lift the object to open it	Common language and expert review				Distinction may be appropriate to assessing the degree to which strength and coordination contribute to changes in functioning.
Handwriting		Ability to write clearly by hand with ease	Use of a writing instrument, such as a pen, pencil, or marker and fine motor skills of the hand	Common language and expert review			Penmanship	
Using a computer		Ability to use a device keyboard, buttons, and/or mouse	Ability to operate a computer	Common language and expert review	Limitations secondary to lack of technological knowledge			Predominantly due to physical difficulty but may also have a cognitive component.
Using smartphone/tablet		Ability to tap accurately on a device touchpad or screen	Ability to use a handheld electronic device	Common language and expert review	Limitations secondary to lack of technological knowledge			Predominantly due to physical difficulty but may also have a cognitive component.
Exercise/activity/sports			Ability to engage in physical activity to improve health and fitness, or for social and leisure purposes	Common language and expert review			Ability to be physically active, including running; hiking; biking	Distinguishing between exercise/sports as a physical vs. social impact may be appropriate in some contexts of use, and may require further specification. For example, ability to exercise may be used to assess stamina or level of fatigue, but may require specification that exercise is vigorous.
**PHYSICAL COMFORT**								
Increased physical discomfort		Physical comfort	An unpleasant sensory experience; inability to achieve physical comfort, such as feeling relaxed, at ease, and able to hold a normal physical position without distress	Adopted from Giddens, 2017	Excludes pain (symptom)	Discomfort	Discomfort	
Feeling unwell		Feeling of well-being	General malaise; sensation of not feeling well or not at baseline	Common language and expert review	Emotional distress			
Difficulty relaxing		Ability to relax	State of increased tension with inability to fully resolve; Ability to reduce muscle tension, slow breathing, and calm thought process	Common language and expert review				
**EFFORT OF LIVING**								
Effort of daily living			Energy or effort (mental or physical) expended to perform usual daily living activities	Common language and expert review				
Functional slowness			Prolonged time required to complete tasks, often due to slow effortful movements and decreased motor control	Common language and expert review		Bradykinesia	Takes longer to do things	
Increased effort to do things			Increased energy expended to perform all tasks (overlaps with categorical concept)	Common language and expert review			Feeling drained	
Having to plan around PD			Having to coordinate schedule and alter plans to accommodate for impact of PD	Common language and expert review		Motor fluctuations, ON/OFF time		
**SELF-CARE**								
Dressing			Ability to put on or take off clothing without help	Common language and expert review				
Personal hygiene			Tasks to maintain personal cleanliness (showering, brushing teeth, grooming, etc.) without help	Common language and expert review				Distinguishing between different personal hygiene activities may be appropriate in some contexts of use or provide more precise information about change in functioning.
Eating tasks		Ability to eat and drink without help	Ability to feed oneself or independently consume food/beverages without help	Common language and expert review		Nutrition		
**SAFETY**								
Unintentional self-injury			Injuries to oneself that are not inflicted purposely, commonly resulting from cognitive or motor impairments	Common language and expert review	Self-harm			
Tripping and falling	Divide as:<br>1. Tripping<br>2. Falling		1. Difficulties with foot clearance, motor control, or imbalance which may result in near or actual falls; and 2. Inability to maintain or regain balance in standing position; falling as a result of tripping, bending over	Common language and expert review	Falling out of bed	Gait imbalance, freezing, falls	Unsteadiness	Additional evidence may be needed to understand what the concept really is and to what extent tripping and falling are separate. Tripping can occur without falling and falling can occur without tripping. However, "tripping-and-then-falling-because-of-tripping" could be a appropriate concept for early PD.
Weight change			Unplanned weight loss or gain that may or may not be secondary to changes in appetite	Common language and expert review				
PSYCHOSOCIAL FUNCTIONING DOMAIN								
**INDEPENDENCE**								
Independence			Ability to be self-reliant and complete necessary and desirable tasks and activities without undue assistance from others	Common language and expert review			Being self-reliant	
Cooking/Meal prep			Act of preparing a meal for consumption	Common language and expert review				
Shopping			Act of purchasing goods from a store or online	Common language and expert review				

Impact Terminology Derived from Literature Review	Suggested modifications for use in future research	Proposed Alternate Terminology for use as an Outcome (neutral)	Proposed Definition	Definition Source	Excludes	Relevant Clinical Terminology	Patient Friendly Definitior	Comments
House work/home maintenance/garden		Ability to carry out one's home care tasks and responsibilities	Ability to engage in activities required for maintenance of a home (vacuuming, washing dishes, laundry, lawn care, etc.)	Common language and expert review				The specific tasks and the associated levels of functioning and/or stamina required to complete them can vary greatly depending on living circumstances, culture, and other factors. Depending on assessment aims, it may or may not be feasible or useful to articulate specific tasks as examples or to distinguish between types of tasks.
Travel/Driving	Divide as:<br>1. Traveling<br>2. Driving	Ability to get around by car or other means of transportation	1. Act of going to different places by using a means of transportation, including being a passenger in a car; and 2. Ability to operate a motor vehicle	Common language and expert review				Distinguishing between driving and other means of travel may not be appropriate to assessing independence. For example, many people do not have a car and/or do not need to drive, which affects the concept's usefulness in assessing independence in a varied population of people with PD.
**SELF-CONCEPT**								
Altered self-concept		Sense of identity	Change in beliefs/perception about oneself, feeling that one's identity or essential self has changed	Common language and expert review			Change in self-image	
Embarrassment/self-conscious	Divide as:<br>1. Embarassment<br>2. Self-conscious		1. Uncomfortable feeling generated from perceiving that one's condition draws attention from others or results in unwelcome or taboo occurances; and 2. A state of shame or worry about one's condition or symptoms	Common language and expert review				
Feeling stigmatized		Percieved stigma	Self perception of being negatively viewed, being treated unfairly	Common language and expert review				
**SELF-EFFICACY**								
Decreased self-confidence		Self-confidence	Diminished pride in oneself or belief in ability to be successful	Common language and expert review				
Sense of helplessness		Feeling capable	Feeling or perception that one's actions have limited ability to change or affect future outcomes	Common language and expert review				
Sense that PD limits what you can do		Feeling unrestricted	Perception that one's abilities are limited by symptoms	Common language and expert review				
**COPING**								
Altered coping		Coping	Decreased ability to manage stress or adverse conditions	Common language and expert review				
Living with uncertainty			Acknowledgement and awareness of the unpredictable and potentially undesirable nature of disease outcomes that may or may not cause anticipatory distress	Common language and expert review				
Preoccupation with disease			Intrusive thoughts or worry about the impact of disease burden/progression	Common language and expert review				
Increased fear		Fear	Unpleasant strong emotion and increased autonomic activity caused by anticipation or awareness of danger	Adopted from Merriam-Webster Dictonary, 2024				
Fear of falling			Anticipation and concern about the act of falling and its consequences	Common language and expert review				
Fear of future			Anticipation and dread about future or upcoming events or outcomes	Common language and expert review				
Avoidance			Behaviors that limit social interaction or pursuit of activities	Common language and expert review				
Denial			Refusal to accept diagnosis or reality of one's condition	Common language and expert review				
Concealing diagnosis			Choosing not to disclose one's diagnosis	Common language and expert review				
Annoyance/bothered	Divide as:<br>1. Annoyance<br>2. Bothered		Emotional state of feeling annoyed, irritated, or bothered about something	Common language and expert review				Further evidence may be needed to determine whether distinguishing between these two concepts is feasible or useful.
Frustration/Anger	Divide as:<br>1. Frustration<br>2. Anger		Emotional state of displeasure or animosity towards something	Common language and expert review				Further evidence may be needed to determine whether distinguishing between these two concepts is feasible or useful.
Stress, distress, overwhelmed			Percieved emotional feeling and hormonal reaction to a life event such as adversity, hardship, or illness where resources and abilites for management are available, exceeded, or exhausted	Adopted from Giddens, 2017				Further evidence may be needed to determine whether distinguishing between these three concepts is feasible or useful.
Finding ways to compensate		Ability to cope	Actions that compensate for the impact of one's symptoms or diagnosis	Common language and expert review				
Making positive changes to take control of disease		Adaptation to and management of disease	Adaptive behaviors to promote self empowerment and address uncertainty	Common language and expert review		Empowerment		
**INTERPERSONAL INTERACTIONS**								
Interpersonal interactions (all)		Ability to engage in desired level of social activity	Social involvement or engagement with others	Common language and expert review				
Relationships with others (friends, family, colleagues)		Satisfaction with family and social life	Perception of major roles and responsibilites in current life situation	Common language and expert review				
Perceptions and reactions of other people			Views and feelings about how other people perceive and react to one's symptoms, impairments, and needs or requirements	Common language and expert review				Term may benefit by clarification and may be related to feeling stigmatized
Communication			Ability to express thoughts verbally or by writing or typing, and to understand spoken or written language	Common language and expert review		Expressive language, receptive language		
Loneliness/isolation	Divide as:<br>1. Loneliness<br>2. Isolation		1. Feeling alone; emotional state that can occur in the presence of others as well as by oneself; and 2. Limited ability to go out and engage with others; keeping oneself apart from others	Common language and expert review				
**ROLES AND RESPONSIBILITIES**								
Taking care of family			Ability to provide for family, may include physical/emotional means	Common language and expert review				
Taking care of pets			Ability to provide for pets, may include feeding, elimination, exercise, veterinary care	Common language and expert review			Pet care	
Impact on job/career/profession			Influence symptoms/disease has on career aspirations or ability to achieve desired professional goals, as well as ability to attend work and complete work-related tasks accurately and on time	Common language and expert review				
Financial impact/paying bills			Economic strain or effect on affording costs of living	Common language and expert review			Worrying about money or finances	
IMPORTANT ACTIVITIES								
Pleasurable activities			Ability to engage in activities that bring enjoyment, may include recreational activities or tasks that provide greater sense of fufillment	Common language and expert review				
Loss of things you enjoy			Sense of loss due to inability to engage in desired enjoyable activities	Common language and expert review		Apathy		

Impact Terminology Derived from Literature Review	Suggested modifications for use in future research	Proposed Alternate Terminology for use as an Outcome (neutral)	Proposed Definition	Definition Source	Excludes	Relevant Clinical Terminology	Patient Friendly Definition	Comments
Social life			Social interactions with others spent doing enjoyable activities	Common language and expert review		Socialization		
Playing musical instrument			Ability to position oneself to hold or operate a musical instrument	Common language and expert review				
Spiritual and Religious activities			Ability to participate in an organized or independent spiritual or religious life	Common language and expert review				
Hobbies/leisure activities			Ability to engage in desired hobby or leisure activities	Common language and expert review				
CONTEXTUAL FACTORS								
Symmetrical symptom onset			Motor deficits that affect both sides of the body at beginning of disease or perceived awareness of symptoms	Common language and expert review				
Most dominant/obvious issues			Most prevalent or dominant symptom(s) that causes the greatest impact on daily life, health, and wellbeing	Common language and expert review				
Frequency and severity of symptoms			Frequency refers to the number of times a symptom occurs and severity refers to how severe a symptoms is when present	Common language and expert review				
Medication dose failure/delayed on			Limited efficacy following medication dosing either due to delay or limit in peak benefit	Common language and expert review				
Nocturnal motor symptoms			Motor symptoms that occur primarily at night often while trying to sleep	Common language and expert review				
Motor fluctuations			Changes in motor performance often in context of varied drug absorption/availability of dopamine	Common language and expert review		dyskinesias		
Energy intake			Total daily consumption of nutritional caloric intake	Common language and expert review		Nutrition	Eating	
"Off" periods			Moments of worsened motor symptoms resulting in functional limitation	Common language and expert review		Wearing off syndrome		
Medication side effects			Unintended adverse effect occuring at a normal dose	Common language and expert review		Pharmacological adverse effect		

References:

Giddens, J.F. (2017). *Concepts for nursing practice* (2nd Ed.). Elsevier

Merriam-Webster (2024). https://www.merriam-webster.com/dictionary/

Supplementary Table 3. Multi-data base search strategy

Database	Search terms	Date	Hits	Duplicates	Unique
	**SEARCH STRATEGY 1: Symptoms & Impacts in early PD**				
Web of Science	1. "early parkinson*" (Title) OR "early parkinson*" (Abstract)<br>2. "early PD*" (Title) OR "early PD*" (Abstract)<br>3. #2 OR #1<br>4. ((AB=(symptom*)) OR AB=(impact*)) AND #3<br>***Filtered***: within 10 years (2013—2023)	4/4/2023	595	-	595
PubMed	(("early PD"[Title/Abstract]) OR ("early Parkinson*"[Title/Abstract])) AND ((symptom*[Title/Abstract]) OR (impact*[Title/Abstract]))<br>***Filtered***: within 10 years (2013—2023)	4/4/2023	551	497	54
MEDLINE/OVID	1. ("early parkinson*" or "early parkinson*").ab.<br>2. ("early PD" or "early PD").ab.<br>3. ("early parkinson*" or "early parkinson*").ti.<br>4. ("early PD" or "early PD").ti.<br>5. (symptom* or symptom*).ti.<br>6. (impact* or impact*).ti.<br>7. (symptom* or symptom*).ab.<br>8. (impact* or impact*).ab.<br>9. 7 or 8<br>10. 5 or 6<br>11. 3 or 4<br>12. 1 or 2<br>13. 11 or 12<br>14. 9 or 10<br>15. 13 and 14<br>***Filtered***: within 10 years (2013—2023)	4/4/2023	546	540	6
EBSCO/CINHAL	1. TI "early parkinson*" OR AB "early parkinson*"<br>2. TI "early PD" OR AB "early PD"<br>3. 1 OR 2<br>4. TI impact* OR AB impact*<br>5. TI symptom* OR AB symptom*<br>6. 4 OR 5<br>7. 3 AND 6<br>8. (PY>2012) AND 7	4/4/2023	209	209	0
	**SEARCH STRATEGY 2 – Qualitative in early PD**				
Web of Science	((((((AB=(qualitative)) OR AB=(conceptual model)) OR AB=(conceptual framework)) OR AB=(lived experience)) OR AB=(illness experience)) OR AB=(phenomena)) OR AB=(phenomenon)) AND **((AB=("early PD")) OR AB=("early Parkinson*"))**	5/4/2023	48	10	38<br>(*4 relevant*)
PubMed	((((((("conceptual model"[Title/Abstract]) OR ("conceptual framework"[Title/Abstract])) OR (phenomena[Title/Abstract])) OR ("lived-experience"[Title/Abstract])) OR ("lived experience"[Title/Abstract])) OR ("illness experience"[Title/Abstract])) OR ("illness-experience"[Title/Abstract])) OR (qualitative[Title/Abstract])) AND (("early PD"[Title/Abstract]) OR ("early Parkinson*"[Title/Abstract]))	5/4/2023	26	16	10<br>(*2 relevant*)
MEDLINE/OVID	((ab: early w PD) or (ab: early w Parkinson*)) and (ab: qualitative or ab: phenomena) or ((ab: conceptual and ab: model)) or (((ab: lived and ab: experience)) or ((ab: illness and ab: experience)) or ((ab: conceptual and ab: framework)))	5/4/2023	23	23	0
EBSCO/CINHAL	(AB "early PD" OR AB "early Parkinson") AND (AB qualitative OR AB "conceptual model" OR AB "conceptual framework" OR AB phenomena OR AB "lived experience" OR AB "illness experience")	5/4/2023	6	6	0
	**SEARCH STRATEGY 3**				
Reference lists	Sources identified from reference lists in systematic and scoping reviews on symptoms or impacts of early PD.				
	**SEARCH STRATEGY 4**				
Expert review	Ahead of press and prepublication.<br>Seminal works older than 10 years.				2
	**TOTALS**				
					**705**

Supplementary References and Audit trail of sources included in the systematic review

Included in Full Review for concept identification (all "early" PD – study defined) N=88

Tier 1 (N=8): [4, 10-13, 15-17]
Tier 2 (N=27) and 3 (N=53) : [18-97] (Excluded T2/T3= [126-189])

Pooled Data from Same Sample studies (Full review for concept identification)

Tier 1: Parkinson's UK survey data [4, 16]
Tier 2:

- Chinese PSG [81, 94]
- ICICLE-PD [33, 34, 60]
- Norwegian Parkwest [59, 62, 87]
- OPDC [23, 24, 85]
- PPMI [52, 66, 79]

Tier 3

- Norwegian Parkwest [46, 58, 86, 89]
- ICICLE-PD [49, 57]
- PALS [29, 39, 43]
- PPMI [20, 21, 32, 38, 45, 48, 76, 78]
- Sichuan University [63, 64]

Removed from final model due to PD time since PD diagnosis >3 years (model constraints) N=32

- Tier 1: [17]
- Tier 2: [23, 59, 66, 72, 74, 81, 85, 90, 91, 94]
- Tier 3: [20, 24-26, 28, 37, 40-42, 44, 47, 51, 53, 56, 61, 63-65, 71, 75, 80, 89, 93]

Included in final model of <3years from diagnosis for final frequencies: Tier 1,2,3 - N=56

- Tiers 1-3: [4, 10-13, 15, 16, 18, 19, 21, 22, 27, 29-36, 38, 39, 43, 45, 46, 48-50, 52, 54, 55, 57, 59, 60, 62, 66-70, 73, 76-79, 82-88, 92, 95-97]

Excluded from Review:

Excluded during full text review [126-189]

- Not early PD
- Not methodologically generalizable
- Not reporting symptoms or impacts

Excluded during Abstract review:

- Biomarker/genetic study [190-261]
- Conference abstract [262]
- Digital monitoring, no symptoms [263-266]
- Imaging study [267-347]
- Modeling study [348-353]
- Not early PD population [354-370]
- Not reporting symptoms or impacts [371-439]
- Not a primary source [440-532]
- Out of data and not qualitative [533-541]
- Protocol/methods paper [542-550]
- Reference list - Review [551-569]
- Unrelated to Parkinson's [570-603]
- Validation study [604-638]
- Efficacy study (effect of X on Y) [639-705]
- Animal model study [706-763]
- Unsure/not usable [764]

[4] Port RJ, Rumsby M, Brown G, Harrison IF, Amjad A, Bale CJ (2021) People with Parkinson's Disease: What Symptoms Do They Most Want to Improve and How Does This Change with Disease Duration? *J Parkinsons Dis* **11**, 715-724.

[10] Mammen J, Speck R, Stebbins G, Müller M, Yang P, Campbell M, Cosman J, Crawford J, Dam T, Hellsten J, Jensen-Roberts S, Kostrzebski M, Simuni T, Ward Barowicz K, Cedarbaum J, Dorsey E, Stephenson D, Adams J (2022) Relative meaningfulness and impacts of symptoms in people with early-stage Parkinson’s disease. *(Preprint open access)*.

[11] Mestre TA, LaPelle N, Stebbins G (ND) A Patient Reported Outcome Assessment for Patients with Early Parkinson’s Disease. *Prepublication data*.

[12] Morel T, Cleanthous S, Andrejack J, Barker RA, Blavat G, Brooks W, Burns P, Cano S, Gallagher C, Gosden L, Siu C, Slagle AF, Trenam K, Boroojerdi B, Ratcliffe N, Schroeder K (2022) Patient Experience in Early-Stage Parkinson's Disease: Using a Mixed Methods Analysis to Identify Which Concepts Are Cardinal for Clinical Trial Outcome Assessment. *Neurol Ther* **11**, 1319-1340.

[13] Staunton H, Kelly K, Newton L, Leddin M, Rodriguez-Esteban R, Chaudhuri KR, Weintraub D, Postuma RB, Martinez-Martin P (2022) A Patient-Centered Conceptual Model of Symptoms and Their Impact in Early Parkinson's Disease: A Qualitative Study. *Journal of Parkinsons Disease* **12**, 137-151.

[15] Lerner A, Mammen J, Tyo M, Auinger P, Al-Rubayie R, Xiao Y, Marras C, Adam J ((ahead of press)) Fox Insight: Most bothersome symptoms in early-stage Parkinson’s disease. *Movement Disorders Clinical Practice*.

[16] Mammen J, Tyo M, Adams JL, Bale CJ, Xiao Y (2024) Understanding aspects of Parkinson’s Disease that are most important to treat from the perspective of patients and families. *Preprint*.

[17] Politis M, Wu K, Molloy S, Bain PG, Chaudhuri KR, Piccini P (2010) Parkinson's Disease Symptoms: The Patient's Perspective. *Movement Disorders* **25**, 1646-1651.

[18] Adams JL, Kangarloo T, Tracey B, O'Donnell P, Volfson D, Latzman RD, Zach N, Alexander R, Bergethon P, Cosman J, Anderson D, Best A, Severson J, Kostrzebski MA, Auinger P, Wilmot P, Pohlson Y, Waddell E, Jensen-Roberts S, Gong Y, Kilambi KP, Herrero TR, Ray Dorsey E, Parkinson Study Group Watch PDSI, Collaborators (2023) Using a smartwatch and smartphone to assess early Parkinson's disease in the WATCH-PD study. *NPJ Parkinsons Dis* **9**, 64.

[19] Adwani S, Yadav R, Kumar K, Chandra SR, Pal PK (2016) Neuropsychological profile in early Parkinson's disease: Comparison between patients with right side onset versus left side onset of motor symptoms. *Annals of Indian Academy of Neurology* **19**, 74-78.

[20] Amara AW, Chahine L, Seedorff N, Caspell-Garcia CJ, Coffey C, Simuni T, Marek K, Daegelel N, Tanner C, Simuni T, Coffey C, Kieburtz K, Wilsons R, Mollenhauer B, Galasko D, Foroud T, Chahine L, Siderowf A, Seibyl J, Toga A, Singleton A, Weintraub D, Trojanowski J, Shaw L, Tosun-Turgut D, Poston K, Bressman S, Merchant KM, Poewe W, Sherer T, Chowdhury S, Frasier M, Kopil C, Naito A, Arnedo V, Dorsey R, Casaceli C, Daegele N, Albani J, Caspell-Garcia C, Uribe L, Foster E, Long J, Seedorff N, Crawford K, Smiths DE, Casalin P, Malferrari G, Halter C, Heathers L, Russell D, Factor S, Hogarth P, Standaert D, Amara A, Hauser R, Jankovic J, Dahodwala N, Stern M, Hu SC, Todd G, Saunders-Pullman R, Richard I, Saint-

Hilaire MH, Seppi K, Shill H, Fernandez H, Trenkwalder C, Oertel W, Berg D, Brockman K, Wurster I, Rosenthal L, Tai Y, Pavese N, Barone P, Isaacson S, Espay A, Rowe D, Brandabur M, Tetrud J, Liang G, Iranzo A, Tolosa E, Marder K, Sanchez MD, Stefanis L, Marti MJ, Martinez JR, Corvol JC, Assly J, Brillman S, Giladi N, Smejdir D, Pelaggi J, Kausar F, Rees L, Sommerfield B, Freed A, Blair C, Williams K, Zimmerman G, Guthrie S, Rawlins A, Donhar L, Hunter C, Tran B, Darin A, Linder C, Baca M, Venkov H, Thomas CA, James R, Heim B, Deritis P, Sprenger F, Raymond D, Willeke D, Obradov Z, Mule J, Monahan N, Gauss K, Fontaine D, Szpak D, McCoy A, Dunlop B, Payne LM, Ainscough S, Carvajal L, Silverstein R, Espay K, Ranola M, Rezola EM, Santana HM, Stamelou M, Garrido A, Carvalho S, Kristiansen AG, Specketer K, Mirlman A, Facheris M, Soares H, Mintun MA, Cedarbaum J, Taylor P, Jennings D, Slieker L, McBride B, Watson C, Montagut E, Sheikh ZH, Bingol B, Forrat R, Sardi P, Fischer T, Reith AD, Egebjerg J, Larsen LF, Breysse N, Meulien D, Saba B, Kiyasova V, Min C, McAvoy T, Umek R, Iredale P, Edgerton J, De Sand S, Czech C, Boess F, Sevigny J, Kremer T, Grachev I, Merchant K, Avbersek A, Muglia P, Stewart A, Prashad R, Taucher J, Parkinsons Progression M (2019) Self-reported physical activity levels and clinical progression in early Parkinson's disease. *Parkinsonism & Related Disorders* **61**, 118-125.

[21] Amara AW, Chahine LM, Caspell-Garcia C, Long JD, Coffey C, Hogl B, Videnovic A, Iranzo A, Mayer G, Foldvary-Schaefer N, Postuma R, Oertel W, Lasch S, Marek K, Simuni T, Parkinson's Progression M (2017) Longitudinal assessment of excessive daytime sleepiness in early Parkinson's disease. *Journal of Neurology Neurosurgery and Psychiatry* **88**, 653-662.

[22] Baig F, Kelly MJ, Lawton MA, Ruffmann C, Rolinski M, Klein JC, Barber T, Lo C, Ben-Shlomo Y, Okai D, Hu MT (2019) Impulse control disorders in Parkinson disease and RBD A longitudinal study of severity. *Neurology* **93**, E675-E687.

[23] Baig F, Lawton M, Rolinski M, Ruffmann C, Nithi K, Evetts SG, Fernandes HR, Ben-Shlomo Y, Hu MTM (2015) Delineating Nonmotor Symptoms in Early Parkinson's Disease and First-Degree Relatives. *Movement Disorders* **30**, 1759-1766.

[24] Baig F, Lawton MA, Rolinski M, Ruffmann C, Klein JC, Nithi K, Okai D, Ben-Shlomo Y, Hu MTM (2017) Personality and addictive behaviours in early Parkinson's disease and REM sleep behaviour disorder. *Parkinsonism & Related Disorders* **37**, 72-78.

[25] Baschieri F, Sambati L, Guaraldi P, Barletta G, Cortelli P, Calandra-Buonaura G (2021) Neurogenic orthostatic hypotension in early stage Parkinson's disease: New insights from the first 105 patients of the BoProPark study. *Parkinsonism & Related Disorders* **93**, 12-18.

[26] Bega D, Luo S, Fernandez H, Chou K, Aminoff M, Parashos S, Walker H, Russell DS, Christine CW, Dhall R, Singer C, Bodis-Wollner I, Hamill R, Truong D, Mari Z, Glazmann S, Huang ML, Houston E, Simuni T, Investigators NPL (2015) Impact of Depression on Progression of Impairment and Disability in Early Parkinson's Disease. *Movement Disorders Clinical Practice* **2**, 371-378.

[27] Bhidayasiri R, Boonmongkol T, Thongchuam Y, Phumphid S, Kantachadvanich N, Panyakaew P, Jagota P, Plengsri R, Chokpatcharavate M, Phokaewvarangkul O (2020) Impact of disease stage and age at Parkinson's onset on patients' primary concerns: Insights for targeted management. *Plos One* **15**, 15.

[28] Choi SM, Cho SH, Choe Y, Kim BC (2023) Clinical determinants of apathy and its impact on health-related quality of life in early Parkinson disease. *Medicine* **102**, 4.

[29] Chua CY, Koh MRE, Chia NSY, Ng SYE, Saffari SE, Wen MC, Chen RYY, Choi XY, Heng DL, Neo SX, Tay KY, Au WL, Tan EK, Tan LCS, Xu ZY (2021) Subjective cognitive Complaints in early Parkinson ' s disease patients with normal cognition are associated with affective symptoms. *Parkinsonism & Related Disorders* **82**, 24-28.

[30] Diederich NJ, Rufra O, Pieri V, Hipp G, Vaillant M (2013) Lack of Polysomnographic Non-REM Sleep Changes in Early Parkinson's Disease. *Movement Disorders* **28**, 1443-1446.

[31] Diederich NJ, Sauvageot N, Pieri V, Hipp G, Vaillant M (2020) The Clinical Non-Motor Connectome in Early Parkinson's Disease. *Journal of Parkinsons Disease* **10**, 1797-1806.

[32] Dijkstra F, de Volder I, Viaene M, Cras P, Crosiers D (2022) Impaired bed mobility in prediagnostic and de novo Parkinson's disease. *Parkinsonism & Related Disorders* **98**, 47-52.

[33] Dlay JK, Duncan GW, Khoo TK, Williams-Gray CH, Breen DP, Barker RA, Burn DJ, Lawson RA, Yarnall AJ (2020) Progression of Neuropsychiatric Symptoms over Time in an Incident Parkinson's Disease Cohort (ICICLE-PD). *Brain Sciences* **10**, 12.

[34] Durcan R, Wiblin L, Lawson RA, Khoo TK, Yarnall AJ, Duncan GW, Brooks DJ, Pavese N, Burn DJ, Grp I-PS (2019) Prevalence and duration of non-motor symptoms in prodromal Parkinson's disease. *European Journal of Neurology* **26**, 979-985.

[35] Eriksson A, Tsitsi P, Vinding MC, Ingvar M, Svenningsson P, Lundqvist D (2022) Changes in Emotion Processing in Early Parkinson's Disease Reflect Disease Progression. *Neuropsychology* **36**, 206-215.

[36] Erro R, Picillo M, Vitale C, Amboni M, Moccia M, Longo K, Cozzolino A, Giordano F, De Rosa A, De Michele G, Pellecchia MT, Barone P, Erro R, Picillo M, Vitale C, Amboni M, Moccia M, Longo K, Cozzolino A, Giordano F (2013) Non-motor symptoms in early Parkinson's disease: a 2-year follow-up study on previously untreated patients. *Journal of Neurology, Neurosurgery & Psychiatry* **84**, 14-17.

[37] Fagerberg P, Klingelhoefer L, Bottai M, Langlet B, Kyritsis K, Rotter E, Reichmann H, Falkenburger B, Delopoulos A, Ioakimidis I (2020) Lower Energy Intake among Advanced vs. Early Parkinson's Disease Patients and Healthy Controls in a Clinical Lunch Setting: A Cross-Sectional Study. *Nutrients* **12**, 19.

[38] Hemphill L, Valenzuela Y, Luna K, Szymkowicz SM, Jones JD (2023) Synergistic associations of depressive symptoms and aging on cognitive decline in early Parkinson's disease. *Clin Park Relat Disord* **8**, 100192.

[39] Huang XX, Ng SYE, Chia NSY, Acharyya S, Setiawan F, Lu ZH, Ng E, Tay KY, Au WL, Tan EK, Tan LCS (2018) Serum uric acid level and its association with motor subtypes and non-motor symptoms in early Parkinson's disease: PALS study. *Parkinsonism & Related Disorders* **55**, 50-54.

[40] Isais-Millan S, Pina-Fuentes D, Guzman-Astorga C, Cervantes-Arriaga A, Rodriguez-Violante M (2016) Prevalence of neuropsychiatric disorders in drug-naive subjects with Parkinson's disease (PD). *Gaceta Medica De Mexico* **152**, 357-363.

[41] Jeancolas L, Mangone G, Petrovska-Delacretaz D, Benali H, Benkelfat BE, Arnulf I, Corvol JC, Vidailhet M, Lehericy S (2022) Voice characteristics from isolated rapid eye movement

sleep behavior disorder to early Parkinson's disease. *Parkinsonism & Related Disorders* **95**, 86-91.
[42] Kim M, Yoo S, Kim D, Cho JW, Kim JS, Ahn JH, Mun JK, Choi I, Lee SK, Youn J (2021) Extra-basal ganglia iron content and non-motor symptoms in drug-naive, early Parkinson's disease. *Neurological Sciences* **42**, 5297-5304.
[43] Koh MRE, Chua CY, Ng SYE, Chia NSY, Saffari SE, Chen RYY, Choi X, Heng DL, Neo SX, Tay KY, Au WL, Tan EK, Tan LCS, Xu ZY (2022) Poor sleep quality is associated with fatigue and depression in early Parkinson's disease: A longitudinal study in the PALS cohort. *Frontiers in Neurology* **13**, 9.
[44] Kwon KY, Park S, Lee M, Ju H, Im K, Joo BE, Lee KB, Roh H, Ahn MY (2020) Dizziness in patients with early stages of Parkinson's disease: Prevalence, clinical characteristics and implications. *Geriatrics & Gerontology International* **20**, 443-447.
[45] LaBelle DR, Walsh RR, Banks SJ (2017) Latent Cognitive Phenotypes in De Novo Parkinson's Disease: A Person-Centered Approach. *Journal of the International Neuropsychological Society* **23**, 551-563.
[46] Larsen JP, Dalen I, Pedersen KF, Tysnes OB (2017) The natural history of depressive symptoms in patients with incident Parkinson's disease: a prospective cohort study. *Journal of Neurology* **264**, 2401-2408.
[47] Lee SM, Kim M, Lee HM, Kwon KY, Kim HT, Koh SB (2014) Differential diagnosis of parkinsonism with visual inspection of posture and gait in the early stage. *Gait & Posture* **39**, 1138-1141.
[48] Liu R, Umbach DM, Peddada SD, Xu Z, Tröster AI, Huang X, Chen H (2015) Potential sex differences in nonmotor symptoms in early drug-naive Parkinson disease. *Neurology* **84**, 2107-2115.
[49] Lord S, Galna B, Coleman S, Burn D, Rochester L (2013) Mild depressive symptoms are associated with gait impairment in early Parkinson's disease. *Movement Disorders* **28**, 634-639.
[50] Malek N, Lawton MA, Grosset KA, Bajaj N, Barker RA, Burn DJ, Foltynie T, Hardy J, Morris HR, Williams NM, Ben-Shlomo Y, Wood NW, Grosset DG, Consortium PRC (2017) Autonomic Dysfunction in Early Parkinson's Disease: Results from the United Kingdom Tracking Parkinson's Study. *Movement Disorders Clinical Practice* **4**, 509-516.
[51] Marković V, Stanković I, Radovanović S, Petrović I, Ječmenica Lukić M, Dragašević Mišković N, Svetel M, Kostić V (2022) Gait alterations in Parkinson's disease at the stage of hemiparkinsonism-A longitudinal study. *PLoS One* **17**, e0269886.
[52] Martinez-Ramirez D, Velazquez-Avila ES, Almaraz-Espinoza A, Gonzalez-Cantu A, Vazquez-Elizondo G, Overa-Posada D, Cervantes-Arriaga A, Rodriguez-Violante M, Gonzalez-Gonzalez M (2020) Lower Urinary Tract and Gastrointestinal Dysfunction Are Common in Early Parkinson's Disease. *Parkinsons Disease* **2020**, 8.
[53] Meira B, Lhommee E, Schmitt E, Klinger H, Bichon A, Pelissier P, Anheim M, Tranchant C, Fraix V, Meoni S, Durif F, Houeto JL, Azulay JP, Moro E, Thobois S, Krack P, Castrioto A, Honeymoon study g (2022) Early Parkinson's Disease Phenotypes Tailored by Personality, Behavior, and Motor Symptoms. *J Parkinsons Dis* **12**, 1665-1676.

[54] Moguel-Cobos G, Saldivar C, Goslar PW, Shill HA (2020) The Relationship Between Social Anxiety Disorder and Motor Symptoms of Parkinson Disease: A Pilot Study. *Psychosomatics* **61**, 321-326.
[55] Mollenhauer B, Zimmermann J, Sixel-Döring F, Focke NK, Wicke T, Ebentheuer J, Schaumburg M, Lang E, Friede T, Trenkwalder C, Sixel-Döring F (2019) Baseline predictors for progression 4 years after Parkinson's disease diagnosis in the De Novo Parkinson Cohort (DeNoPa). *Movement Disorders* **34**, 67-77.
[56] Moreau C, Devos D, Baille G, Delval A, Tard C, Perez T, Danel-Buhl N, Seguy D, Labreuche J, Duhamel A, Delliaux M, Dujardin K, Defebvre L (2016) Are Upper-Body Axial Symptoms a Feature of Early Parkinson's Disease? *Plos One* **11**, 13.
[57] Morris R, Lord S, Lawson RA, Coleman S, Galna B, Duncan GW, Khoo TK, Yarnall AJ, Burn DJ, Rochester L (2017) Gait Rather Than Cognition Predicts Decline in Specific Cognitive Domains in Early Parkinson's Disease. *Journals of Gerontology Series a-Biological Sciences and Medical Sciences* **72**, 1656-1662.
[58] Muller B, Assmus J, Herlofson K, Larsen JP, Tysnes OB (2013) Importance of motor vs. non-motor symptoms for health-related quality of life in early Parkinson's disease. *Parkinsonism & Related Disorders* **19**, 1027-1032.
[59] Muller B, Assmus J, Larsen JP, Haugarvoll K, Skeie GO, Tysnes OB, ParkWest Study G (2013) Autonomic symptoms and dopaminergic treatment in de novo Parkinson's disease. *Acta Neurologica Scandinavica* **127**, 290-294.
[60] Naisby J, Lawson RA, Galna B, Alcock L, Burn DJ, Rochester L, Yarnall AJ (2021) Trajectories of pain over 6 years in early Parkinson's disease: ICICLE-PD. *Journal of Neurology* **268**, 4759-4767.
[61] Oh YS, Kim JS, Park IS, Song IU, Son YM, Park JW, Yang DW, Kim HT, Lee KS (2014) Association between nocturnal/supine hypertension and restless legs syndrome in patients with Parkinson's disease. *Journal of the Neurological Sciences* **344**, 186-189.
[62] Ongre SO, Larsen JP, Tysnes OB, Herlofson K (2017) Fatigue in early Parkinson's disease: the Norwegian ParkWest study. *European Journal of Neurology* **24**, 105-111.
[63] Ou RW, Hou YB, Liu KC, Lin JY, Jiang Z, Wei QQ, Zhang LY, Cao B, Zhao B, Song W, Shang HF (2021) Progression of Fatigue in Early Parkinson's Disease: A 3-Year Prospective Cohort Study. *Frontiers in Aging Neuroscience* **13**, 8.
[64] Ou RW, Lin JY, Liu KC, Jiang Z, Wei QQ, Hou YB, Zhang LY, Cao B, Zhao B, Song W, Shang HF (2021) Evolution of Apathy in Early Parkinson's Disease: A 4-Years Prospective Cohort Study. *Frontiers in Aging Neuroscience* **12**, 9.
[65] Pan CX, Ren JR, Hua P, Yan L, Yu M, Wang YJ, Zhou GY, Zhang RG, Chen J, Liu WG (2021) Subjective Cognitive Complaints in Newly-Diagnosed Parkinson's Disease With and Without Mild Cognitive Impairment. *Frontiers in Neuroscience* **15**, 8.
[66] Paracha M, Herbst K, Kieburtz K, Venuto CS (2022) Prevalence and Incidence of Nonmotor Symptoms in Individuals with and Without Parkinson's Disease. *Movement Disorders Clinical Practice* **9**, 961-966.
[67] Pellicano C, Assogna F, Cravello L, Langella R, Caltagirone C, Spalletta G, Pontieri FE (2015) Neuropsychiatric and cognitive symptoms and body side of onset of parkinsonism in unmedicated Parkinson's disease patients. *Parkinsonism & Related Disorders* **21**, 1096-1100.

[68] Picillo M, Erro R, Amboni M, Longo K, Vitale C, Moccia M, Pierro A, Scannapieco S, Santangelo G, Spina E, Orefice G, Barone P, Pellecchia MT (2014) Gender differences in non-motor symptoms in early Parkinson's disease: A 2-years follow-up study on previously untreated patients. *Parkinsonism & Related Disorders* **20**, 850-854.

[69] Picillo M, Palladino R, Erro R, Alfano R, Colosimo C, Marconi R, Antonini A, Barone P (2021) The PRIAMO study: age- and sex-related relationship between prodromal constipation and disease phenotype in early Parkinson's disease. *Journal of Neurology* **268**, 448-454.

[70] Podgorny PJ, Suchowersky O, Romanchuk KG, Feasby TE (2016) Evidence for small fiber neuropathy in early Parkinson's disease. *Parkinsonism & Related Disorders* **28**, 94-99.

[71] Roggendorf J, Chen S, Baudrexel S, van de Loo S, Seifried C, Hilker R (2012) Arm swing asymmetry in Parkinson's disease measured with ultrasound based motion analysis during treadmill gait. *Gait & Posture* **35**, 116-120.

[72] Rolinski M, Szewczyk-Krolikowski K, Tomlinson PR, Nithi K, Talbot K, Ben-Shlomo Y, Hu MT (2014) REM sleep behaviour disorder is associated with worse quality of life and other non-motor features in early Parkinson's disease. *J Neurol Neurosurg Psychiatry* **85**, 560-566.

[73] Santangelo G, Vitale C, Trojano L, Picillo M, Moccia M, Pisano G, Pezzella D, Cuoco S, Erro R, Longo K, Pellecchia MT, Amboni M, De Rosa A, De Michele G, Barone P (2015) Relationship between apathy and cognitive dysfunctions in de novo untreated Parkinson's disease: a prospective longitudinal study. *European Journal of Neurology* **22**, 253-260.

[74] Santos-Garcia D, Fonticoba TD, Castro ES, Diaz AA, McAfee D, Catalan MJ, Alonso-Frech F, Villanueva C, Jesus S, Mir P, Aguilar M, Pastor P, Caldentey JG, Peyret EE, Planellas LL, Marti MJ, Caballol N, Vara JH, Andres GM, Cabo I, Rivera MAA, Manzanares LL, Redondo N, Martinez-Martin P, Grp CS (2020) Non-motor symptom burden is strongly correlated to motor complications in patients with Parkinson's disease. *European Journal of Neurology* **27**, 1210-1223.

[75] Schindlbeck KA, Mehl A, Geffe S, Benik S, Tutuncu S, Klostermann F, Marzinzik F (2016) Somatosensory symptoms in unmedicated de novo patients with idiopathic Parkinson's disease. *J Neural Transm (Vienna)* **123**, 211-217.

[76] Serra MC, Landry A, Juncos JL, Markland AD, Burgio KL, Goode PS, Johnson TM, Vaughan CP (2018) Increased odds of bladder and bowel symptoms in early Parkinson's disease. *Neurourology and Urodynamics* **37**, 1344-1348.

[77] Siciliano M, Trojano L, De Micco R, Giordano A, Russo A, Tedeschi G, Chiorri C, Tessitore A (2020) Predictors of fatigue severity in early, de novo Parkinson disease patients: A 1-year longitudinal study. *Parkinsonism & Related Disorders* **79**, 3-8.

[78] Simuni T, Caspell-Garcia C, Coffey C, Chahine LM, Lasch S, Oertel WH, Mayer G, Hogl B, Postuma R, Videnovic A, Amara AW, Marek K, Investigators PSWgobotP (2015) Correlates of excessive daytime sleepiness in de novo Parkinson's disease: A case control study. *Mov Disord* **30**, 1371-1381.

[79] Simuni T, Caspell-Garcia C, Coffey CS, Weintraub D, Mollenhauer B, Lasch S, Tanner CM, Jennings D, Kieburtz K, Chahine LM, Marek K (2018) Baseline prevalence and longitudinal evolution of non-motor symptoms in early Parkinson's disease: the PPMI cohort. *Journal of Neurology Neurosurgery and Psychiatry* **89**, 78-88.

[80] Skrabal D, Rusz J, Novotny M, Sonka K, Ruzicka E, Dusek P, Tykalova T (2022) Articulatory undershoot of vowels in isolated REM sleep behavior disorder and early Parkinson's disease. *Npj Parkinsons Disease* **8**, 7.

[81] Song Y, Gu ZQ, An J, Chan P, Chinese Parkinson Study G (2014) Gender differences on motor and non-motor symptoms of de novo patients with early Parkinson's disease. *Neurological Sciences* **35**, 1991-1996.

[82] Stankovic I, Petrovic I, Pekmezovic T, Markovic V, Stojkovic T, Dragasevic-Miskovic NA, Svetel M, Kostic V (2019) Longitudinal assessment of autonomic dysfunction in early Parkinson's disease. *Parkinsonism & Related Disorders* **66**, 74-79.

[83] Stankovic I, Stefanova E, Tomic A, Lukic MJ, Stojkovic T, Markovic V, Stojmenovic GM, Kresojevic N, Svetel M, Kostic V (2016) Psychiatric Symptoms in the Initial Motor Stage of Parkinson's Disease. *Journal of Neuropsychiatry and Clinical Neurosciences* **28**, 205-210.

[84] Sung HY, Park JW, Kim JS (2014) The Frequency and Severity of Gastrointestinal Symptoms in Patients with Early Parkinson's Disease. *Journal of Movement Disorders* **7**, 7-12.

[85] Szewczyk-Krolikowski K, Tomlinson P, Nithi K, Wade-Martins R, Talbot K, Ben-Shlomo Y, Hu MTM (2014) The influence of age and gender on motor and non-motor features of early Parkinson's disease: Initial findings from the Oxford Parkinson Disease Center (OPDC) discovery cohort. *Parkinsonism & Related Disorders* **20**, 99-105.

[86] Tholfsen LK, Larsen JP, Schulz J, Tysnes OB, Gjerstad MD (2015) Development of excessive daytime sleepiness in early Parkinson disease. *Neurology* **85**, 162-168.

[87] Tholfsen LK, Larsen JP, Schulz J, Tysnes OB, Gjerstad MD (2017) Changes in insomnia subtypes in early Parkinson disease. *Neurology* **88**, 352-358.

[88] Tosin MHS, Simuni T, Stebbins GT, Cedarbaum JM (2022) Tracking Emergence of New Motor and Non-Motor Symptoms Using the MDS-UPDRS: A Novel Outcome Measure for Early Parkinson's Disease? *Journal of Parkinsons Disease* **12**, 1345-1351.

[89] Tveiten OV, Skeie GO, Haugarvoll K, Muller B, Larsen JP, Tysnes OB (2013) Treatment in early Parkinson's disease: the Norwegian ParkWest study. *Acta Neurologica Scandinavica* **128**, 107-113.

[90] Wu L, Mu N, Yang F, Zang J, Zheng JP (2016) A study of the non-motor symptoms in early Parkinson's disease with olfactory deficits. *Eur Rev Med Pharmacol Sci* **20**, 3857-3862.

[91] Wu Y, Guo XY, Wei QQ, Ou RW, Song W, Cao B, Zhao B, Shang HF (2016) Non-motor symptoms and quality of life in tremor dominant vs postural instability gait disorder Parkinson's disease patients. *Acta Neurologica Scandinavica* **133**, 330-337.

[92] Yang HJ, Kim YE, Yun JY, Kim HJ, Jeon BS (2014) Identifying the Clusters within Nonmotor Manifestations in Early Parkinson's Disease by Using Unsupervised Cluster Analysis. *Plos One* **9**, 5.

[93] Yoo SW, Kim JS, Oh YS, Ryu DW, Lee KS (2019) Trouble Concentrating is an Easily Overlooked Symptom of Orthostatic Hypotension in Early Parkinson's Disease. *J Parkinsons Dis* **9**, 405-411.

[94] Zhang H, Gu ZQ, An J, Wang CD, Chan P (2014) Non-Motor Symptoms in Treated and Untreated Chinese Patients with Early Parkinson's Disease. *Tohoku Journal of Experimental Medicine* **232**, 129-136.

[95] Zhang H, Yin X, Ouyang Z, Chen J, Zhou S, Zhang C, Pan X, Wang S, Yang J, Feng Y, Yu P, Zhang Q (2016) A prospective study of freezing of gait with early Parkinson disease in Chinese patients. *Medicine (Baltimore)* **95**, e4056.
[96] Zhou MX, Wang Q, Lin Y, Xu Q, Wu L, Chen YJ, Jiang YH, He Q, Zhao L, Dong YR, Liu JR, Chen W (2022) Oculomotor impairments in de novo Parkinson's disease. *Frontiers in Aging Neuroscience* **14**, 9.
[97] Zucco GM, Rovatti F, Stevenson RJ (2015) Olfactory asymmetric dysfunction in early Parkinson patients affected by unilateral disorder. *Frontiers in Psychology* **6**, 4.
[126] Erro R, Vitale C, Amboni M, Picillo M, Moccia M, Longo K, Santangelo G, De Rosa A, Allocca R, Giordano F, Orefice G, De Michele G, Santoro L, Pellecchia MT, Barone P (2013) The Heterogeneity of Early Parkinson's Disease: A Cluster Analysis on Newly Diagnosed Untreated Patients. *Plos One* **8**, 8.
[127] Khoo TK, Yarnall AJ, Duncan GW, Coleman S, O'Brien JT, Brooks DJ, Barker RA, Burn DJ (2013) The spectrum of nonmotor symptoms in early Parkinson disease. *Neurology* **80**, 276-281.
[128] Picillo M, Amboni M, Erro R, Vitale C, Longo K, Pellecchia MT, Cozzolino A, Moccia M, Allocca R, Barone P (2013) Segmental progression of cardinal motor symptoms in Parkinson's disease: A pilot study suggesting a practical approach to rate disease course in the early stages. *Parkinsonism & Related Disorders* **19**, 1143-1148.
[129] de la Riva P, Smith K, Xie SX, Weintraub D (2014) Course of psychiatric symptoms and global cognition in early Parkinson disease. *Neurology* **83**, 1096-1103.
[130] Duncan GW, Khoo TK, Yarnall AJ, O'Brien JT, Coleman SY, Brooks DJ, Barker RA, Burn DJ (2014) Health-related quality of life in early Parkinson's disease: The impact of nonmotor symptoms. *Movement Disorders* **29**, 195-202.
[131] Frandsen R, Kjellberg J, Ibsen R, Jennum P (2014) Morbidity in early Parkinson's disease and prior to diagnosis. *Brain and Behavior* **4**, 446-452.
[132] Hanoglu L, Hakyemez HA, Ozer F, Ozben S, Demirci S, Akarsu EO (2014) Relation between Olfactory Dysfunction and Episodic Verbal Memory in Early Parkinson's Disease. *Noropsikiyatri Arsivi-Archives of Neuropsychiatry* **51**, 389-394.
[133] Lawson RA, Yarnall AJ, Duncan GW, Khoo TK, Breen DP, Barker RA, Collerton D, Taylor JP, Burn DJ (2014) Severity of mild cognitive impairment in early Parkinson's disease contributes to poorer quality of life. *Parkinsonism & Related Disorders* **20**, 1071-1075.
[134] Lee WJ, Chang YY, Lin JJ, Sung YF, Li JY, Wang SJ, Chen RS, Yang YH, Hu CJ, Tsai CH, Wang HC, Wu SL, Chang MH, Fuh JL (2014) Comparison of activities of daily living impairments in Parkinson's disease patients as defined by the Pill Questionnaire and assessments by neurologists. *Journal of Neurology Neurosurgery and Psychiatry* **85**, 969-973.
[135] Mahajan A, Rosenthal LS, Gamaldo C, Salas RE, Pontone GM, McCoy A, Umeh C, Mari Z (2014) REM Sleep Behavior and Motor Findings in Parkinson's Disease: A Cross-sectional Analysis. *Tremor and Other Hyperkinetic Movements* **4**, 6.
[136] Bega D, Kim S, Zhang Y, Elm J, Schneider J, Hauser R, Fraser A, Simuni T (2015) Predictors of Functional Decline in Early Parkinson's Disease: NET-PD LS1 Cohort. *J Parkinsons Dis* **5**, 773-782.
[137] Hong JY, Sunwoo MK, Ham JH, Lee JJ, Lee PH, Sohn YH (2015) Apathy and Olfactory Dysfunction in Early Parkinson's Disease. *Journal of Movement Disorders* **8**, 21-25.

[138] Lieberman A, Deep A, Dhall R, Tran A, Liu MJ (2015) Early Freezing of Gait: Atypical versus Typical Parkinson Disorders. *Parkinsons Disease* **2015**, 5.

[139] Louter M, Maetzler W, Prinzen J, van Lummel RC, Hobert M, Arends JB, Bloem BR, Streffer J, Berg D, Overeem S, Liepelt-Scarfone I (2015) Accelerometer-based quantitative analysis of axial nocturnal movements differentiates patients with Parkinson's disease, but not high-risk individuals, from controls. *J Neurol Neurosurg Psychiatry* **86**, 32-37.

[140] Zis P, Martinez-Martin P, Sauerbier A, Rizos A, Sharma JC, Worth PF, Sophia R, Silverdale M, Chaudhuri KR (2015) Non-motor symptoms burden in treated and untreated early Parkinson's disease patients: argument for non-motor subtypes. *European Journal of Neurology* **22**, 1145-1150.

[141] Bjornestad A, Tysnes OB, Larsen JP, Alves G (2016) Loss of independence in early Parkinson disease A 5-year population-based incident cohort study. *Neurology* **87**, 1599-1606.

[142] Defazio G, Guerrieri M, Liuzzi D, Gigante AF, di Nicola V (2016) Assessment of voice and speech symptoms in early Parkinson's disease by the Robertson dysarthria profile. *Neurological Sciences* **37**, 443-449.

[143] Fullard ME, Tran BC, Xie SX, Toledo JB, Scordia C, Linder C, Purri R, Weintraub D, Duda JE, Chahine LM, Morley JF (2016) Olfactory impairment predicts cognitive decline in early Parkinson's disease. *Parkinsonism & Related Disorders* **25**, 45-51.

[144] Lamont RM, Morris ME, Woollacott MH, Brauer SG (2016) Ambulatory Activity in People with Early Parkinson's Disease. *Brain Impairment* **17**, 87-98.

[145] Olszewska DA, Fearon C, Lynch T (2016) Loss of visual feedback revealing motor impairment- an early symptom of Parkinson's disease in two Irish farmers. *J Clin Mov Disord* **3**, 12.

[146] Teive HAG, Bertucci DC, Munhoz RP (2016) Unusual motor and non-motor symptoms and signs in the early stage of Parkinson's disease. *Arquivos De Neuro-Psiquiatria* **74**, 781-784.

[147] Eisinger RS, Hess CW, Martinez-Ramirez D, Almeida L, Foote KD, Okun MS, Gunduz A (2017) Motor subtype changes in early Parkinson's disease. *Parkinsonism Relat Disord* **43**, 67-72.

[148] Fereshtehnejad SM, Zeighami Y, Dagher A, Postuma RB (2017) Clinical criteria for subtyping Parkinson's disease: biomarkers and longitudinal progression. *Brain* **140**, 1959-1976.

[149] Hindle JV, Martin-Forbes PA, Martyr A, Bastable AJM, Pye KL, Gathercole VCM, Thomas EM, Clare L (2017) The effects of lifelong cognitive lifestyle on executive function in older people with Parkinson's disease. *International Journal of Geriatric Psychiatry* **32**, E157-E165.

[150] Kluger BM, Pedersen KF, Tysnes OB, Ongre SO, Oygarden B, Herlofson K (2017) Is fatigue associated with cognitive dysfunction in early Parkinson's disease? *Parkinsonism & Related Disorders* **37**, 87-91.

[151] Picillo M, Palladino R, Barone P, Erro R, Colosimo C, Marconi R, Morgante L, Antonini A, Grp PS (2017) The PRIAMO study: urinary dysfunction as a marker of disease progression in early Parkinson's disease. *European Journal of Neurology* **24**, 788-795.

[152] Toomsoo T, Randver R, Liepelt-Scarfone I, Kadastik-Eerme L, Asser T, Rubanovits I, Berg D, Taba P (2017) Prevalence of depressive symptoms and their association with brainstem

raphe echogenicity in patients with Parkinson's disease and non-PD controls. *Psychiatry Research-Neuroimaging* **268**, 45-49.

[153] Barrett MJ, Blair JC, Sperling SA, Smolkin ME, Druzgal TJ (2018) Baseline symptoms and basal forebrain volume predict future psychosis in early Parkinson disease. *Neurology* **90**, E1618-E1626.

[154] Guan XX, Wang YC, Li Q, Wei M, Chen LL, Cheng OM (2018) Analysis of the clinical features of early Parkinson's disease with comparatively integrated intestinal function. *Neurological Sciences* **39**, 1847-1856.

[155] Hughes KC, Gao X, Baker JM, Stephen C, Kim IY, Valeri L, Schwarzschild MA, Ascherio A (2018) Non-motor features of Parkinson's disease in a nested case-control study of US men. *Journal of Neurology Neurosurgery and Psychiatry* **89**, 1288-1295.

[156] Kandaswamy D, MuthuKumar M, Alexander M, Prabhu K, Gowri SM, Krothapalli SB (2018) Quantitative Assessment of Hand Dysfunction in Patients with Early Parkinson's Disease and Focal Hand Dystonia. *Journal of Movement Disorders* **11**, 35-44.

[157] Park HR, Youn J, Cho JW, Oh ES, Kim JS, Park S, Jang W, Park JS (2018) Characteristic Motor and Nonmotor Symptoms Related to Quality of Life in Drug-Naive Patients with Late-Onset Parkinson Disease. *Neurodegener Dis* **18**, 19-25.

[158] Wu JQ, Li P, Stavitsky Gilbert K, Hu K, Cronin-Golomb A (2018) Circadian Rest-Activity Rhythms Predict Cognitive Function in Early Parkinson's Disease Independently of Sleep. *Mov Disord Clin Pract* **5**, 614-619.

[159] Chahine LM, Siderowf A, Barnes J, Seedorff N, Caspell-Garcia C, Simuni T, Coffey CS, Galasko D, Mollenhauer B, Arnedo V, Daegele N, Frasier M, Tanner C, Kieburtz K, Marek K, Seibyl J, Coffey C, Tosun-Turgut D, Shaw L, Trojanowski J, Singleton A, Toga A, Chahine L, Poewe W, Foroud T, Poston K, Sherer T, Chowdhury S, Kopil C, Casaceli C, Dorsey R, Wilson R, Mahes S, Salerno C, Crawford K, Casalin P, Malferrari G, Weisz MG, Orr-Urtreger A, Montine T, Russell D, Dahodwala N, Giladi N, Factor S, Hogarth P, Standaert D, Hauser R, Jankovic J, Saint-Hilaire M, Richard I, Shprecher D, Fernandez H, Brockmann K, Rosenthal L, Barone P, Espay A, Rowe D, Marder K, Santiago A, Bressman S, Hu SC, Isaacson S, Corvol JC, Martinez JR, Tolosa E, Tai Y, Politis M, Smejdir D, Rees L, Williams K, Kausar F, Richardson W, Willeke D, Peacock S, Sommerfeld B, Freed A, Wakeman K, Blair C, Guthrie S, Harrell L, Hunter C, Thomas CA, James R, Zimmerman G, Brown V, Mule J, Hilt E, Ribb K, Ainscough S, Wethington M, Ranola M, Santana HM, Moreno J, Raymond D, Speketer K, Carvajal L, Carvalho S, Croitoru I, Garrido A, Payne LM, Viswanth V, Severt L, Facheris M, Soares H, Mintun MA, Cedarbaum J, Taylor P, Biglan K, Vandenbroucke E, Sheikh ZH, Bingol B, Fischer T, Sardi P, Forrat R, Reith A, Egebjerg J, Hillert GA, Saba B, Min C, Umek R, Mather J, De Santi S, Post A, Boess F, Taylor K, Grachev I, Avbersek A, Muglia P, Merchant K, Tauscher J, Parkinsons Progression Markers I (2019) Predicting Progression in Parkinson's Disease Using Baseline and 1-Year Change Measures. *Journal of Parkinsons Disease* **9**, 665-679.

[160] Huang X, Ng SYE, Chia NSY, Setiawan F, Tay KY, Au WL, Tan EK, Tan LCS (2019) Non-motor symptoms in early Parkinson's disease with different motor subtypes and their associations with quality of life. *European Journal of Neurology* **26**, 400-406.

[161] Kim R, Yoo D, Im JH, Kim HJ, Jeon B (2019) REM sleep behavior disorder predicts functional dependency in early Parkinson's disease. *Parkinsonism & Related Disorders* **66**, 138-142.

[162] Li Y, Zhang H, Mao W, Liu XN, Hao SW, Zhou YT, Ma JH, Gu ZQ, Chan P (2019) Visual dysfunction in patients with idiopathic rapid eye movement sleep behavior disorder. *Neuroscience Letters* **709**, 5.

[163] Ma SY, Zhang YS, Liu N, Xiao WZ, Li SQ, Zhang GY, Zhou XL, Munte TF, Ye Z (2019) Altered transposition asymmetry in serial ordering in early Parkinson's disease. *Parkinsonism & Related Disorders* **62**, 62-67.

[164] Picillo M, Palladino R, Erro R, Colosimo C, Marconi R, Antonini A, Barone P, Grp PS (2019) The PRIAMO study: active sexual life is associated with better motor and non-motor outcomes in men with early Parkinson's disease. *European Journal of Neurology* **26**, 1327-1333.

[165] Romosan AM, Dehelean L, Romosan RS, Andor M, Bredicean AC, Simu MA (2019) Affective theory of mind in Parkinson's disease: the effect of cognitive performance. *Neuropsychiatric Disease and Treatment* **15**, 2521-2535.

[166] Ryu DW, Kim JS, Yoo SW, Oh YS, Lee KS (2019) The Impact of Impulsivity on Quality of Life in Early Drug-Naive Parkinson's Disease Patients. *Journal of Movement Disorders* **12**, 172-176.

[167] Stuart S, Lawson RA, Yarnall AJ, Nell J, Alcock L, Duncan GW, Khoo TK, Barker RA, Rochester L, Burn DJ, O'Brien JT, Brooks DJ, Wesnes KA, Robbins TW, Chinnery PF, Johnston F, McDonald C, Sleeman I, Rowe JB, Williams-Gray C, Breen D, Cummins GA, Evans J, Grp I-PS (2019) Pro-Saccades Predict Cognitive Decline in Parkinson's Disease: ICICLE-PD. *Movement Disorders* **34**, 1690-1698.

[168] Mills KA, Schneider RB, Saint-Hilaire M, Ross GW, Hauser RA, Lang AE, Halverson MJ, Oakes D, Eberly S, Litvan I, Blindauer K, Aquino C, Simuni T, Marras C (2020) Cognitive impairment in Parkinson's disease: Associations between subjective and objective cognitive decline in a large longitudinal study. *Parkinsonism & Related Disorders* **80**, 127-132.

[169] Park JH, Lee SH, Kim Y, Park SW, Byeon GH, Jang JW (2020) Depressive symptoms are associated with worse cognitive prognosis in patients with newly diagnosed idiopathic Parkinson disease. *Psychogeriatrics:The Official Journal of the Japanese Psychogeriatric Society* **20**, 880-890.

[170] Baraldi MA, Avanzino L, Pelosin E, Domaneschi F, Paola SD, Lagravinese G (2021) Pragmatic abilities in early Parkinson?s disease. *Brain and Cognition* **150**, 16.

[171] Chao JY, Xiong KP, Zhuang S, Zhang JR, Huang JY, Li J, Mao CJ, Wu HH, Wang JY, Liu CF (2021) [Relationship between emotional apathy and motor symptoms, sleep and cognitive function in patients with early Parkinson's disease]. *Zhonghua Yi Xue Za Zhi* **101**, 2792-2797.

[172] Kwon KY, Lee EJ, Lee M, Ju H, Im K (2021) Impact of motor subtype on non-motor symptoms and fall-related features in patients with early Parkinson's disease. *Geriatrics & Gerontology International* **21**, 416-420.

[173] Kwon KY, Park S, Lee EJ, Lee M, Ju H (2021) Association of fall risk factors and non-motor symptoms in patients with early Parkinson's disease. *Scientific Reports* **11**, 6.

[174] Liguori S, Moretti A, Palomba A, Paoletta M, Gimigliano F, De Micco R, Siciliano M, Tessitore A, Iolascon G (2021) Non-motor impairments affect walking kinematics in Parkinson disease patients: A cross-sectional study. *Neurorehabilitation* **49**, 481-489.
[175] Liu YP, Lawton MA, Lo C, Bowring F, Klein JC, Querejeta-Coma A, Scotton S, Welch J, Razzaque J, Barber T, Ben-Shlomo Y, Hu MT (2021) Longitudinal Changes in Parkinson's Disease Symptoms with and Without Rapid Eye Movement Sleep Behavior Disorder: The Oxford Discovery Cohort Study. *Movement Disorders* **36**, 2821-2832.
[176] Ng SYE, Chia NSY, Abbas MM, Saffari ES, Choi XY, Heng DL, Xu ZY, Tay KY, Au WL, Tan EK, Tan LCS (2021) Physical Activity Improves Anxiety and Apathy in Early Parkinson's Disease: A Longitudinal Follow-Up Study. *Frontiers in Neurology* **11**, 8.
[177] Ou RW, Hou YB, Wei QQ, Lin JY, Liu KC, Zhang LY, Jiang Z, Cao B, Zhao B, Song W, Shang HF (2021) Longitudinal evolution of non-motor symptoms in early Parkinson's disease: a 3-year prospective cohort study. *Npj Parkinsons Disease* **7**, 6.
[178] Yoo HS, Lee S, Jeong SH, Ye BS, Sohn YH, Yun M, Lee PH (2021) Clinical and Dopamine Depletion Patterns in Hyposmia- and Dysautonomia-Dominant Parkinson's Disease. *J Parkinsons Dis* **11**, 1703-1713.
[179] Julio F, Ribeiro MJ, Morgadinho A, Sousa M, van Asselen M, Simoes MR, Castelo-Branco M, Januario C (2022) Cognition, function and awareness of disease impact in early Parkinson's and Huntington's disease. *Disabil Rehabil* **44**, 921-939.
[180] Kwon KY, Park S, Lee EJ, Lee M, Ju H (2022) Impact of subjective dizziness on motor and non-motor symptoms in patients with early stages of Parkinson's disease. *Journal of Integrative Neuroscience* **21**, 6.
[181] Ma JN, Dou KX, Liu RZ, Liao YJ, Yuan ZQ, Xie AM (2022) Associations of Sleep Disorders With Depressive Symptoms in Early and Prodromal Parkinson's Disease. *Frontiers in Aging Neuroscience* **14**, 13.
[182] Martinez-Nunez AE, Latack K, Situ-Kcomt M, Mahajan A (2022) Olfaction and apathy in early idiopathic Parkinson's disease. *Journal of the Neurological Sciences* **439**, 3.
[183] Picillo M, Lafontant DE, Bressman S, Caspell-Garcia C, Coffey C, Cho HR, Burghardt EL, Dahodwala N, Saunders-Pullman R, Tanner CM, Amara AW, Parkinson's Progression Markers I (2022) Sex-Related Longitudinal Change of Motor, Non-Motor, and Biological Features in Early Parkinson's Disease. *Journal of Parkinsons Disease* **12**, 421-436.
[184] Skjaerbaek C, Knudsen K, Kinnerup M, Hansen KV, Borghammer P (2022) Intestinal Transit in Early Moderate Parkinson's Disease Correlates with Probable RBD: Subclinical Esophageal Dysmotility Does Not Correlate. *Parkinsons Disease* **2022**, 8.
[185] Szymkowicz SM, Jones JD, Timblin H, Ryczek CA, Taylor WD, May PE (2022) Apathy as a Within-Person Mediator of Depressive Symptoms and Cognition in Parkinson's Disease: Longitudinal Mediation Analyses. *Am J Geriatr Psychiatry* **30**, 664-674.
[186] Zolfaghari S, Thomann AE, Lewandowski N, Trundell D, Lipsmeier F, Pagano G, Taylor KI, Postuma RB (2022) Self-Report versus Clinician Examination in Early Parkinson's Disease. *Movement Disorders* **37**, 585-597.
[187] Meng D, Jin Z, Wang Y, Fang B (2023) Longitudinal cognitive changes in patients with early Parkinson's disease and neuropsychiatric symptoms. *CNS Neurosci Ther*.
[188] Morel T, Cleanthous S, Andrejack J, Barker RA, Biagioni M, Blavat G, Bloem BR, Boroojerdi B, Brooks W, Burns P, Cano S, Gallagher C, Gosden L, Siu C, Slagle AF, Ratcliffe N,

Schroeder K (2023) Development and early qualitative evidence of two novel patient-reported outcome instruments to assess daily functioning in people with early-stage Parkinson's. *J Patient Rep Outcomes* **7**, 40.
[189] Turner TH, Lench DH, Adams R, Wilson S, Marsicano C, Rodriguez-Porcel F (2023) Are Standardized Tests Sensitive to Early Cognitive Change in Parkinson's Disease? *Psychopharmacol Bull* **53**, 19-29.
[190] Ba MW, Yu GP, Kong M, Liang H, Yu L (2018) CSF A beta(1-42) level is associated with cognitive decline in early Parkinson's disease with rapid eye movement sleep behavior disorder. *Translational Neurodegeneration* **7**, 9.
[191] Baez S, Herrera E, Trujillo C, Cardona JF, Diazgranados JA, Pino M, Santamaria-Garcia H, Ibanez A, Garcia AM (2020) Classifying Parkinson's Disease Patients With Syntactic and Socio-emotional Verbal Measures. *Frontiers in Aging Neuroscience* **12**, 11.
[192] Beauchamp LC, Chan J, Hung LW, Padman BS, Vella LJ, Liu XM, Coleman B, Bush AI, Lazarou M, Hill AF, Jacobson L, Barnham KJ (2018) Ablation of tau causes an olfactory deficit in a murine model of Parkinson's disease. *Acta Neuropathologica Communications* **6**, 12.
[193] Beigi M, Wilkinson L, Gobet F, Parton A, Jahanshahi M (2016) Levodopa medication improves incidental sequence learning in Parkinson's disease. *Neuropsychologia* **93**, 53-60.
[194] Bin-Nun A, Shchors I, Abu-Omar R, Kasirer Y, Mimouni F, Hammerman C (2022) A simple noninvasive biomarker can reflect both the acute and chronic pulmonary impact of patent ductus arteriosus shunting. *Pediatric Pulmonology* **57**, 1209-1213.
[195] Ceravolo R, Frosini D, Poletti M, Kiferle L, Pagni C, Mazzucchi S, Volterrani D, Bonuccelli U (2013) Mild affective symptoms in de novo Parkinson's disease patients: relationship with dopaminergic dysfunction. *European Journal of Neurology* **20**, 480-485.
[196] Chang CW, Yang SY, Yang CC, Chang CW, Wu YR (2020) Plasma and Serum Alpha-Synuclein as a Biomarker of Diagnosis in Patients With Parkinson's Disease. *Frontiers in Neurology* **10**, 7.
[197] Chen XQ, Niu JP, Peng RQ, Song YH, Xu N, Zhang YW (2019) The early diagnosis of Parkinson's disease through combined biomarkers. *Acta Neurologica Scandinavica* **140**, 268-273.
[198] Chen Y, Gao C, Sun Q, Pan H, Huang P, Ding J, Chen S (2017) MicroRNA-4639 Is a Regulator of DJ-1 Expression and a Potential Early Diagnostic Marker for Parkinson's Disease. *Front Aging Neurosci* **9**, 232.
[199] Chung SJ, Kim HR, Jung JH, Lee PH, Jeong Y, Sohn YH (2020) Identifying the Functional Brain Network of Motor Reserve in Early Parkinson's Disease. *Movement Disorders* **35**, 577-586.
[200] Concha-Marambio L, Weber S, Farris CM, Dakna M, Lang E, Wicke T, Ma Y, Starke M, Ebentheuer J, Sixel-Döring F, Muntean ML, Schade S, Trenkwalder C, Soto C, Mollenhauer B (2023) Accurate Detection of α-Synuclein Seeds in Cerebrospinal Fluid from Isolated Rapid Eye Movement Sleep Behavior Disorder and Patients with Parkinson's Disease in the DeNovo Parkinson (DeNoPa) Cohort. *Mov Disord*.

[201] Craig CE, Jenkinson NJ, Brittain JS, Grothe MJ, Rochester L, Silverdale M, Alho A, Alho EJL, Holmes PS, Ray NJ (2020) Pedunculopontine Nucleus Microstructure Predicts Postural and Gait Symptoms in Parkinson's Disease. *Movement Disorders* **35**, 1199-1207.

[202] Dayan E, Browner N (2017) Alterations in striato-thalamo-pallidal intrinsic functional connectivity as a prodrome of Parkinson's disease. *Neuroimage-Clinical* **16**, 313-318.

[203] Deng X, Saffari SE, Liu N, Xiao B, Allen JC, Ng SYE, Chia N, Tan YJ, Choi XY, Heng DL, Lo YL, Xu ZY, Tay KY, Au WL, Ng A, Tan EK, Tan LCS (2022) Biomarker characterization of clinical subtypes of Parkinson Disease. *Npj Parkinsons Disease* **8**, 8.

[204] Devos D, Labreuche J, Rascol O, Corvol JC, Duhamel A, Delannoy PG, Poewe W, Compta Y, Pavese N, Ruzicka E, Dusek P, Post B, Bloem BR, Berg D, Maetzler W, Otto M, Habert MO, Lehericy S, Ferreira J, Dodel R, Tranchant C, Eusebio A, Thobois S, Marques AR, Meissner WG, Ory-Magne F, Walter U, de Bie RMA, Gago M, Vilas D, Kulisevsky J, Januario C, Coelho MVS, Behnke S, Worth P, Seppi K, Ouk T, Potey C, Leclercq C, Viard R, Kuchcinski G, Lopes R, Pruvo JP, Pigny P, Garcon G, Simonin O, Carpentier J, Rolland AS, Nyholm D, Scherfler C, Mangin JF, Chupin M, Bordet R, Dexter DT, Fradette C, Spino M, Tricta F, Ayton S, Bush AI, Devedjian JC, Duce JA, Cabantchik I, Defebvre L, Deplanque D, Moreau C, Grp FIS (2022) Trial of Deferiprone in Parkinson's Disease. *New England Journal of Medicine* **387**, 2045-2055.

[205] Galet B, Ingallinesi M, Pegon J, Thi AD, Ravassard P, Biguet NF, Meloni R (2021) G-protein coupled receptor 88 knockdown in the associative striatum reduces psychiatric symptoms in a translational male rat model of Parkinson disease. *Journal of Psychiatry & Neuroscience* **46**, E44-E55.

[206] Hanna-Pladdy B, Pahwa R, Lyons KE (2021) Dopaminergic Basis of Spatial Deficits in Early Parkinson's Disease. *Cereb Cortex Commun* **2**, tgab042.

[207] Hassin-Baer S, Cohen OS, Israeli-Korn S, Yahalom G, Benizri S, Sand D, Issachar G, Geva AB, Shani-Hershkovich R, Peremen Z (2022) Identification of an early-stage Parkinson's disease neuromarker using event-related potentials, brain network analytics and machine-learning. *PLoS One* **17**, e0261947.

[208] Hertz E, Thornqvist M, Holmberg B, Machaczka M, Sidransky E, Svenningsson P (2019) First Clinicogenetic Description of Parkinson's Disease Related to GBA Mutation S107L. *Movement Disorders Clinical Practice* **6**, 254-258.

[209] Hinkle JT, Perepezko K, Mills KA, Mari Z, Butala A, Dawson TM, Pantelyat A, Rosenthal LS, Pontone GM (2018) Dopamine transporter availability reflects gastrointestinal dysautonomia in early Parkinson disease. *Parkinsonism & Related Disorders* **55**, 8-14.

[210] Inayat M, Bany-Mohammed F, Valencia A, Tay C, Jacinto J, Aranda JV, Beharry KD (2015) Antioxidants and Biomarkers of Oxidative Stress in Preterm Infants with Symptomatic Patent Ductus Arteriosus. *American Journal of Perinatology* **32**, 895-904.

[211] Jackson H, Anzures-Cabrera J, Taylor KI, Pagano G, Grp PIPS (2021) Hoehn and Yahr Stage and Striatal Dat-SPECT Uptake Are Predictors of Parkinson's Disease Motor Progression. *Frontiers in Neuroscience* **15**, 10.

[212] Jeancolas L, Petrovska-Delacretaz D, Mangone G, Benkelfat BE, Corvol JC, Vidailhet M, Lehericy S, Benali H (2021) X-Vectors: New Quantitative Biomarkers for Early Parkinson's Disease Detection From Speech. *Frontiers in Neuroinformatics* **15**, 18.

[213] Jiang QW, Wang C, Zhou Y, Hou MM, Wang X, Tang HD, Wu YW, Ma JF, Chen SD (2015) Plasma Epidermal Growth Factor Decreased in the Early Stage of Parkinson's Disease. *Aging and Disease* **6**, 168-173.

[214] Joutsa J, Johansson J, Seppanen M, Noponen T, Kaasinen V (2015) Dorsal-to-Ventral Shift in Midbrain Dopaminergic Projections and Increased Thalamic/Raphe Serotonergic Function in Early Parkinson Disease. *Journal of Nuclear Medicine* **56**, 1036-1041.

[215] Khosousi S, Hye A, Velayudhan L, Bloth B, Tsitsi P, Markaki I, Svenningsson P (2023) Complement system changes in blood in Parkinson's disease and progressive Supranuclear Palsy/Corticobasal Syndrome. *Parkinsonism & Related Disorders* **108**, 8.

[216] Kim R, Kim HJ, Kim A, Jang M, Kim A, Kim Y, Yoo D, Im JH, Choi JH, Jeon B (2018) Peripheral blood inflammatory markers in early Parkinson's disease. *Journal of Clinical Neuroscience* **58**, 30-33.

[217] Kim R, Park S, Yoo D, Jun JS, Jeon B (2021) Impact of the apolipoprotein E? 4 allele on early Parkinson ? s disease progression. *Parkinsonism & Related Disorders* **83**, 66-70.

[218] Kim R, Park S, Yoo D, Suh YJ, Jun JS, Jeon B (2021) Potential Sex-Specific Effects of Apolipoprotein E epsilon 4 on Cognitive Decline in Early Parkinson's Disease. *Journal of Parkinsons Disease* **11**, 497-505.

[219] Kim R, Shin JH, Park S, Kim HJ, Jeon B (2020) Longitudinal evolution of non-motor symptoms according to age at onset in early Parkinson's disease. *Journal of the Neurological Sciences* **418**, 7.

[220] Kim YJ, Park CW, Shin HW, Lee HS, Kim YJ, Yun MJ, Lee PH, Sohn YH, Jeong Y, Chung SJ (2022) Identifying the white matter structural network of motor reserve in early Parkinson's disease. *Parkinsonism & Related Disorders* **102**, 108-114.

[221] Kraus TFJ, Haider M, Spanner J, Steinmaurer M, Dietinger V, Kretzschmar HA (2017) Altered Long Noncoding RNA Expression Precedes the Course of Parkinson's Disease-a Preliminary Report. *Molecular Neurobiology* **54**, 2869-2877.

[222] Liu R, Umbach DM, Troster AI, Huang XM, Chen HL (2020) Non-motor symptoms and striatal dopamine transporter binding in early Parkinson's disease. *Parkinsonism & Related Disorders* **72**, 23-30.

[223] Mak E, Kouli A, Holland N, Nicastro N, Savulich G, Surendranathan A, Malpetti M, Manavaki R, Hong YT, Fryer TD, Aigbirhio F, Rowe JB, O'Brien JT, Williams-Gray CH [¹⁸F]-AV-1451 binding in the substantia nigra as a marker of neuromelanin in Lewy body diseases. *Brain Communications* **3**, fcab177.

[224] Masellis M, Collinson S, Freeman N, Tampakeras M, Levy J, Tchelet A, Eyal E, Berkovich E, Eliaz RE, Abler V, Grossman I, Fitzer-Attas C, Tiwari A, Hayden MR, Kennedy JL, Lang AE, Knight J (2016) Dopamine D2 receptor gene variants and response to rasagiline in early Parkinson's disease: a pharmacogenetic study. *Brain* **139**, 2050-2062.

[225] Mollenhauer B, Zimmermann J, Sixel-Döring F, Focke NK, Wicke T, Ebentheuer J, Schaumburg M, Lang E, Trautmann E, Zetterberg H, Taylor P, Friede T, Trenkwalder C (2016) Monitoring of 30 marker candidates in early Parkinson disease as progression markers. *Neurology* **87**, 168-177.

[226] Murtomaki K, Mertsalmi T, Jaakkola E, Makinen E, Levo R, Nojonen T, Eklund M, Nuuttila S, Lindholm K, Pekkonen E, Joutsa J, Noponen T, Ihalainen T, Kaasinen V, Scheperjans F

(2022) Gastrointestinal Symptoms and Dopamine Transporter Asymmetry in Early Parkinson's Disease. *Movement Disorders* **37**, 1284-1289.

[227] Niemann L, Lezius S, Maceski A, Leppert D, Englisch C, Schwedhelm E, Zeller T, Gerloff C, Kuhle J, Choe CU (2021) Serum neurofilament is associated with motor function, cognitive decline and subclinical cardiac damage in advanced Parkinson's disease (MARK-PD). *Parkinsonism & Related Disorders* **90**, 44-48.

[228] Nissen SK, Farmen K, Carstensen M, Schulte C, Goldeck D, Brockmann K, Romero-Ramos M (2022) Changes in CD163+, CD11b+, and CCR2+peripheral monocytes relate to Parkinson's disease and cognition. *Brain Behavior and Immunity* **101**, 182-193.

[229] Niu M, Li Y, Li G, Zhou L, Luo N, Yao M, Kang W, Liu J (2020) A longitudinal study on alpha-synuclein in plasma neuronal exosomes as a biomarker for Parkinson's disease development and progression. *European Journal of Neurology* **27**, 967-974.

[230] Oh YS, Kim JS, Yoo SW, Hwang EJ, Lyoo CH, Lee KS (2019) Striatal dopamine activity and myocardial I-123-metaiodobenzylguanidine uptake in early Parkinson's disease. *Parkinsonism & Related Disorders* **63**, 156-161.

[231] Park DG, Kim JW, An YS, Chang J, Yoon JH (2021) Plasma neurofilament light chain level and orthostatic hypotension in early Parkinson's disease. *Journal of Neural Transmission* **128**, 1853-1861.

[232] Pasquini J, Ceravolo R, Brooks DJ, Bonuccelli U, Pavese N (2020) Progressive loss of raphe nuclei serotonin transporter in early Parkinson's disease: A longitudinal (123)I-FP-CIT SPECT study. *Parkinsonism Relat Disord* **77**, 170-175.

[233] Paul KC, Binder AM, Horvath S, Kusters C, Yan Q, Rosario ID, Yu Y, Bronstein J, Ritz B (2021) Accelerated hematopoietic mitotic aging measured by DNA methylation, blood cell lineage, and Parkinson's disease. *Bmc Genomics* **22**, 10.

[234] Pavlou MAS, Colombo N, Fuertes-Alvarez S, Nicklas S, Cano LG, Marin MC, Goncalves J, Schwamborn JC (2017) Expression of the Parkinson's Disease-Associated Gene Alpha-Synuclein is Regulated by the Neuronal Cell Fate Determinant TRIM32. *Molecular Neurobiology* **54**, 4257-4270.

[235] Picillo M, Santangelo G, Erro R, Cozzolino A, Amboni M, Vitale C, Barone P, Pellecchia MT (2017) Association between dopaminergic dysfunction and anxiety in de novo Parkinson's disease. *Parkinsonism & Related Disorders* **37**, 106-110.

[236] Polychronis S, Niccohni F, Pagano G, Yousaf T, Politis M (2019) Speech difficulties in early de novo patients with Parkinson's disease. *Parkinsonism & Related Disorders* **64**, 256-261.

[237] Qamhawi Z, Towey D, Shah B, Pagano G, Seibyl J, Marek K, Borghammer P, Brooks DJ, Pavese N (2015) Clinical correlates of raphe serotonergic dysfunction in early Parkinson's disease. *Brain* **138**, 2964-2973.

[238] Rathnayake D, Chang T, Udagama P (2019) Selected serum cytokines and nitric oxide as potential multi-marker biosignature panels for Parkinson disease of varying durations: a case-control study. *Bmc Neurology* **19**, 10.

[239] Reams N, Anderson J, Perlman R, Li W, Walters S, Tideman S, Wang C, Simon K, Frigerio R, Maraganore DM (2018) Investigating ioflupane I(123) injection and single photon emission tomography as an imaging biomarker for long-term sequelae following mild traumatic brain injury. *Brain Inj* **32**, 105-112.

[240] Rutten S, van der Ven PM, Weintraub D, Pontone GM, Leentjens AFG, Berendse HW, van der Werf YD, van den Heuvel OA (2017) Predictors of anxiety in early-stage Parkinson's disease- Results from the first two years of a prospective cohort study. *Parkinsonism & Related Disorders* **43**, 49-55.

[241] Sampedro F, Marin-Lahoz J, Martinez-Horta S, Pagonabarraga J, Kulisevsky J (2019) Dopaminergic degeneration induces early posterior cortical thinning in Parkinson's disease. *Neurobiology of Disease* **124**, 29-35.

[242] Santangelo G, Vitale C, Picillo M, Cuoco S, Moccia M, Pezzella D, Erro R, Longo K, Vicidomini C, Pellecchia MT, Amboni M, Brunetti A, Salvatore M, Barone P, Pappata S (2015) Apathy and striatal dopamine transporter levels in de-novo, untreated Parkinson's disease patients. *Parkinsonism & Related Disorders* **21**, 489-493.

[243] Scheper M, Iyer A, Anink JJ, Mesarosova L, Mills JD, Aronica E (2023) Dysregulation of miR-543 in Parkinson's disease: Impact on the neuroprotective gene SIRT1. *Neuropathol Appl Neurobiol* **49**, e12864.

[244] Schwarz ST, Mougin O, Xing Y, Blazejewska A, Bajaj N, Auer DP, Gowland P (2018) Parkinson's disease related signal change in the nigrosomes 1-5 and the substantia nigra using T2*weighted 7T MRI. *Neuroimage-Clinical* **19**, 683-689.

[245] Sherbaf FG, Mohajer B, Ashraf-Ganjouei A, Zadeh MM, Javinani A, Moghaddam HS, Shandiz MS, Aarabi MH (2018) Serum Insulin-Like Growth Factor-1 in Parkinson's Disease; Study of Cerebrospinal Fluid Biomarkers and White Matter Microstructure. *Frontiers in Endocrinology* **9**, 11.

[246] Shu Y, Qian JJ, Wang CY (2020) Aberrant expression of microRNA-132-3p and microRNA-146a-5p in Parkinson's disease patients. *Open Life Sciences* **15**, 647-653.

[247] Siepel FJ, Bronnick KS, Booij J, Ravina BM, Lebedev AV, Pereira JB, Gruner R, Aarsland D (2014) Cognitive Executive Impairment and Dopaminergic Deficits in De Novo Parkinson's Disease. *Movement Disorders* **29**, 1802-1808.

[248] Singh AP, Ramana G, Bajaj T, Singh V, Dwivedi S, Behari M, Dey AB, Dey S (2019) Elevated Serum SIRT 2 May Differentiate Parkinson's Disease From Atypical Parkinsonian Syndromes. *Frontiers in Molecular Neuroscience* **12**, 8.

[249] Sun AG, Wang J, Shan YZ, Yu WJ, Li X, Cong CH, Wang X (2014) Identifying distinct candidate genes for early Parkinson's disease by analysis of gene expression in whole blood. *Neuroendocrinology Letters* **35**, 398-404.

[250] Tao MZ, Dou KX, Xie YJ, Hou BH, Xie AM, Parkinson's Progression Markers I (2022) The associations of cerebrospinal fluid biomarkers with cognition, and rapid eye movement sleep behavior disorder in early Parkinson's disease. *Frontiers in Neuroscience* **16**, 11.

[251] Uehara Y, Ueno SI, Amano-Takeshige H, Suzuki S, Imamichi Y, Fujimaki M, Ota N, Murase T, Inoue T, Saiki S, Hattori N (2021) Non-invasive diagnostic tool for Parkinson's disease by sebum RNA profile with machine learning. *Scientific Reports* **11**, 10.

[252] Vegas-Suarez S, Paredes-Rodriguez E, Aristieta A, Lafuente JV, Miguelez C, Ugedo L (2019) Dysfunction of serotonergic neurons in Parkinson's disease and dyskinesia. *Int Rev Neurobiol* **146**, 259-279.

[253] Voruz P, Constantin IM, Peron JA (2022) Biomarkers and non-motor symptoms as a function of motor symptom asymmetry in early Parkinson?s disease. *Neuropsychologia* **177**, 15.

[254] Xiong KP, Dai YP, Chen J, Xu JM, Wang Y, Feng P, You SJ, Liu CF (2018) Increased Serum Cystatin C in Early Parkinson's Disease With Objective Sleep Disturbances. *Chinese Medical Journal* **131**, 907-911.

[255] Yan JH, Hua P, Chen Y, Li LT, Yu CY, Yan L, Zhang H, He Y, Zheng H, Chen H, Zhang ZJ, Yao QH, Dong H, Liu WG (2020) Identification of microRNAs for the early diagnosis of Parkinson's disease and multiple system atrophy. *Journal of Integrative Neuroscience* **19**, 429-436.

[256] Yang N, Sang S, Peng T, Hu W, Wang J, Bai R, Lu H (2023) Impact of GBA variants on longitudinal freezing of gait progression in early Parkinson's disease. *J Neurol*.

[257] Yang XX, Li Z, Bai LP, Shen X, Wang F, Han XX, Zhang R, Li Z, Zhang JH, Dong MM, Wang YL, Cao TY, Zhao SJ, Chu CG, Liu C, Zhu XD (2022) Association of Plasma and Electroencephalography Markers With Motor Subtypes of Parkinson's Disease. *Frontiers in Aging Neuroscience* **14**, 13.

[258] Yong ACW, Tan YJ, Zhao Y, Lu ZH, Ng EYL, Ng SYE, Chia NSY, Choi XY, Heng DD, Neo S, Xu ZY, Tay KY, Au WL, Tan EK, Tan LCS, Ng ASL (2020) SNCA Rep1 microsatellite length influences non-motor symptoms in early Parkinson's disease. *Aging-Us* **12**, 20880-20887.

[259] Yuan YS, Zhou XJ, Tong Q, Zhang L, Zhang L, Qi ZQ, Ge S, Zhang KZ (2013) Change in Plasma Levels of Amino Acid Neurotransmitters and its Correlation with Clinical Heterogeneity in Early Parkinson's Disease Patients. *Cns Neuroscience & Therapeutics* **19**, 889-896.

[260] Zahi Q, Towey D, Shah B, Pagano G, Seiby J, Marek K, Borghammer P, Brooks DJ, Pavese N, Qamhawi Z, Seibyl J (2015) Clinical correlates of raphe serotonergic dysfunction in early Parkinson's disease. *Brain: A Journal of Neurology* **138**, 2964-2973.

[261] Zhang J, Mattison HA, Liu CQ, Ginghina C, Auinger P, McDermott MP, Stewart T, Kang UJ, Cain KC, Shi M, Parkinson Study Grp D (2013) Longitudinal assessment of tau and amyloid beta in cerebrospinal fluid of Parkinson disease. *Acta Neuropathologica* **126**, 671-682.

[262] Marrinan S, Bajaj N, Barker R, Ben-Shlomo Y, Emmanuel A, Foltynie T, Grosset D, Morris H, Williams N, Wood N, Burn D (2014) 131 GASTROPARESIS SYMPTOMS IN EARLY PARKINSON'S DISEASE. *Age & Ageing* **43**, i36-i36.

[263] Hou XY, Zhang Y, Wang YP, Wang XY, Zhao JH, Zhu XB, Su JB (2021) A Markerless 2D Video, Facial Feature Recognition-Based, Artificial Intelligence Model to Assist With Screening for Parkinson Disease: Development and Usability Study. *Journal of Medical Internet Research* **23**, 11.

[264] Taleb C, Likforman-Sulem L, Mokbel C (2021) in *16th IAPR International Conference on Document Analysis and Recognition (ICDAR)* Springer International Publishing Ag, Electr Network, pp. 397-413.

[265] Williamson JR, Telfer B, Mullany R, Friedl KE (2021) Detecting Parkinson's Disease from Wrist-Worn Accelerometry in the U.K. Biobank. *Sensors* **21**, 14.

[266] Zhang HB, Song C, Wang AS, Xu CH, Li DM, Xu WY, Assoc Comp M (2019) in *25th Annual International Conference on Mobile Computing and Networking (MobiCom)* Assoc Computing Machinery, Los Cabos, MEXICO.

[267] Amandola M, Sinha A, Amandola MJ, Leung HC (2022) Longitudinal corpus callosum microstructural decline in early-stage Parkinson's disease in association with akinetic-rigid symptom severity. *Npj Parkinsons Disease* **8**, 10.

[268] Araki N, Yamanaka Y, Poudel A, Fujinuma Y, Katagiri A, Kuwabara S, Asahina M (2021) Electrogastrography for diagnosis of early-stage Parkinson's disease. *Parkinsonism & Related Disorders* **86**, 61-66.

[269] Arnold C, Gehrig J, Gispert S, Seifried C, Kell CA (2014) Pathomechanisms and compensatory efforts related to Parkinsonian speech. *Neuroimage-Clinical* **4**, 82-97.

[270] Ashraf-Ganjouei A, Majd A, Javinani A, Aarabi MH (2018) Autonomic dysfunction and white matter microstructural changes in drug-naive patients with Parkinson's disease. *Peerj* **6**, 15.

[271] Bowman FD, Drake DF, Huddleston DE (2016) Multimodal Imaging Signatures of Parkinson's Disease. *Frontiers in Neuroscience* **10**, 11.

[272] Brandl SJ, Braune S (2019) Sensitivity and specificity of cardiac metaiodobenzylguanidine scintigraphy in the early diagnosis of Parkinson's disease. *Clinical Autonomic Research* **29**, 567-574.

[273] Buratachwatanasiri W, Chantadisai M, Onwanna J, Chongpison Y, Rakvongthai Y, Khamwan K (2021) Pharmacokinetic Modeling of F-18-FDOPA PET in the Human Brain for Parkinson's Disease. *Molecular Imaging and Radionuclide Therapy* **30**, 69-78.

[274] Caminiti SP, Presotto L, Baroncini D, Garibotto V, Moresco RM, Gianolli L, Volonte MA, Antonini A, Perani D (2017) Axonal damage and loss of connectivity in nigrostriatal and mesolimbic dopamine pathways in early Parkinson's disease. *Neuroimage-Clinical* **14**, 734-740.

[275] Cardaioli G, Ripandelli F, Paoletti FP, Nigro P, Simoni S, Brahimi E, Romoli M, Filidei M, Eusebi P, Calabresi P, Tambasco N (2019) Substantia nigra hyperechogenicity in essential tremor and Parkinson's disease: a longitudinal study. *European Journal of Neurology* **26**, 1370-1376.

[276] Chen NK, Chou YH, Sundman M, Hickey P, Kasoff WS, Bernstein A, Trouard TP, Lin T, Rapcsak SZ, Sherman SJ, Weingarten CP (2018) Alteration of Diffusion-Tensor Magnetic Resonance Imaging Measures in Brain Regions Involved in Early Stages of Parkinson's Disease. *Brain Connectivity* **8**, 343-349.

[277] Chu CG, Wang X, Cai LH, Zhang L, Wang J, Liu C, Zhu XD (2020) Spatiotemporal EEG microstate analysis in drug-free patients with Parkinson's disease. *Neuroimage-Clinical* **25**, 12.

[278] Chung SJ, Lee JJ, Ham JH, Lee PH, Sohn YH (2016) Apathy and striatal dopamine defects in non-demented patients with Parkinson's disease. *Parkinsonism & Related Disorders* **23**, 62-65.

[279] Dadar M, Fereshtehnejad SM, Zeighami Y, Dagher A, Postuma RB, Collins DL (2020) White Matter Hyperintensities Mediate Impact of Dysautonomia on Cognition in Parkinson's Disease. *Mov Disord Clin Pract* **7**, 639-647.

[280] Dan XJ, Hu Y, Sun JY, Gao LL, Zhou YT, Ma JH, Doyon JL, Wu T, Chan P (2021) Altered Cerebellar Resting-State Functional Connectivity in Early-Stage Parkinson's Disease Patients With Cognitive Impairment. *Frontiers in Neurology* **12**, 10.

[281] Fazio P, Svenningsson P, Cselenyi Z, Halldin C, Farde L, Varrone A (2018) Nigrostriatal dopamine transporter availability in early Parkinson's disease. *Movement Disorders* **33**, 592-599.

[282] Fu JF, Matarazzo M, McKenzie J, Neilson N, Vafai N, Dinelle K, Felicio AC, McKeown MJ, Stoessl AJ, Sossi V (2021) Serotonergic System Impacts Levodopa Response in Early Parkinson's and Future Risk of Dyskinesia. *Mov Disord* **36**, 389-397.
[283] Fu T, Klietz M, Nosel P, Wegner F, Schrader C, Hoglinger GU, Dadak M, Mahmoudi N, Lanfermann H, Ding XQ (2020) Brain Morphological Alterations Are Detected in Early-Stage Parkinson's Disease with MRI Morphometry. *Journal of Neuroimaging* **30**, 786-792.
[284] Fu YH, Zhou LC, Li HY, Hsiao JHT, Li BY, Tanglay O, Auwyang AD, Wang E, Feng JY, Kim WS, Liu J, Halliday GM (2022) Adaptive structural changes in the motor cortex and white matter in Parkinson's disease. *Acta Neuropathologica* **144**, 861-879.
[285] Gallagher CL, Bell B, Palotti M, Oh J, Christian BT, Okonkwo O, Sojkova J, Buyan-Dent L, Nickles RJ, Harding SJ, Stone CK, Johnson SC, Holden JE (2015) Anterior cingulate dopamine turnover and behavior change in Parkinson's disease. *Brain Imaging and Behavior* **9**, 821-827.
[286] Ghazi Sherbaf F, Rahmani F, Jooyandeh SM, Aarabi MH (2018) Microstructural changes in patients with Parkinson disease and REM sleep behavior disorder: depressive symptoms versus non-depressed. *Acta Neurologica Belgica* **118**, 415-421.
[287] Greenbaum L, Lorberboym M, Melamed E, Rigbi A, Barhum Y, Kohn Y, Khlebtovsky A, Lerer B, Djaldetti R (2013) Perspective: Identification of genetic variants associated with dopaminergic compensatory mechanisms in early Parkinson's disease. *Frontiers in Neuroscience* **7**, 9.
[288] Grimaldi S, El Mendili MM, Zaaraoui W, Ranjeva JP, Azulay JP, Eusebio A, Guye M (2021) Increased Sodium Concentration in Substantia Nigra in Early Parkinson's Disease: A Preliminary Study With Ultra-High Field (7T) MRI. *Frontiers in Neurology* **12**, 8.
[289] Guan JT, Zheng X, Lai L, Sun S, Geng Y, Zhang X, Zhou T, Wu HZ, Chen JQ, Yang ZX, Zheng XH, Wang JX, Chen W, Zhang YQ (2022) Proton Magnetic Resonance Spectroscopy for Diagnosis of Non-Motor Symptoms in Parkinson's Disease. *Front Neurol* **13**, 594711.
[290] Heuchan AM, Young D (2013) Early colour Doppler duct diameter and symptomatic patent ductus arteriosus in a cyclo-oxygenase inhibitor naive population. *Acta Paediatrica* **102**, 254-257.
[291] Hofmann A, Rosenbaum D, Int-Veen I, Ehlis AC, Brockmann K, Dehnen K, von Thaler AK, Berg D, Fallgatter AJ, Metzger FG (2021) Abnormally reduced frontal cortex activity during Trail-Making-Test in prodromal parkinson's disease-a fNIRS study. *Neurobiol Aging* **105**, 148-158.
[292] Hossein-Tehrani MR, Ghaedian T, Hooshmandi E, Kalhor L, Foroughi AA, Ostovan VR, Hossein-Tehrani MR (2020) Brain TRODAT-SPECT Versus MRI Morphometry in Distinguishing Early Mild Parkinson's Disease from Other Extrapyramidal Syndromes. *Journal of Neuroimaging* **30**, 683-689.
[293] Jiang YY, An HD, Xi Q, Yang WT, Xie HR, Li Y, Huang DY (2022) Diffusion Tensor Imaging Reveals Deep Brain Structure Changes in Early Parkinson's Disease Patients with Various Sleep Disorders. *Brain Sciences* **12**, 12.
[294] Klietz M, Elaman MH, Mahmoudi N, Nosel P, Ahlswede M, Wegner F, Hoglinger GU, Lanfermann H, Ding XQ (2021) Cerebral Microstructural Alterations in Patients With Early Parkinson's Disease Detected With Quantitative Magnetic Resonance Measurements. *Frontiers in Aging Neuroscience* **13**, 12.

[295] Kolmancic K, Perellón-Alfonso R, Pirtosek Z, Rothwell JC, Bhatia K, Kojovic M, Perellón-Alfonso R (2019) Sex differences in Parkinson's disease: A transcranial magnetic stimulation study. *Movement Disorders* **34**, 1873-1881.

[296] Kotagal V, Spino C, Bohnen NI, Koeppe R, Albin RL (2018) Serotonin, beta-amyloid, and cognition in Parkinson disease. *Annals of Neurology* **83**, 994-1002.

[297] Kumari S, Goyal V, Kumaran SS, Dwivedi SN, Srivastava A, Jagannathan NR (2020) Quantitative metabolomics of saliva using proton NMR spectroscopy in patients with Parkinson's disease and healthy controls. *Neurological Sciences* **41**, 1201-1210.

[298] Lauretani F, Ruffini L, Testa C, Salvi M, Scarlattei M, Baldari G, Zucchini I, Lorenzi B, Cattabiani C, Maggio M (2021) Cognitive and Behavior Deficits in Parkinson's Disease with Alteration of FDG-PET Irrespective of Age. *Geriatrics* **6**, 8.

[299] Li X, Xing Y, Martin-Bastida A, Piccini P, Auer DP (2018) Patterns of grey matter loss associated with motor subscores in early Parkinson's disease. *Neuroimage-Clinical* **17**, 498-504.

[300] Li XF, Xing Y, Schwarz ST, Auer DP (2017) Limbic Grey Matter Changes in Early Parkinson's Disease. *Human Brain Mapping* **38**, 3566-3578.

[301] Li YT, Huang XF, Ruan XH, Duan DN, Zhang YH, Yu SD, Chen AM, Wang ZX, Zou YJ, Xia MR, Wei XH (2022) Baseline cerebral structural morphology predict freezing of gait in early drug-naive Parkinson's disease. *Npj Parkinsons Disease* **8**, 9.

[302] Li YX, Huang PY, Guo T, Guan XJ, Gao T, Sheng WS, Zhou C, Wu JJ, Song Z, Xuan M, Gu QQ, Xu XJ, Yang YJ, Zhang MM (2020) Brain structural correlates of depressive symptoms in Parkinson's disease patients at different disease stage. *Psychiatry Research-Neuroimaging* **296**, 7.

[303] Lin SC, Lin KJ, Hsiao IT, Hsieh CJ, Lin WY, Lu CS, Wey SP, Yen TC, Kung MP, Weng YH (2014) In Vivo Detection of Monoaminergic Degeneration in Early Parkinson Disease by F-18-9-Fluoropropyl-(+)-Dihydrotetrabenzazine PET. *Journal of Nuclear Medicine* **55**, 73-79.

[304] Liu XX, Zhang S, Liu N, Sun AP, Zhang YS, Fan DS (2019) [Diagnostic value of tremor analysis in identifying the early Parkinson's syndrome]. *Beijing Da Xue Xue Bao Yi Xue Ban* **51**, 1096-1102.

[305] Lohle M, Wolz M, Beuthien-Baumann B, Oehme L, van den Hoff J, Kotzerke J, Reichmann H, Storch A (2020) Olfactory dysfunction correlates with putaminal dopamine turnover in early de novo Parkinson's disease. *Journal of Neural Transmission* **127**, 9-16.

[306] Louter M, Maetzler W, Prinzen J, van Lummel RC, Hobert M, Arends J, Bloem BR, Streffer J, Berg D, Overeem S, Liepelt-Scarfone I (2015) Accelerometer-based quantitative analysis of axial nocturnal movements differentiates patients with Parkinson's disease, but not high-risk individuals, from controls. *Journal of Neurology Neurosurgery and Psychiatry* **86**, 32-37.

[307] Maiti B, Perlmutter JS (2023) Imaging in Movement Disorders. *Continuum (Minneap Minn)* **29**, 194-218.

[308] Mak E, Kouli A, Holland N, Nicastro N, Savulich G, Surendranathan A, Malpetti M, Manavaki R, Hong YT, Fryer TD, Aigbirhio F, Rowe JB, O'Brien JT, Williams-Gray CH (2021) [(18)F]-AV-1451 binding in the substantia nigra as a marker of neuromelanin in Lewy body diseases. *Brain Commun* **3**, fcab177.

[309] Martens KAE, Matar E, Phillips JR, Shine JM, Grunstein RR, Halliday GM, Lewis SJG (2022) Narrow doorways alter brain connectivity and step patterns in isolated REM sleep behaviour disorder. *Neuroimage-Clinical* **33**, 9.

[310] Matt E, Foki T, Fischmeister F, Pirker W, Haubenberger D, Rath J, Lehrner J, Auff E, Beisteiner R (2017) Early dysfunctions of fronto-parietal praxis networks in Parkinson's disease. *Brain Imaging and Behavior* **11**, 512-525.

[311] Minett T, Su L, Mak E, Williams G, Firbank M, Lawson RA, Yarnall AJ, Duncan GW, Owen AM, Khoo TK, Brooks DJ, Rowe JB, Barker RA, Burn D, O'Brien JT (2018) Longitudinal diffusion tensor imaging changes in early Parkinson's disease: ICICLE-PD study. *Journal of Neurology* **265**, 1528-1539.

[312] Mishina M, Ishii K, Kimura Y, Suzuki M, Kitamura S, Ishibashi K, Sakata M, Oda K, Kobayashi S, Kimura K, Ishiwata K (2017) Adenosine A(1) receptors measured with C-11-MPDX PET in early Parkinson's disease. *Synapse* **71**, 9.

[313] Mishra VR, Sreenivasan KR, Yang ZS, Zhuang XW, Cordes D, Mari Z, Litvan I, Fernandez HH, Eidelberg D, Ritter A, Cummings JL, Walsh RR (2020) Unique white matter structural connectivity in early-stage drug-naive Parkinson disease. *Neurology* **94**, E774-E784.

[314] Oh YS, Kim JH, Yoo SW, Hwang EJ, Lyoo CH, Lee KS, Kim JS (2021) Neuropsychiatric symptoms and striatal monoamine availability in early Parkinson's disease without dementia. *Neurological Sciences* **42**, 711-718.

[315] Oh YS, Kim JS, Hwang EJ, Lyoo CH (2018) Striatal dopamine uptake and olfactory dysfunction in patients with early Parkinson's disease. *Parkinsonism & Related Disorders* **56**, 47-51.

[316] Oh YS, Yoo SW, Lyoo CH, Kim JS (2022) Decreased thalamic monoamine availability in drug-induced parkinsonism. *Scientific Reports* **12**, 7.

[317] Pagano G, Niccolini F, Wilson H, Yousaf T, Khan NL, Martino D, Plisson C, Gunn RN, Rabiner EA, Piccini P, Foltynie T, Politis M (2019) Comparison of phosphodiesterase 10A and dopamine transporter levels as markers of disease burden in early Parkinson's disease. *Movement Disorders* **34**, 1505-1515.

[318] Pelizzari L, Di Tella S, Lagana MM, Bergsland N, Rossetto F, Nemni R, Baglio F (2020) White matter alterations in early Parkinson's disease: role of motor symptom lateralization. *Neurological Sciences* **41**, 357-364.

[319] Pena-Nogales O, Ellmore TM, de Luis-Garcia R, Suescun J, Schiess MC, Giancardo L (2019) Longitudinal Connectomes as a Candidate Progression Marker for Prodromal Parkinson's Disease. *Frontiers in Neuroscience* **12**, 13.

[320] Pineda-Pardo JA, Sanchez-Ferro A, Monje MHG, Pavese N, Obeso JA (2022) Onset pattern of nigrostriatal denervation in early Parkinson's disease. *Brain* **145**, 1018-1028.

[321] Rahmani F, Jooyandeh SM, Shadmehr MH, Shojaie A, Noorizadeh F, Aarabi MH (2016) in *MICCAI Workshop on Computational Diffusion MRI* Springer-Verlag Berlin, Athens, GREECE, pp. 167-173.

[322] Sherbaf FG, Abadi YR, Zadeh MM, Ashraf-Ganjouei A, Moghaddam HS, Aarabi MH (2018) Microstructural Changes in Patients With Parkinson's Disease Comorbid With REM Sleep Behaviour Disorder and Depressive Symptoms. *Frontiers in Neurology* **9**, 12.

[323] Shin C, Lee S, Lee JY, Rhim JH, Park SW (2018) Non-Motor Symptom Burdens Are Not Associated with Iron Accumulation in Early Parkinson's Disease: a Quantitative Susceptibility Mapping Study. *Journal of Korean Medical Science* **33**, 9.

[324] Siebner TH, Fuglsang S, Madelung CF, Løkkegaard A, Bendtsen F, Hove JD, Damgaard M, Madsen JL, Siebner HR (2022) Gastric Emptying Is Not Delayed and Does Not Correlate With Attenuated Postprandial Blood Flow Increase in Medicated Patients With Early Parkinson's Disease. *Front Neurol* **13**, 828069.

[325] Smith KM, Xie SX, Weintraub D (2016) Incident impulse control disorder symptoms and dopamine transporter imaging in Parkinson disease. *Journal of Neurology Neurosurgery and Psychiatry* **87**, 864-870.

[326] Song IU, Chung YA, Oh JK, Chung SW (2014) An FP-CIT PET comparison of the difference in dopaminergic neuronal loss in subtypes of early Parkinson's disease. *Acta Radiologica* **55**, 366-371.

[327] Sreenivasan K, Mishra V, Bird C, Zhuang X, Yang Z, Cordes D, Walsh RR (2019) Altered functional network topology correlates with clinical measures in very early-stage, drug-naïve Parkinson's disease. *Parkinsonism Relat Disord* **62**, 3-9.

[328] Stosser S, Neugebauer H, Althaus K, Ludolph AC, Kassubek J, Schocke M (2016) Perihematomal Diffusion Restriction in Intracerebral Hemorrhage Depends on Hematoma Volume, But Does Not Predict Outcome. *Cerebrovascular Diseases* **42**, 280-287.

[329] Tan Y, Tan J, Luo C, Cui W, He H, Bin Y, Deng J, Tan R, Tan W, Liu T, Zeng N, Xiao R, Yao D, Wang X (2015) Altered Brain Activation in Early Drug-Naive Parkinson's Disease during Heat Pain Stimuli: An fMRI Study. *Parkinsons Dis* **2015**, 273019.

[330] Tang Y, Liu BL, Yang Y, Wang CM, Meng L, Tang BS, Guo JF (2018) Identifying mild-moderate Parkinson's disease using whole-brain functional connectivity. *Clinical Neurophysiology* **129**, 2507-2516.

[331] Tedroff J, Pedersen M, Aquilonius SM, Hartvig P, Jacobsson G, Langstrom B (1996) Levodopa-induced changes in synaptic dopamine in patients with Parkinson's disease as measured by C-11 raclopride displacement and PET. *Neurology* **46**, 1430-1436.

[332] Vogt BA (2019) Cingulate cortex in Parkinson's disease In *Cingulate Cortex*, Vogt BA, ed. Elsevier, Amsterdam, pp. 253-266.

[333] Wang JL, Yang QX, Sun XY, Vesek J, Mosher Z, Vasavada M, Chu J, Kanekar S, Shivkumar V, Venkiteswaran K, Subramanian T (2015) MRI evaluation of asymmetry of nigrostriatal damage in the early stage of early-onset Parkinson's disease. *Parkinsonism & Related Disorders* **21**, 590-596.

[334] Wang ZJ, Jia XQ, Chen HM, Feng T, Wang HL (2018) Abnormal Spontaneous Brain Activity in Early Parkinson's Disease With Mild Cognitive Impairment: A Resting-State fMRI Study. *Frontiers in Physiology* **9**, 10.

[335] Wen MC, Ng SY, Heng HS, Chao YX, Chan LL, Tan EK, Tan LC (2016) Neural substrates of excessive daytime sleepiness in early drug naïve Parkinson's disease: A resting state functional MRI study. *Parkinsonism Relat Disord* **24**, 63-68.

[336] Wieler M, Gee M, Martin WRW (2015) Longitudinal midbrain changes in early Parkinson's disease: iron content estimated from R2*/MRI. *Parkinsonism & Related Disorders* **21**, 179-183.

[337] Wu G, Shen YJ, Huang MH, Xing Z, Liu Y, Chen J (2016) Proton MR Spectroscopy for Monitoring Pathologic Changes in the Substantia Nigra and Globus Pallidus in Parkinson Disease. *American Journal of Roentgenology* **206**, 385-389.

[338] Wu T, Hou YA, Hallett M, Zhang JR, Chan P (2015) Lateralization of Brain Activity Pattern During Unilateral Movement in Parkinson's Disease. *Human Brain Mapping* **36**, 1878-1891.

[339] Xiao YM, Peters TM, Khan AR (2021) Characterizing white matter alterations subject to clinical laterality in drug-naive de novo Parkinson's disease. *Human Brain Mapping* **42**, 4465-4477.

[340] Yang J, Archer DB, Burciu RG, Muller M, Roy A, Ofori E, Bohnen NI, Albin RL, Vaillancourt DE (2019) Multimodal dopaminergic and free-water imaging in Parkinson's disease. *Parkinsonism & Related Disorders* **62**, 10-15.

[341] Yang Y, Tang BS, Weng L, Li N, Shen L, Wang J, Zuo CT, Yan XX, Xia K, Guo JF (2015) Genetic Identification Is Critical for the Diagnosis of Parkinsonism: A Chinese Pedigree with Early Onset of Parkinsonism. *Plos One* **10**, 12.

[342] Yi GS, Wang YB, Wang LF, Chu CG, Wang J, Shen X, Han XX, Li Z, Bai LP, Li Z, Zhang R, Wang YL, Zhu XD, Liu C (2023) Capturing the Abnormal Brain Network Activity in Early Parkinsons Disease With Mild Cognitive Impairment Based on Dynamic Functional Connectivity. *Ieee Transactions on Neural Systems and Rehabilitation Engineering* **31**, 1238-1247.

[343] Yoo SW, Oh YS, Hwang EJ, Ryu DW, Lee KS, Lyoo CH, Kim JS (2019) "Depressed" caudate and ventral striatum dopamine transporter availability in de novo Depressed Parkinson's disease. *Neurobiology of Disease* **132**, 7.

[344] Youn J, Won JH, Kim M, Kwon J, Moon SH, Kim M, Ahn JH, Mun JK, Park H, Cho JW (2023) Extra-Basal Ganglia Brain Structures Are Related to Motor Reserve in Parkinson's Disease. *Journal of Parkinsons Disease* **13**, 39-48.

[345] Zeng WQ, Fan WL, Kong XC, Liu XM, Liu L, Cao ZQ, Zhang XQ, Yang XM, Cheng C, Wu Y, Xu Y, Cao XB, Xu Y (2022) Altered Intra- and Inter-Network Connectivity in Drug-Naive Patients With Early Parkinson's Disease. *Frontiers in Aging Neuroscience* **14**, 12.

[346] Zhang GH, Zhang CG, Wang YK, Wang LJ, Zhang YH, Xie HQ, Lu JC, Nie K (2019) Is hyperhomocysteinemia associated with the structural changes of the substantia nigra in Parkinson's disease? A two-year follow-up study. *Parkinsonism & Related Disorders* **60**, 46-50.

[347] Zhao Y, Zheng X, Wang Q, Xu J, Xu X, Zhang M (2014) Altered activation in visual cortex: unusual functional magnetic resonance imaging finding in early Parkinson's disease. *J Int Med Res* **42**, 503-515.

[348] Huang LQ, Ye XF, Yang MJ, Pan L, Zheng SH (2023) MNC-Net: Multi-task graph structure learning based on node clustering for early Parkinson's disease diagnosis. *Computers in Biology and Medicine* **152**, 12.

[349] Kaixin D, Jiangnan M, Xue Z, Wanda S, Mingzhu T, Anmu X (2022) Multi-predictor modeling for predicting early Parkinson's disease and non-motor symptoms progression. *Frontiers in Aging Neuroscience* **14**, 1-12.

[350] Sauerbier A, Jenner P, Todorova A, Chaudhuri KR (2016) Non motor subtypes and Parkinson's disease. *Parkinsonism & Related Disorders* **22**, S41-S46.

[351] Severson KA, Chahine LM, Smolensky LA, Dhuliawala M, Frasier M, Ng K, Ghosh S, Hu JY (2021) Discovery of Parkinson's disease states and disease progression modelling: a longitudinal data study using machine learning. *Lancet Digital Health* **3**, E555-E564.

[352] Simuni T, Long JD, Caspell-Garcia C, Coffey CS, Lasch S, Tanner CM, Jennings D, Kieburtz KD, Marek K, Investigators P (2016) Predictors of time to initiation of symptomatic therapy in early Parkinson's disease. *Annals of Clinical and Translational Neurology* **3**, 482-494.

[353] Wang C, Peng L, Hou ZG, Li YF, Tan Y, Hao HL (2022) A Hierarchical Architecture for Multisymptom Assessment of Early Parkinson's Disease via Wearable Sensors. *Ieee Transactions on Cognitive and Developmental Systems* **14**, 1553-1563.

[354] Bejr-kasem H, Sampedro F, Marin-Lahoz J, Martinez-Horta S, Pagonabarraga J, Kulisevsky J (2021) Minor hallucinations reflect early gray matter loss and predict subjective cognitive decline in Parkinson's disease. *European Journal of Neurology* **28**, 438-447.

[355] Hiorth YH, Pedersen KF, Dalen I, Tysnes OB, Alves G (2019) Orthostatic hypotension in Parkinson disease A 7-year prospective population-based study. *Neurology* **93**, E1526-E1534.

[356] Kudrevatykh A, Senkevich K, Miliukhina I (2020) Postural instability and neuropsychiatric disturbance in the overlapping phenotype of essential tremor and Parkinson's Disease. *Neurophysiologie Clinique-Clinical Neurophysiology* **50**, 489-494.

[357] Liepelt-Scarfone I, Brandle B, Yilmaz R, Gauss K, Schaeffer E, Timmers M, Wurster I, Brockmann K, Maetzler W, Van Nueten L, Streffer JR, Berg D (2017) Progression of prodromal motor and non-motor symptoms in the premotor phase study-2-year follow-up data. *European Journal of Neurology* **24**, 1369-1374.

[358] Liepelt-Scarfone I, Gauss K, Maetzler W, Muller K, Bormann C, Berger MF, Timmers M, Streffer J, Berg D (2013) Evaluation of Progression Markers in the Premotor Phase of Parkinson's Disease: The Progression Markers in the Premotor Phase Study. *Neuroepidemiology* **41**, 174-182.

[359] Martinez-Martin P, Skorvanek M, Henriksen T, Lindvall S, Domingos J, Alobaidi A, Kandukuri PL, Chaudhari VS, Patel AB, Parra JC, Pike J, Antonini A (2023) Impact of advanced Parkinson's disease on caregivers: an international real-world study. *J Neurol* **270**, 2162-2173.

[360] Masarova L, Drotar P, Mekyska J, Smekal Z, Rektorova I (2014) Assessing Handwriting in Patients with Parkinson's Disease. *Ceska a Slovenska Neurologie a Neurochirurgie* **77**, 456-462.

[361] Rascol O, Fabbri M, Poewe W (2021) Amantadine in the treatment of Parkinson's disease and other movement disorders. *Lancet Neurology* **20**, 1048-1056.

[362] Rochester L, Galna B, Lord S, Burn D (2014) THE NATURE OF DUAL-TASK INTERFERENCE DURING GAIT IN INCIDENT PARKINSON'S DISEASE. *Neuroscience* **265**, 83-94.

[363] Roy HA, Nettleton J, Blain C, Dalton C, Farhan B, Fernandes A, Georgopoulos P, Klepsch S, Lavelle J, Martinelli E, Panicker JN, Radoja I, Rapidi CA, Silva RPE, Tudor K, Wagg AS, Drake MJ (2020) Assessment of patients with lower urinary tract symptoms where an undiagnosed neurological disease is suspected: A report from an International Continence Society consensus working group. *Neurourology and Urodynamics* **39**, 2535-2543.

[364] Simonet C, Bestwick J, Jitlal M, Waters S, Ben-Joseph A, Marshall CR, Dobson R, Marrium S, Robson J, Jacobs BM, Belete D, Lees AJ, Giovannoni G, Cuzick J, Schrag A, Noyce AJ (2022) Assessment of Risk Factors and Early Presentations of Parkinson Disease in Primary Care in a Diverse UK Population. *Jama Neurology* **79**, 359-369.

[365] Studer V, Maestri R, Clerici I, Spina L, Zivi I, Ferrazzoli D, Frazzitta G (2017) Treadmill Training with Cues and Feedback Improves Gait in People with More Advanced Parkinson's Disease. *Journal of Parkinsons Disease* **7**, 729-739.

[366] Sumi Y, Ubara A, Ozeki Y, Kadotani H (2022) Minor hallucinations in isolated rapid eye movement sleep behavior disorder indicative of early phenoconversion: A preliminary study. *Acta Neurologica Scandinavica* **145**, 348-359.

[367] Yang HJ, Kim YE, Yun JY, Ehm G, Kim HJ, Jeon BS (2014) Comparison of sleep and other non-motor symptoms between SWEDDs patients and de novo Parkinson's disease patients. *Parkinsonism & Related Disorders* **20**, 1419-1422.

[368] Yildiz EP, Yesil G, Ozkan MU, Bektas G, Caliskan M, Ozmen M (2017) A novel EPM2A mutation in a patient with Lafora disease presenting with early parkinsonism symptoms in childhood. *Seizure* **51**, 77-79.

[369] 张淼, 陈曦, 张惠红, 蔡莉, 周玉颖 (2017) 帕金森叠加综合征神经精神症状及PET 影像学特征. *Chinese Journal of Contemporary Neurology & Neurosurgery* **17**, 39-45.

[370] 邹慧莉, 赵显超, 江应聪, 雷革胜, 杨伟毅, 宿长军 (2017) 早期帕金森病患者快速眼动睡眠期行为障碍研究. *Chinese Journal of Contemporary Neurology & Neurosurgery* **17**, 736-740.

[371] (2018) Breath test offers hope of early Parkinson’s diagnosis. *British Journal of Healthcare Assistants* **12**, 466-466.

[372] Afentou N, Jarl J, Gerdtham UG, Saha S (2019) Economic Evaluation of Interventions in Parkinson's Disease: A Systematic Literature Review. *Movement Disorders Clinical Practice* **6**, 282-290.

[373] Afshin-Majd S, Khalili M, Roghani M, Mehranmehr N, Baluchnejadmojarad T (2015) Carnosine Exerts Neuroprotective Effect Against 6-Hydroxydopamine Toxicity in Hemiparkinsonian Rat. *Molecular Neurobiology* **51**, 1064-1070.

[374] Al-Qassabi A, Pelletier A, Fereshtehnejad SM, Postuma RB (2018) Autonomic Sweat Responses in REM Sleep Behavior Disorder and Parkinsonism. *Journal of Parkinsons Disease* **8**, 463-468.

[375] Belousov D, Afanasieva E (2015) Budget Impact Analysis of Pramipexole Extended Release Monotherapy In Early Parkinson'S Disease. *Value in Health* **18**, A752-A753.

[376] Bottcher T, Rolfs A, Meyer B, Grossmann A, Berg D, Kropp P, Benecke R, Walter U (2013) Clinical, genetic, and brain sonographic features related to Parkinson's disease in Gaucher disease. *Journal of Neurology* **260**, 2523-2531.

[377] Boura E, Stamelou M, Vadasz D, Ries V, Unger MM, Kägi G, Oertel WH, Möller JC, Mylius V (2017) Is increased spinal nociception another hallmark for Parkinson's disease? *J Neurol* **264**, 570-575.

[378] Camalier CR, Konrad PE, Gill CE, Kao C, Remple MR, Nasr HM, Davis TL, Hedera P, Phibbs FT, Molinari AL, Neimat JS, Charles D (2014) Methods for surgical targeting of the STN in early-stage Parkinson's disease. *Frontiers in Neurology* **5**, 6.

[379] Chandler C, Folse H, Gal P, Chavan A, Proskorovsky I, Franco-Villalobos C, Yang Y, Ward A (2021) Modeling long-term health and economic implications of new treatment strategies for Parkinson's disease: an individual patient simulation study. *J Mark Access Health Policy* **9**, 1922163.

[380] Chen Y, Xue NJ, Fang Y, Jin CY, Li YL, Tian J, Yan YP, Yin XZ, Zhang BR, Pu JL (2022) Association of Concurrent Olfactory Dysfunction and Probable Rapid Eye Movement Sleep Behavior Disorder with Early Parkinson's Disease Progression. *Movement Disorders Clinical Practice* **9**, 909-919.

[381] Choi SM, Cho SH, Kang KW, Kim JM, Kim BC (2021) Family history of hand tremor in patients with early Parkinson's disease. *Journal of Clinical Neuroscience* **90**, 161-164.

[382] Chu YP, Morfini GA, Kordower JH (2016) Alterations in Activity-Dependent Neuroprotective Protein in Sporadic and Experimental Parkinson's Disease. *Journal of Parkinsons Disease* **6**, 77-97.

[383] De Natale ER, Ginatempo F, Paulus KS, Manca A, Mercante B, Pes GM, Agnetti V, Tolu E, Deriu F (2015) Paired neurophysiological and clinical study of the brainstem at different stages of Parkinson's Disease. *Clinical Neurophysiology* **126**, 1871-1878.

[384] Devi B, Srivastava S, Verma VK (2021) in *4th International Conference on Information Systems and Management Science (ISMS)* Springer International Publishing Ag, Msida, MALTA, pp. 540-557.

[385] Doppler K, Jentschke HM, Schulmeyer L, Vadasz D, Janzen A, Luster M, Hoffken H, Mayer G, Brumberg J, Booij J, Musacchio T, Klebe S, Sittig-Wiegand E, Volkmann J, Sommer C, Oertel WH (2017) Dermal phospho-alpha-synuclein deposits confirm REM sleep behaviour disorder as prodromal Parkinson's disease. *Acta Neuropathologica* **133**, 535-545.

[386] Dou KX, Ma JN, Zhang X, Shi WD, Tao MZ, Xie AM, Parkinson's Progression Markers I (2022) Multi-predictor modeling for predicting early Parkinson's disease and non-motor symptoms progression. *Frontiers in Aging Neuroscience* **14**, 12.

[387] Fantini ML, Fedler J, Pereira B, Weintraub D, Marques AR, Durif F (2020) Is Rapid Eye Movement Sleep Behavior Disorder a Risk Factor for Impulse Control Disorder in Parkinson Disease? *Annals of Neurology* **88**, 759-770.

[388] Fundament T, Eldridge PR, Green AL, Whone AL, Taylor RS, Williams AC, Schuepbach WM (2016) Deep Brain Stimulation for Parkinson's Disease with Early Motor Complications: A UK Cost-Effectiveness Analysis. *PLoS One* **11**, e0159340.

[389] Gafoor SHA, Theagarajan P (2022) Intelligent approach of score-based artificial fish swarm algorithm (SAFSA) for Parkinson's disease diagnosis. *International Journal of Intelligent Computing and Cybernetics* **15**, 540-561.

[390] Galna B, Lord S, Rochester L (2013) Is gait variability reliable in older adults and Parkinson's disease? Towards an optimal testing protocol. *Gait & Posture* **37**, 580-585.

[391] Gjerde KV, Muller B, Skeie GO, Assmus J, Alves G, Tysnes OB (2018) Hyposmia in a simple smell test is associated with accelerated cognitive decline in early Parkinson's disease. *Acta Neurologica Scandinavica* **138**, 508-514.

[392] Guo S, Li L, Dai Y, Tang Q, Chen Y, Li S, Chen J, Mao C, Li J, Liu C (2015) [Correlations between olfactory and cognitive functions in early stage Parkinson's disease]. *Zhonghua Yi Xue Za Zhi* **95**, 489-492.

[393] Hessam S, Vahdat S, Asl IM, Kazemipoor M, Aghaei A, Shamshirband S, Rabczuk T (2019) Parkinson's Disease Detection Using Biogeography-Based Optimization. *Cmc-Computers Materials & Continua* **61**, 11-26.

[394] Heusinkveld L, Hacker M, Turchan M, Bollig M, Tamargo C, Fisher W, McLaughlin L, Martig A, Charles D (2017) Patient Perspectives on Deep Brain Stimulation Clinical Research in Early Stage Parkinson's Disease. *Journal of Parkinsons Disease* **7**, 89-94.

[395] Hong CT, Chan L, Wu D, Chen WT, Chien LN (2019) Association Between Parkinson's Disease and Atrial Fibrillation: A Population-Based Study. *Frontiers in Neurology* **10**, 6.

[396] Iakovakis D, Hadjidimitriou S, Charisis V, Bostanjopoulou S, Katsarou Z, Klingelhoefer L, Mayer S, Reichmann H, Dias SB, Diniz JA, Trivedi D, Chaudhuri RK, Hadjileontiadis LJ, Ieee (2019) in *41st Annual International Conference of the IEEE Engineering in Medicine and Biology Society (EMBC)* Ieee, Berlin, GERMANY, pp. 3535-3538.

[397] Iakovakis D, Mastoras RE, Hadjidimitriou S, Charisis V, Bostanjopoulou S, Katsarou Z, Klingelhoefer L, Reichmann H, Trivedi D, Chaudhuri RK, Hadjileontiadis LJ, Ieee (2020) in *42nd Annual International Conference of the IEEE-Engineering-in-Medicine-and-Biology-Society (EMBC)* Ieee, Montreal, CANADA, pp. 4326-4329.

[398] Ibrahim N, Kusmirek J, Struck AF, Floberg JM, Perlman SB, Gallagher C, Hall LT (2016) The sensitivity and specificity of F-DOPA PET in a movement disorder clinic. *American Journal of Nuclear Medicine and Molecular Imaging* **6**, 102-109.

[399] Jurcau A, Nunkoo VS (2021) Clinical Markers May Identify Patients at Risk for Early Parkinson's Disease Dementia: A Prospective Study. *American Journal of Alzheimers Disease and Other Dementias* **36**, 9.

[400] Kanavou S, Pitz V, Lawton MA, Malek N, Grosset KA, Morris HR, Ben-Shlomo Y, Grosset DG (2021) Comparison between four published definitions of hyposmia in Parkinson's disease. *Brain and Behavior* **11**, e2258.

[401] Kang KW, Choi SM, Kim BC (2022) Gender differences in motor and non-motor symptoms in early Parkinson disease. *Medicine* **101**, 5.

[402] Kelly MJ, Lawton MA, Baig F, Ruffmann C, Barber TR, Lo C, Klein JC, Ben-Shlomo Y, Hu MT (2019) Predictors of motor complications in early Parkinson's disease: A prospective cohort study. *Movement Disorders* **34**, 1174-1183.

[403] Keranen T, Virta LJ (2016) Association of guidelines and clinical practice in early Parkinson's disease. *European Geriatric Medicine* **7**, 131-134.

[404] Khaskhoussy R, Ben Ayed Y (2022) in *18th International Conference on Advanced Data Mining and Applications (ADMA)* Springer International Publishing Ag, Brisbane, AUSTRALIA, pp. 15-26.

[405] Kim R, Choi S, Byun K, Kang N, Suh YJ, Jun JS, Jeon B (2023) Association of Early Weight Change With Cognitive Decline in Patients With Parkinson Disease. *Neurology* **100**, E232-E241.

[406] Kim R, Kim HJ, Shin JH, Lee CY, Jeon SH, Jeon B (2022) Serum Inflammatory Markers and Progression of Nonmotor Symptoms in Early Parkinson's Disease. *Movement Disorders* **37**, 1535-1541.

[407] Kwon KY, Lee HM, Kang SH, Pyo SJ, Kim HJ, Koh SB (2017) Recuperation of slow walking in de novo Parkinson's disease is more closely associated with increased cadence, rather than with expanded stride length. *Gait & Posture* **58**, 1-6.

[408] Lamba R, Gulati T, Alharbi HF, Jain A A hybrid system for Parkinson's disease diagnosis using machine learning techniques. *International Journal of Speech Technology*, 11.

[409] Lamba R, Gulati T, Jain A (2022) An Intelligent System for Parkinson's Diagnosis Using Hybrid Feature Selection Approach. *International Journal of Software Innovation* **10**, 13.

[410] Madanchi A, Taghavi-Shahri F, Taghavi-Shahri SM, Tabar MRR (2020) Scaling behavior in measured keystroke time series from patients with Parkinson's disease. *European Physical Journal B* **93**, 8.

[411] Mak E, Kouli A, Holland N, Nicastro N, Savulich G, Surendranathan A, Malpetti M, Manavaki R, Hong YT, Fryer TD, Aigbirhio F, Rowe JB, O'Brien JT, Williams-Gray CH (2021) F-18-AV-1451 binding in the substantia nigra as a marker of neuromelanin in Lewy body diseases. *Brain Communications* **3**, 9.

[412] Maple-Grodem J, Paul KC, Dalen I, Ngo KJ, Wong D, Macleod AD, Counsell CE, Backstrom D, Forsgren L, Tysnes OB, Kusters CDJ, Fogel BL, Bronstein JM, Ritz B, Alves G (2021) Lack of Association Between GBA Mutations and Motor Complications in European and American Parkinson's Disease Cohorts. *Journal of Parkinsons Disease* **11**, 1569-1578.

[413] Martinez-Horta S, Bejr-Kasem H, Horta-Barba A, Pascual-Sedano B, Santos-Garcia D, De Deus-Fonticoba T, Jesus S, Aguilar M, Planellas L, Garcia-Caldentey J, Caballol N, Vives-Pastor B, Hernandez-Vara J, Cabo-Lopez I, Lopez-Manzanares L, Gonzalez-Aramburu I, Avila-Rivera MA, Catalan MJ, Lopez-Diaz LM, Puente V, Garcia-Moreno JM, Borrue C, Solano-Vila B, Alvarez-Sauco M, Vela L, Escalante S, Cubo E, Carrillo-Padilla F, Martinez-Castrillo JC, Sanchez-Alonso P, Alonso-Losada MG, Lopez-Ariztegui N, Gaston I, Blazquez-Estrada M, Seijo-Martinez M, Ruiz-Martinez J, Valero-Merino C, Kurtis M, De Fabregues-Boixar O, Gonzalez-Ardura J, Prieto-Jurczynska C, Martinez-Martin P, Mir P, Kulisevsky J, Study C (2021) Identifying comorbidities and lifestyle factors contributing to the cognitive profile of early Parkinson's disease. *Bmc Neurology* **21**, 10.

[414] Muller-Oehring EM, Sullivan EV, Pfefferbaum A, Huang NC, Poston KL, Bronte-Stewart HM, Schulte T (2015) Task-rest modulation of basal ganglia connectivity in mild to moderate Parkinson's disease. *Brain Imaging and Behavior* **9**, 619-638.

[415] Netser R, Demmin DL, Dobkin R, Goldstein A, Roche M, Zernik AN, Silverstein SM (2021) Flash Electroretinography Parameters and Parkinson's Disease. *Journal of Parkinsons Disease* **11**, 251-259.

[416] Nishida N, Yoshida K, Hata Y (2017) Sudden unexpected death in early Parkinson's disease: neurogenic or cardiac death? *Cardiovascular Pathology* **30**, 19-22.

[417] Nolano M, Caporaso G, Manganelli F, Stancanelli A, Borreca I, Mozzillo S, Tozza S, Dubbioso R, Iodice R, Vitale F, Koay S, Vichayanrat E, da Silva FV, Santoro L, Iodice V, Provitera V (2022) Phosphorylated alpha-Synuclein Deposits in Cutaneous Nerves of Early Parkinsonism. *Journal of Parkinsons Disease* **12**, 2453-2468.

[418] Oung QW, Muthusamy H, Basah SN, Lee H, Vijean V (2018) Empirical Wavelet Transform Based Features for Classification of Parkinson's Disease Severity. *Journal of Medical Systems* **42**, 17.

[419] Pellicano C, Benincasa D, Fanciulli A, Latino P, Giovannelli M, Pontieri FE (2013) The impact of extended release dopamine agonists on prescribing patterns for therapy of early Parkinson's disease: an observational study. *European Journal of Medical Research* **18**, 6.
[420] Pradhan S, Kelly VE (2019) Quantifying physical activity in early Parkinson disease using a commercial activity monitor. *Parkinsonism & Related Disorders* **66**, 171-175.
[421] Prakash N, Caspell-Garcia C, Coffey C, Siderowf A, Tanner CM, Kieburtz K, Mollenhauer B, Galasko D, Merchant K, Foroud T, Chahine LM, Weintraub D, Casaceli C, Dorsey R, Wilson R, Herzog M, Daegele N, Arnedo V, Frasier M, Sherer T (2019) Feasibility and safety of lumbar puncture in the Parkinson's disease research participants: Parkinson's Progression Marker Initiative (PPMI). *Parkinsonism & Related Disorders* **62**, 201-209.
[422] Qin XL, Zhang QS, Sun L, Hao MW, Hu ZT (2015) Lower Serum Bilirubin and Uric Acid Concentrations in Patients with Parkinson's Disease in China. *Cell Biochemistry and Biophysics* **72**, 49-56.
[423] Schaeffer E, Rogge A, Nieding K, Helmker V, Letsch C, Hauptmann B, Berg D (2020) Patients' views on the ethical challenges of early Parkinson disease detection. *Neurology* **94**, E2037-E2044.
[424] Schapira AHV, Barone P, Hauser RA, Mizuno Y, Rascol O, Busse M, Debieuvre C, Fraessdorf M, Poewe W, Pramipexole ERSG (2013) Patient-reported convenience of once-daily versus three-times-daily dosing during long-term studies of pramipexole in early and advanced Parkinson's disease. *European Journal of Neurology* **20**, 50-56.
[425] Schroeder U, Behnke S, Buchholz HG, Fassbender K, Schreckenberger M, Berg D (2013) Substantia nigra hyperechogenicity in healthy controls: a (18)Fluoro Dopa-PET follow-up study. *Journal of Neurology* **260**, 1907-1911.
[426] Siciliano M, De Micco R, Giordano A, Di Nardo F, Russo A, Caiazzo G, De Mase A, Cirillo M, Tedeschi G, Trojano L, Tessitore A (2020) Supplementary motor area functional connectivity in "drug-naïve" Parkinson's disease patients with fatigue. *J Neural Transm (Vienna)* **127**, 1133-1142.
[427] Siepmann T, Arndt M, Sedghi A, Szatmári S, Jr., Horváth T, Takáts A, Bereczki D, Moskopp ML, Buchmann S, Skowronek C, Zago W, Woranush W, Lapusca R, Weidemann ML, Gibbons CH, Freeman R, Reichmann H, Puetz V, Barlinn K, Pintér A, Illigens BM (2023) Two-Year observational study of autonomic skin function in patients with Parkinson's disease compared to healthy individuals. *Eur J Neurol*.
[428] Siepmann T, Frenz E, Penzlin AI, Goelz S, Zago W, Friehs I, Kubasch ML, Wienecke M, Lohle M, Schrempf W, Barlinn K, Siegert J, Storch A, Reichmann H, Illigens BMW (2016) Pilomotor function is impaired in patients with Parkinson's disease: A study of the adrenergic axon-reflex response and autonomic functions. *Parkinsonism & Related Disorders* **31**, 129-134.
[429] Sun Y, Ng ML, Lian CY, Wang L, Yang F, Yan N, Ieee (2018) in *11th International Symposium on Chinese Spoken Language Processing (ISCSLP)* Ieee, Academia Sinica, Taipei, TAIWAN, pp. 354-358.
[430] Terao Y, Tokushige S, Inomata-Terada S, Fukuda H, Yugeta A, Ugawa Y (2019) Differentiating early Parkinson's disease and multiple system atrophy with parkinsonism by saccade velocity profiles. *Clinical Neurophysiology* **130**, 2203-2215.

[431] Thompson MR, Stone RF, Ochs VD, Litvan I (2013) Primary Health Care Providers' Knowledge Gaps on Parkinson's Disease. *Educational Gerontology* **39**, 856-862.

[432] Tinazzi M, Abbruzzese G, Antonini A, Ceravolo R, Fabbrini G, Lessi P, Barone P, Grp RS (2013) Reasons driving treatment modification in Parkinson's disease: Results from the cross-sectional phase of the REASON study. *Parkinsonism & Related Disorders* **19**, 1130-1135.

[433] Umehara T, Nakahara A, Matsuno H, Toyoda C, Oka H (2017) Body weight and dysautonomia in early Parkinson's disease. *Acta Neurologica Scandinavica* **135**, 560-567.

[434] Viallet F, Pitel S, Lancrenon S, Blin O (2013) Evaluation of the safety and tolerability of rasagiline in the treatment of the early stages of Parkinson's disease. *Current Medical Research and Opinion* **29**, 23-31.

[435] Vikdahl M, Carlsson M, Linder J, Forsgren L, Haglin L (2014) Weight gain and increased central obesity in the early phase of Parkinson's disease. *Clinical Nutrition* **33**, 1132-1139.

[436] Virameteekul S, Revesz T, Jaunmuktane Z, Warner TT, De Pablo-Fernández E (2023) Clinical Diagnostic Accuracy of Parkinson's Disease: Where Do We Stand? *Mov Disord*.

[437] Wang J, Luo S, Li L (2017) DYNAMIC PREDICTION FOR MULTIPLE REPEATED MEASURES AND EVENT TIME DATA: AN APPLICATION TO PARKINSON'S DISEASE. *Annals of Applied Statistics* **11**, 1787-1809.

[438] Wang X, Jiao B, Jia XL, Wang YQ, Liu H, Zhu XY, Hao XL, Zhu Y, Xu B, Zhang SZ, Xu Q, Wang JL, Guo JF, Yan XX, Tang BS, Zhao RC, Shen L (2022) The macular inner plexiform layer thickness as an early diagnostic indicator for Parkinson's disease. *Npj Parkinsons Disease* **8**, 8.

[439] Zhang Y, Feng S, Nie K, Zhao X, Gan R, Wang L, Zhao J, Tang H, Gao L, Zhu R, Wang L, Zhang Y (2016) Catechol-O-methyltransferase Val158Met polymorphism influences prefrontal executive function in early Parkinson's disease. *J Neurol Sci* **369**, 347-353.

[440] Ahn J, Kim H, Heo J-H (2016) Behavioral and psychological symptoms in Korean patients with early Parkinson's disease. *Parkinsonism & Related Disorders* **22**, e55-e56.

[441] Akhtar RS, Mano T (2019) High serum neurofilament light chain predicts a worse fate in early parkinsonism. *Neurology* **92**, 595-596.

[442] Alamri YAS (2017) Right- versus Left-onset Parkinson's Disease: Other Psychometric Parameters...Adwani S, Yadav R, Kumar K, Chandra SR, Pal PK. Neuropsychological profile in early Parkinson’s disease: Comparison between patients with right side onset versus left side onset of motor symptoms. Ann Indian Acad Neurol 2016;19:74-8. *Annals of Indian Academy of Neurology* **20**, 162-163.

[443] Antonini A, Biundo R (2014) PARKINSON DISEASE Can dopamine transporter imaging define early PD? *Nature Reviews Neurology* **10**, 432-433.

[444] Ascherio A, Schwarzschild MA (2016) The epidemiology of Parkinson's disease: risk factors and prevention. *Lancet Neurology* **15**, 1255-1270.

[445] Bette S, Shpiner DS, Singer C, Moore H (2018) Safinamide in the management of patients with Parkinson's disease not stabilized on levodopa: a review of the current clinical evidence. *Therapeutics and Clinical Risk Management* **14**, 1737-1745.

[446] Bhattacharjee S, Chalissery AJ, Barry T, O'Connell M, Lynch T, O'Sullivan D (2017) Referral practice, reporting standards, and the impact of dopamine transporter scans done in a tertiary hospital. *Neurology India* **65**, 1264-1270.

[447] Blesa J, Trigo-Damas I, Dileone M, del Rey NLG, Hernandez LF, Obeso JA (2017) Compensatory mechanisms in Parkinson's disease: Circuits adaptations and role in disease modification. *Experimental Neurology* **298**, 148-161.
[448] Brabenec L, Mekyska J, Galaz Z, Rektorova I (2017) Speech disorders in Parkinson's disease: early diagnostics and effects of medication and brain stimulation. *Journal of Neural Transmission* **124**, 303-334.
[449] Bratsos SP, Karponis D, Saleh SN (2018) Efficacy and Safety of Deep Brain Stimulation in the Treatment of Parkinson's Disease: A Systematic Review and Meta-analysis of Randomized Controlled Trials. *Cureus* **10**, 20.
[450] Cajigal S (2021) Highlights of the AAN Updated Guideline on Treating Motor Symptoms in Early Parkinson's Disease. *Neurology Today* **21**, 13-16.
[451] Chang YJ, Li N, Jin W, Gao JS, Li Z, Wang TJ (2021) EFFICACY, SAFETY AND TOLERABILITY OF ROTIGOTINE TRANSDERMAL PATCHES FOR TREATING EARLY PARKINSON'S DISEASE: A META-ANALYSIS OF RANDOMISED CONTROLLED TRIALS. *Acta Medica Mediterranea* **37**, 2567-2572.
[452] Chen HL, Burton EA, Ross GW, Huang XM, Savica R, Abbott RD, Ascherio A, Caviness JN, Gao X, Gray KA, Hong JS, Kamel F, Jennings D, Kirshner A, Lawler C, Liu R, Miller GW, Nussbaum R, Peddada SD, Rick AC, Ritz B, Siderowf AD, Tanner CM, Troster AI, Zhang J (2013) Research on the Premotor Symptoms of Parkinson's Disease: Clinical and Etiological Implications. *Environmental Health Perspectives* **121**, 1245-1252.
[453] Cleary RT, Bucholz R (2021) Neuromodulation Approaches in Parkinson's Disease Using Deep Brain Stimulation and Transcranial Magnetic Stimulation. *Journal of Geriatric Psychiatry and Neurology* **34**, 301-309.
[454] Cox BC, Cincotta M, Espay AJ (2012) Mirror Movements in Movement Disorders: A Review. *Tremor and Other Hyperkinetic Movements* **2**, 8.
[455] de Bie RMA, Clarke CE, Espay AJ, Fox SH, Lang AE (2020) Initiation of pharmacological therapy in Parkinson's disease: when, why, and how. *Lancet Neurology* **19**, 452-461.
[456] Deuschl G, Antonini A, Costa J, Smilowska K, Berg D, Corvol JC, Fabbrini G, Ferreira J, Foltynie T, Mir P, Schrag A, Seppi K, Taba P, Ruzicka E, Selikhova M, Henschke N, Villanueva G, Moro E (2022) European Academy of Neurology/Movement Disorder Society-European Section guideline on the treatment of Parkinson's disease: I. Invasive therapies. *European Journal of Neurology* **29**, 2580-2595.
[457] Dezsi L, Vecsei L (2014) Safinamide for the treatment of Parkinson's disease. *Expert Opinion on Investigational Drugs* **23**, 729-742.
[458] Dezsi L, Vecsei L (2017) Monoamine Oxidase B Inhibitors in Parkinson's Disease. *Cns & Neurological Disorders-Drug Targets* **16**, 425-439.
[459] Dietrichs E, Odin P (2017) Algorithms for the treatment of motor problems in Parkinson's disease. *Acta Neurologica Scandinavica* **136**, 378-385.
[460] Dijkstra F, de Volder I, Viaene M, Cras P, Crosiers D (2022) Polysomnographic Predictors of Sleep, Motor, and Cognitive Dysfunction Progression in Parkinson's Disease. *Current Neurology and Neuroscience Reports* **22**, 657-674.
[461] Dooley M, Markham A (1998) Pramipexole- A review of its use in the management of early and advanced Parkinson's disease. *Drugs & Aging* **12**, 495-514.

[462] Espay AJ, Lang AE (2018) Parkinson Diseases in the 2020s and Beyond: Replacing Clinico-Pathologic Convergence With Systems Biology Divergence. *Journal of Parkinsons Disease* **8**, S59-S64.
[463] Fabbri M, Rosa MM, Abreu D, Ferreira JJ (2015) Clinical pharmacology review of safinamide for the treatment of Parkinson's disease. *Neurodegenerative Disease Management* **5**, 481-496.
[464] Fang JY, Perez A, Christine CW, Leehey M, Aminoff MJ, Boyd JT, Morgan JC, Dhall R, Nicholas AP, Bodis-Wollner I, Zweig RM, Goudreau JL, Investigators NN-P (2015) Parkinson's disease severity and use of dopaminergic medications. *Parkinsonism & Related Disorders* **21**, 297-299.
[465] Fox SH, Katzenschlager R, Lim SY, Barton B, de Bie RMA, Seppi K, Coelho M, Sampaio C, Movement Disorder Soc E (2018) International Parkinson and Movement Disorder Society Evidence-Based Medicine Review: Update on Treatments for the Motor Symptoms of Parkinson's Disease. *Movement Disorders* **33**, 1248-1266.
[466] Frampton JE (2014) Pramipexole Extended-Release: A Review of Its Use in Patients with Parkinson's Disease. *Drugs* **74**, 2175-2190.
[467] Frampton JE (2019) Rotigotine Transdermal Patch: A Review in Parkinson's Disease. *Cns Drugs* **33**, 707-718.
[468] Getz SJ, Levin B (2017) Cognitive and Neuropsychiatric Features of Early Parkinson's Disease. *Archives of Clinical Neuropsychology* **32**, 769-785.
[469] Gjerstad MD, Alves G, Maple-Grodem J (2018) Excessive Daytime Sleepiness and REM Sleep Behavior Disorders in Parkinson's Disease: A Narrative Review on Early Intervention With Implications to Neuroprotection. *Frontiers in Neurology* **9**, 6.
[470] Gouda NA, Elkamhawy A, Cho J (2022) Emerging Therapeutic Strategies for Parkinson's Disease and Future Prospects: A 2021 Update. *Biomedicines* **10**, 40.
[471] Grazynska A, Adamczewska K, Antoniuk S, Bien M, Tos M, Kufel J, Urbas W, Siuda J (2021) The Influence of Serum Uric Acid Level on Non-Motor Symptoms Occurrence and Severity in Patients with Idiopathic Parkinson's Disease and Atypical Parkinsonisms-A Systematic Review. *Medicina-Lithuania* **57**, 13.
[472] Greig SL, McKeage K (2016) Carbidopa/Levodopa ER Capsules (Rytary(®), Numient™): A Review in Parkinson's Disease. *CNS Drugs* **30**, 79-90.
[473] Gross J (2013) Exercise Beneits Patients with Parkinson's Disease. *Internal Medicine Alert* **35**, 91-92.
[474] Hauser RA, Eliaz R, Eyal E, Abler V, Schilling T (2016) Symptomatic efficacy of rasagiline in early PD: A meta-analysis. *Parkinsonism & Related Disorders* **22**, e91-e91.
[475] Hauser RA, Giladi N, Poewe W, Brotchie J, Friedman H, Oren S, Litman P (2022) P2B001 (Extended Release Pramipexole and Rasagiline): A New Treatment Option in Development for Parkinson's Disease. *Adv Ther* **39**, 1881-1894.
[476] Heng NCL, Malek N, Lawton MA, Nodehi A, Pitz V, Grosset KA, Ben-Shlomo Y, Grosset DG Striatal Dopamine Loss in Early Parkinson's Disease: Systematic Review and Novel Analysis of Dopamine Transporter Imaging. *Movement Disorders Clinical Practice*, 8.
[477] Kaasinen V, Vahlberg T, Stoessl AJ, Strafella AP, Antonini A (2021) Dopamine Receptors in Parkinson's Disease: A Meta-Analysis of Imaging Studies. *Movement Disorders* **36**, 1781-1791.

[478] Kalia SK, Lozano AM (2013) PARKINSON DISEASE Neurostimulation in PD-benefit of early surgery revealed. *Nature Reviews Neurology* **9**, 244-245.
[479] Kano O, Tsuda H, Hayashi A, Arai M (2022) Rasagiline as Adjunct to Levodopa for Treatment of Parkinson's Disease: A Systematic Review and Meta-Analysis. *Parkinsons Dis* **2022**, 4216452.
[480] Kestenbaum M, Fahn S (2015) Safety of IPX066, an extended release carbidopa-levodopa formulation, for the treatment of Parkinson's disease. *Expert Opinion on Drug Safety* **14**, 761-767.
[481] Kimber TE (2021) Approach to the patient with early Parkinson disease: diagnosis and management. *Internal Medicine Journal* **51**, 20-26.
[482] Kobylecki C (2020) Update on the diagnosis and management of Parkinson's disease. *Clinical Medicine* **20**, 393-398.
[483] Kulkarni AS, Burns MR, Brundin P, Wesson DW (2022) Linking α-synuclein-induced synaptopathy and neural network dysfunction in early Parkinson's disease. *Brain Commun* **4**, fcac165.
[484] Lauterbach EC (2016) Six psychotropics for pre-symptomatic & early Alzheimer's (MCI), Parkinson's, and Huntington's disease modification. *Neural Regeneration Research* **11**, 1712-1726.
[485] Lewis A, Galetta S (2022) Editors' Note: Long-term Effect of Regular Physical Activity and Exercise Habits in Patients With Early Parkinson Disease. *Neurology* **99**, 401-404.
[486] Liepelt-Scarfone I, Ophey A, Kalbe E (2022) Cognition in prodromal Parkinson's disease. *Prog Brain Res* **269**, 93-111.
[487] Loh HW, Hong WR, Ooi CP, Chakraborty S, Barua PD, Deo RC, Soar J, Palmer EE, Acharya UR (2021) Application of Deep Learning Models for Automated Identification of Parkinson's Disease: A Review (2011-2021). *Sensors* **21**, 25.
[488] Lyons KE, Pahwa R (2013) Outcomes of Rotigotine Clinical Trials: Effects on Motor and Nonmotor Symptoms of Parkinson's Disease. *Neurologic Clinics* **31**, S51-+.
[489] Malkki H (2013) Parkinson disease: Nonmotor symptoms predict quality of life in patients with early Parkinson disease. *Nat Rev Neurol* **9**, 544.
[490] Martinez-Martin P (2014) Nonmotor symptoms and health-related quality of life in early Parkinson's disease. *Mov Disord* **29**, 166-168.
[491] Matheson AJ, Spencer CM (2000) Ropinirole- A review of its use in the management of Parkinson's disease. *Drugs* **60**, 115-137.
[492] McCormack PL (2014) Rasagiline: a review of its use in the treatment of idiopathic Parkinson's disease. *CNS Drugs* **28**, 1083-1097.
[493] McDaniels B, Pontone GM, Keener AM, Subramanian I A Prescription for Wellness in Early PD: Just What the Doctor Ordered. *Journal of Geriatric Psychiatry and Neurology*, 9.
[494] Metta V, Leta V, Mrudula KR, Prashanth LK, Goyal V, Borgohain R, Chung-Faye G, Chaudhuri KR (2022) Gastrointestinal dysfunction in Parkinson's disease: molecular pathology and implications of gut microbiome, probiotics, and fecal microbiota transplantation. *Journal of Neurology* **269**, 1154-1163.
[495] Mukherjee A, Biswas A, Das SK (2016) Gut dysfunction in Parkinson's disease. *World Journal of Gastroenterology* **22**, 5742-5752.

[496] Nakum S, Cavanna AE (2016) The prevalence and clinical characteristics of hypersexuality in patients with Parkinson's disease following dopaminergic therapy: A systematic literature review. *Parkinsonism & Related Disorders* **25**, 10-16.

[497] Noci A, Wolbers M, Abt M, Baayen C, Burger HU, Jin M, Robieson WNZ A Comparison of Estimand and Estimation Strategies for Clinical Trials in Early Parkinson's Disease. *Statistics in Biopharmaceutical Research*, 11.

[498] Obeso JA, Burgera JA, Castro A, Chacon J, Roldan SG, Grandas F, Horga J, Kulisevski J, Lozano JJL, Macias R, Marin C, Pavon N, Diaz MR, Oroz MCR, Valldeoriola F (2002) Consensus on the use of entacapone in Parkinson's disease. *Neurologia* **17**, 77-79.

[499] Ortner NJ (2021) Voltage-Gated Ca2+ Channels in Dopaminergic Substantia Nigra Neurons: Therapeutic Targets for Neuroprotection in Parkinson's Disease? *Frontiers in Synaptic Neuroscience* **13**, 11.

[500] Pagonabarraga J, Rodriguez-Oroz MC (2013) Rasagiline in monotherapy in patients with early stages of Parkinson's disease and in combined and adjunct therapy to levodopa with moderate and advanced stages. *Revista De Neurologia* **56**, 25-34.

[501] Parambi DT (2020) Treatment of Parkinson's Disease by MAO-B Inhibitors, New Therapies and Future Challenges- A Mini-Review. *Combinatorial Chemistry & High Throughput Screening* **23**, 847-861.

[502] Perez-Lloret S, Rascol O (2016) Piribedil for the Treatment of Motor and Non-motor Symptoms of Parkinson Disease. *CNS Drugs* **30**, 703-717.

[503] Postuma RB (2019) Prodromal Parkinson disease: do we miss the signs? *Nature Reviews Neurology* **15**, 437-438.

[504] Postuma RB, Berg D (2016) Advances in markers of prodromal Parkinson disease. *Nature Reviews Neurology* **12**, 622-634.

[505] Pringsheim T, Day GS, Smith DB, Rae-Grant A, Licking N, Armstrong MJ, de Bie RMA, Roze E, Miyasaki JM, Hauser RA, Espay AJ, Martello JP, Gurwell JA, Billinghurst L, Sullivan K, Fitts MS, Cothros N, Hall DA, Rafferty M, Hagerbrant L (2021) Dopaminergic Therapy for Motor Symptoms in Early Parkinson Disease Practice Guideline Summary: A Report of the AAN Guideline Subcommittee. *Neurology* **97**, 942-957.

[506] Ravn AH, Thyssen JP, Egeberg A (2017) Skin disorders in Parkinson's disease: potential biomarkers and risk factors. *Clinical Cosmetic and Investigational Dermatology* **10**, 87-92.

[507] Robinson JP, Bradway CW, Bunting-Perry L, Moriarty H (2019) Increased odds of bladder and bowel symptoms in early Parkinson's disease. *Neurourol Urodyn* **38**, 418-419.

[508] Rossi C, Genovesi D, Marzullo P, Giorgetti A, Filidei E, Corsini GU, Bonuccelli U, Ceravolo R (2017) Striatal Dopamine Transporter Modulation After Rotigotine: Results From a Pilot Single-Photon Emission Computed Tomography Study in a Group of Early Stage Parkinson Disease Patients. *Clinical Neuropharmacology* **40**, 34-36.

[509] Sakakibara R, Tateno F, Kishi M, Tsuyusaki Y, Terada H, Inaoka T (2014) MIBG myocardial scintigraphy in pre-motor Parkinson's disease: A review. *Parkinsonism & Related Disorders* **20**, 267-273.

[510] Seibyl J, Russell D, Jennings D, Marek K (2012) Neuroimaging Over the Course of Parkinson's Disease: From Early Detection of the At-Risk Patient to Improving Pharmacotherapy of Later-Stage Disease. *Seminars in Nuclear Medicine* **42**, 406-414.

[511] Sharpe G, Macerollo A, Fabbri M, Tripoliti E (2020) Non-pharmacological Treatment Challenges in Early Parkinson's Disease for Axial and Cognitive Symptoms: A Mini Review. *Frontiers in Neurology* **11**, 11.
[512] Shrestha N, Abe RAM, Masroor A, Khorochkov A, Prieto J, Singh KB, Nnadozie MC, Abdal M, Mohammed L (2021) The Correlation Between Parkinson's Disease and Rapid Eye Movement Sleep Behavior Disorder: A Systematic Review. *Cureus Journal of Medical Science* **13**, 9.
[513] Sigurdsson HP, Raw R, Hunter H, Baker MR, Taylor JP, Rochester L, Yarnall AJ (2021) Noninvasive vagus nerve stimulation in Parkinson's disease: current status and future prospects. *Expert Review of Medical Devices* **18**, 971-984.
[514] Solari N, Bonito-Oliva A, Fisone G, Brambilla R (2013) Understanding cognitive deficits in Parkinson's disease: lessons from preclinical animal models. *Learning & Memory* **20**, 592-600.
[515] Solmi M, Pigato G, Kane JM, Correll CU (2018) Clinical risk factors for the development of tardive dyskinesia. *Journal of the Neurological Sciences* **389**, 21-27.
[516] Stephenson D, Hill D, Cedarbaum JM, Tome M, Vamvakas S, Romero K, Conrado DJ, Dexter DT, Seibyl J, Jennings D, Nicholas T, Matthews D, Xie ZY, Imam S, Maguire P, Russell D, Gordon MF, Stebbin GT, Somer E, Gallagher J, Roach A, Basseches P, Grosset D, Marek K, Critical Path Parkinsons C (2019) The Qualification of an Enrichment Biomarker for Clinical Trials Targeting Early Stages of Parkinson's Disease. *Journal of Parkinsons Disease* **9**, 553-563.
[517] Supriya P, Rajaram S (2021) Literature Review on History and Pharmacotherapy of Parkinson`s Disease. *Journal of Pharmaceutical Research International* **33**, 839-849.
[518] Taniguchi S, Takeda A (2017) Olfactory dysfunction. *Nihon Rinsho* **75**, 119-123.
[519] Tseng MT, Lin CH (2017) Pain in early-stage Parkinson's disease: Implications from clinical features to pathophysiology mechanisms. *Journal of the Formosan Medical Association* **116**, 571-581.
[520] Tsukita K, Sakamaki-Tsukita H, Takahashi R (2022) Author Response: Long-term Effect of Regular Physical Activity and Exercise Habits in Patients With Early Parkinson Disease. *Neurology* **99**, 132-132.
[521] van Hilten JJ, Ramaker CC, Stowe RL, Ives NJ (2007) Bromocriptine versus levodopa in early Parkinson's disease. *Cochrane Database of Systematic Reviews*, 32.
[522] Wiseman LR, Fitton A (1999) Cabergoline- A review of its efficacy in the treatment of Parkinson's disease. *Cns Drugs* **12**, 485-497.
[523] Wiseman LR, McTavish D (1995) SELEGILINE- A REVIEW OF ITS CLINICAL EFFICACY IN PARKINSONS-DISEASE AND ITS CLINICAL POTENTIAL IN ALZHEIMERS-DISEASE. *Cns Drugs* **4**, 230-246.
[524] Xie CL, Zhang YY, Wang XD, Chen J, Chen YH, Pa JL, Lin SY, Lin HZ, Wang WW (2015) Levodopa alone compared with levodopa-sparing therapy as initial treatment for Parkinson's disease: a meta-analysis. *Neurological Sciences* **36**, 1319-1329.
[525] Yee KM, Ford PJ (2012) Regulatory misconception muddies the ethical waters: challenges to a qualitative study. *J Clin Ethics* **23**, 217-220; discussion 221-213.
[526] Zesiewicz TA, Bezchlibnyk Y, Dohse N, Ghanekar SD (2020) Management of Early Parkinson Disease. *Clinics in Geriatric Medicine* **36**, 35-+.

[527] Zhang H, Song C, Rathore AS, Huang MC, Zhang Y, Xu W (2021) mHealth Technologies Towards Parkinson's Disease Detection and Monitoring in Daily Life: A Comprehensive Review. *IEEE Rev Biomed Eng* **14**, 71-81.

[528] Zhang J (2022) Mining imaging and clinical data with machine learning approaches for the diagnosis and early detection of Parkinson's disease. *Npj Parkinsons Disease* **8**, 15.

[529] Zhang Y, Burock MA (2020) Diffusion Tensor Imaging in Parkinson's Disease and Parkinsonian Syndrome: A Systematic Review. *Frontiers in Neurology* **11**, 25.

[530] Zhao YT, Liu L, Zhao Y, Xie ZY (2022) The effect and safety of levodopa alone versus levodopa sparing therapy for early Parkinson's disease: a systematic review and meta-analysis. *Journal of Neurology* **269**, 1834-1850.

[531] Zhou CQ, Zhang JW, Wang M, Peng GG (2014) Meta-analysis of the efficacy and safety of long-acting non-ergot dopamine agonists in Parkinson's disease. *Journal of Clinical Neuroscience* **21**, 1094-1101.

[532] 高中宝, 王洁, 王炜, 陈彤, 孙虹, 王振福 (2015) 帕金森病诊断与治疗断进展. *Chinese Journal of Contemporary Neurology & Neurosurgery* **15**, 777-781.

[533] Blandini F (2005) Neuroprotection by rasagiline: a new therapeutic approach to Parkinson's disease? *CNS Drug Rev* **11**, 183-194.

[534] Csanda E, Tárczy M, Takáts A, Mogyorós I, Köves A, Katona G (1983) L-deprenyl in the treatment of Parkinson's disease. *J Neural Transm Suppl* **19**, 283-290.

[535] Doan JB, Melvin KG, Whishaw IQ, Suchowersky O (2008) Bilateral impairments of skilled reach-to-eat in early Parkinson's disease patients presenting with unilateral or asymmetrical symptoms. *Behavioural Brain Research* **194**, 207-213.

[536] Giladi N, McDermott MP, Fahn S, Przedborski S, Jankovic J, Stern M, Tanner C, Parkinson Study G (2001) Freezing of gait in PD- Prospective assessment in the DATATOP cohort. *Neurology* **56**, 1712-1721.

[537] Hatori K, Kondo T, Mizuno Y (1996) Levodopa absorption profile in Parkinson's disease: Evidence to indicate qualitative difference from the control. *Parkinsonism Relat Disord* **2**, 137-144.

[538] Lieberman A, Fazzini E (1991) Experience with selegiline and levodopa in advanced Parkinson's disease. *Acta Neurol Scand Suppl* **136**, 66-69.

[539] Lieberman AN, Goldstein M, Gopinathan G, Neophytides A (1987) D-1 and D-2 agonists in Parkinson's disease. *Can J Neurol Sci* **14**, 466-473.

[540] Przuntek H, Welzel D, Blümner E, Danielczyk W, Letzel H, Kaiser HJ, Kraus PH, Riederer P, Schwarzmann D, Wolf H, et al. (1992) Bromocriptine lessens the incidence of mortality in L-dopa-treated parkinsonian patients: prado-study discontinued. *Eur J Clin Pharmacol* **43**, 357-363.

[541] Ramaker C, van Hilten JJ (2000) Bromocriptine versus levodopa in early Parkinson's disease. *Cochrane Database Syst Rev*, Cd002258.

[542] Alhussein M (2017) Monitoring Parkinson's Disease in Smart Cities. *Ieee Access* **5**, 19835-19841.

[543] Almogren A (2019) An automated and intelligent Parkinson disease monitoring system using wearable computing and cloud technology. *Cluster Computing-the Journal of Networks Software Tools and Applications* **22**, 2309-2316.

[544] Bernardo LS, Quezada A, Munoz R, Maia FM, Pereira CR, Wu WQ, de Albuquerque VHC (2019) Handwritten pattern recognition for early Parkinson's disease diagnosis. *Pattern Recognition Letters* **125**, 78-84.

[545] Biglan KM, Oakes D, Lang AE, Hauser RA, Hodgeman K, Greco B, Lowell J, Rockhill R, Shoulson I, Venuto C, Young D, Simuni T, Parkinson Study Grp S-P, III (2017) A novel design of a Phase III trial of isradipine in early Parkinson disease (STEADY-PD III). *Annals of Clinical and Translational Neurology* **4**, 360-368.

[546] Dusek P, Bezdicek O, Brozova H, Dall'Antonia I, Dostalova S, Havrankova P, Klempir J, Mana J, Maskova J, Nepozitek J, Roth J, Perinova P, Ruzicka F, Serranova T, Trnka J, Ulmanova O, Zogala D, Jech R, Sonka K, Ruzicka E (2020) Clinical characteristics of newly diagnosed Parkinson's disease patients included in the longitudinal BIO-PD study. *Ceska a Slovenska Neurologie a Neurochirurgie* **83**, 633-639.

[547] Ferreira JJ, Poewe W, Rascol O, Stocchi F, Antonini A, Moreira J, Pereira A, Rocha JF, Soares-da-Silva P (2022) Opicapone as an Add-on to Levodopa in Patients with Parkinson's Disease Without Motor Fluctuations: Rationale and Design of the Phase III, Double-Blind, Randomised, Placebo-Controlled EPSILON Trial. *Neurology and Therapy* **11**, 1409-1425.

[548] Jiang L, Wang XX, Li PT, Feng ZH, Shi X, Shao H (2020) Efficacy of C-11-2 beta-carbomethoxy-3 beta-(4-fluorophenyl) tropane positron emission tomography combined with F-18-fluorodeoxyglucose positron emission tomography in the diagnosis of early Parkinson disease A protocol for systematic review and meta analysis. *Medicine* **99**, 5.

[549] Siepmann T, Pinter A, Buchmann SJ, Stibal L, Arndt M, Kubasch AS, Kubasch ML, Penzlin AI, Frenz E, Zago W, Horvath T, Szatmari S, Bereczki D, Takats A, Ziemssen T, Lipp A, Freeman R, Reichmann H, Barlinn K, Illigens BMW (2017) Cutaneous Autonomic Pilomoter Testing to Unveil the Role of Neuropathy Progression in Early Parkinson's Disease (CAPTURE PD): Protocol for a Multicenter Study. *Frontiers in Neurology* **8**, 9.

[550] Verschuur CV, Suwijn SR, Post B, Dijkgraaf M, Bloem BR, van Hilten JJ, van Laar T, Tissingh G, Deuschl G, Lang AE, de Haan RJ, de Bie RM (2015) Protocol of a randomised delayed-start double-blind placebo-controlled multi-centre trial for Levodopa in EArly Parkinson's disease: the LEAP-study. *BMC Neurol* **15**, 236.

[551] Fereshtehnejad SM, Yao C, Pelletier A, Montplaisir JY, Gagnon JF, Postuma RB (2019) Evolution of prodromal Parkinson's disease and dementia with Lewy bodies: a prospective study. *Brain* **142**, 2051-2067.

[552] Flores-Torres MH, Hughes KC, Molsberry S, Gao X, Kang JH, Schwarzschild MA, Ascherio A (2021) Cognitive function in men with non-motor features of Parkinson's disease. *BMJ Neurol Open* **3**, e000112.

[553] Friederich A, Flinspach A, Suenkel U, Eschweiler GW, Greulich K, team Ts, Maetzler W, Berg D, Heinzel S (2019) Prodromal features of Parkinson's disease: Self-reported symptoms versus clinically assessed signs. *Mov Disord* **34**, 144-146.

[554] Giorelli M, Bagnoli J, Consiglio L, Lopane M, Zimatore GB, Zizza D, Difazio P (2014) Do non-motor symptoms in Parkinson's disease differ from essential tremor before initial diagnosis? A clinical and scintigraphic study. *Parkinsonism Relat Disord* **20**, 17-21.

[555] Kresojevic N, Jankovic M, Petrovic I, Kumar KR, Dragasevic N, Dobricic V, Novakovic I, Svetel M, Klein C, Pekmezovic T, Kostic VS (2015) Presenting symptoms of GBA-related Parkinson's disease. *Parkinsonism Relat Disord* **21**, 804-807.

[556] Liu H, Ou R, Wei Q, Hou Y, Cao B, Zhao B, Shang H (2019) Rapid eye movement behavior disorder in drug-naive patients with Parkinson's disease. *J Clin Neurosci* **59**, 254-258.
[557] Liu SY, Zheng Z, Gu ZQ, Wang CD, Tang BS, Xu YM, Ma JH, Zhou YT, Feng T, Chen SD, Chan P, Chinese Parkinson Study G (2018) Prevalence of pre-diagnostic symptoms did not differ between LRRK2-related, GBA-related and idiopathic patients with Parkinson's disease. *Parkinsonism Relat Disord* **57**, 72-76.
[558] Maraki MI, Stefanis L, Yannakoulia M, Kosmidis MH, Xiromerisiou G, Dardiotis E, Hadjigeorgiou GM, Sakka P, Scarmeas N, Stamelou M (2019) Motor function and the probability of prodromal Parkinson's disease in older adults. *Mov Disord* **34**, 1345-1353.
[559] Marano M, Gupta D, Motolese F, Rossi M, Luccarelli V, Altamura C, Di Lazzaro V (2020) Excessive daytime sleepiness is associated to the development of swallowing impairment in a cohort of early stage drug naive Parkinson's disease patients. *J Neurol Sci* **410**, 116626.
[560] Orso B, Fama F, Giorgetti L, Mattioli P, Donniaquio A, Girtler N, Brugnolo A, Massa F, Peira E, Pardini M, Morbelli S, Nobili F, Arnaldi D (2022) Polysomnographic correlates of sleep disturbances in de novo, drug naive Parkinson's Disease. *Neurol Sci* **43**, 2531-2536.
[561] Pagonabarraga J, Martinez-Horta S, Fernandez de Bobadilla R, Perez J, Ribosa-Nogue R, Marin J, Pascual-Sedano B, Garcia C, Gironell A, Kulisevsky J (2016) Minor hallucinations occur in drug-naive Parkinson's disease patients, even from the premotor phase. *Mov Disord* **31**, 45-52.
[562] Plouvier AO, Hameleers RJ, van den Heuvel EA, Bor HH, Olde Hartman TC, Bloem BR, van Weel C, Lagro-Janssen AL (2014) Prodromal symptoms and early detection of Parkinson's disease in general practice: a nested case-control study. *Fam Pract* **31**, 373-378.
[563] Pont-Sunyer C, Tolosa E, Caspell-Garcia C, Coffey C, Alcalay RN, Chan P, Duda JE, Facheris M, Fernandez-Santiago R, Marek K, Lomena F, Marras C, Mondragon E, Saunders-Pullman R, Waro B, Consortium LC (2017) The prodromal phase of leucine-rich repeat kinase 2-associated Parkinson disease: Clinical and imaging Studies. *Mov Disord* **32**, 726-738.
[564] Rodriguez-Violante M, de Sarachaga AJ, Cervantes-Arriaga A, Millan-Cepeda R, Leal-Ortega R, Estrada-Bellmann I, Zuniga-Ramirez C (2016) Self-Perceived Pre-Motor Symptoms Load in Patients with Parkinson's Disease: A Retrospective Study. *J Parkinsons Dis* **6**, 183-190.
[565] Schrag A, Horsfall L, Walters K, Noyce A, Petersen I (2015) Prediagnostic presentations of Parkinson's disease in primary care: a case-control study. *Lancet Neurol* **14**, 57-64.
[566] Schrag A, Zhelev SS, Hotham S, Merritt RD, Khan K, Graham L (2019) Heterogeneity in progression of prodromal features in Parkinson's disease. *Parkinsonism Relat Disord* **64**, 275-279.
[567] Walter U, Kleinschmidt S, Rimmele F, Wunderlich C, Gemende I, Benecke R, Busse K (2013) Potential impact of self-perceived prodromal symptoms on the early diagnosis of Parkinson's disease. *J Neurol* **260**, 3077-3085.
[568] Watts CR, Zhang Y (2022) Progression of Self-Perceived Speech and Swallowing Impairment in Early Stage Parkinson's Disease: Longitudinal Analysis of the Unified Parkinson's Disease Rating Scale. *J Speech Lang Hear Res* **65**, 146-158.

[569] Zimmermann M, Gaenslen A, Prahl K, Srulijes K, Hauser AK, Schulte C, Csoti I, Berg D, Brockmann K (2019) Patient's perception: shorter and more severe prodromal phase in GBA-associated PD. *Eur J Neurol* **26**, 694-698.
[570] Alsunaid S, Holden VK, Kohli A, Diaz J, O'Meara LB (2021) Wound care management: tracheostomy and gastrostomy. *Journal of Thoracic Disease* **13**, 5297-5313.
[571] Bienvenut WV, Giglione C, Meinnel T (2015) Proteome-wide analysis of the amino terminal status of Escherichia coli proteins at the steady-state and upon deformylation inhibition. *Proteomics* **15**, 2503-2518.
[572] Boakye LAT, Fourman MS, Spina NT, Laudermilch D, Lee JY (2018) "Post-Decompressive Neuropathy": New-Onset Post-Laminectomy Lower Extremity Neuropathic Pain Different from the Preoperative Complaint. *Asian Spine Journal* **12**, 1043-1052.
[573] Caputo D, Digiacomo L, Cascone C, Pozzi D, Palchetti S, Di Santo R, Quagliarini E, Coppola R, Mahmoudi M, Caracciolo G (2021) Synergistic Analysis of Protein Corona and Haemoglobin Levels Detects Pancreatic Cancer. *Cancers* **13**, 11.
[574] Chandran S, Nikfarjam M (2013) The utility of upfront double wire guided biliary cannulation following early unintentional pancreatic cannulation in patients undergoing ERCP. *Indian Journal of Gastroenterology* **32**, 324-329.
[575] Chen YJ, Chen YC, Dong HL, Li LX, Ni W, Li HF, Wu ZY (2018) Novel PLA2G6 mutations and clinical heterogeneity in Chinese cases with phospholipase A2-associated neurodegeneration. *Parkinsonism & Related Disorders* **49**, 88-94.
[576] Chen ZJ, Han CY, Zhou X, Wang XK, Liao XW, He YF, Mo ST, Li X, Zhu GZ, Ye XP, Peng T (2021) Prognostic value and potential molecular mechanism of the like-Sm gene family in early-stage pancreatic ductal adenocarcinoma. *Translational Cancer Research* **10**, 1744-+.
[577] Clyman RI, Hills NK, Cambonie G, Debillon T, Ligi I, Gascoin G, Patkai J, Beuchee A, Favrais G, Durrmeyer X, Flamant C, Roze JC (2022) Patent ductus arteriosus, tracheal ventilation, and the risk of bronchopulmonary dysplasia. *Pediatric Research* **91**, 652-658.
[578] Grandin EW, Gulati G, Nunez JI, Kennedy K, Rame JE, Atluri P, Pagani FD, Kirklin JK, Kormos RL, Teuteberg J, Kiernan MS (2022) Outcomes With Phosphodiesterase-5 Inhibitor Use After Left Ventricular Assist Device: An STS-INTERMACS Analysis. *Circulation-Heart Failure* **15**, 307-317.
[579] Guttman Krader C (2021) Study highlights negative mental health impact of Peyronie disease. *Urology Times* **49**, 27-32.
[580] Hagiwara A, Schlossman J, Shabani S, Raymond C, Tatekawa H, Abrey LE, Garcia J, Chinot O, Saran F, Nishikawa R, Henriksson R, Mason WP, Wick W, Cloughesy TF, Ellingson BM (2022) Incidence, molecular characteristics, and imaging features of "clinically-defined pseudoprogression" in newly diagnosed glioblastoma treated with chemoradiation. *Journal of Neuro-Oncology* **159**, 509-518.
[581] Herreros-Villanueva M, Gironella M, Castells A, Bujanda L (2013) Molecular markers in pancreatic cancer diagnosis. *Clinica Chimica Acta* **418**, 22-29.
[582] Hidalgo M, Cascinu S, Kleeff J, Labianca R, Lohr JM, Neoptolemos J, Real FX, Van Laethem JL, Heinemann V (2015) Addressing the challenges of pancreatic cancer: Future directions for improving outcomes. *Pancreatology* **15**, 8-18.

[583] Ingel B, Reyes C, Massonnet M, Boudreau B, Sun YL, Sun Q, McElrone AJ, Cantu D, Roper MC (2021) Xylella fastidiosa causes transcriptional shifts that precede tylose formation and starch depletion in xylem. *Molecular Plant Pathology* **22**, 175-188.
[584] Jaworski JJ, Morgan RD, Sivakumar S (2020) Circulating Cell-Free Tumour DNA for Early Detection of Pancreatic Cancer. *Cancers* **12**, 16.
[585] Kaese S, Zander MC, Lebiedz P (2016) Successful Use of Early Percutaneous Dilatational Tracheotomy and the No Sedation Concept in Respiratory Failure in Critically Ill Obese Subjects. *Respiratory Care* **61**, 615-620.
[586] Kurk SA, Peeters PHM, Dorresteijn B, de Jong PA, Jourdan M, Creemers GJM, Erdkamp FLG, de Jongh FE, Kint PAM, Poppema BJ, Radema SA, Simkens LHJ, Tanis BC, Tjin-A-Ton MLR, Van Der Velden A, Punt CJA, Koopman M, May AM (2020) Loss of skeletal muscle index and survival in patients with metastatic colorectal cancer: Secondary analysis of the phase 3 CAIRO3 trial. *Cancer Medicine* **9**, 1033-1043.
[587] Liao SY, Towie EA, Balaz D, Riddet C, Cheng BJ, Asenov A, Ieee (2013) in *18th International Conference on Simulation of Semiconductor Processes and Devices (SISPAD)* Ieee, Univ Glasgow, Glasgow, SCOTLAND, pp. 220-223.
[588] Ng AKH, Tan SN, Tay ME, Van Der Straaten JC, Chionh CY, Grp C (2022) Comparison of planned-start, early-start and deferred-start strategies for peritoneal dialysis initiation in end-stage kidney disease. *Annals Academy of Medicine Singapore* **51**, 213-220.
[589] Noor-ul-huda M, Tehsin S, Ahmed S, Niazi FAK, Murtaza Z (2019) Retinal images benchmark for the detection of diabetic retinopathy and clinically significant macular edema (CSME). *Biomedical Engineering-Biomedizinische Technik* **64**, 297-307.
[590] Obolonskyi A, Snisar V, Surkov D, Obolonska O, Kapustina O, Dereza K (2019) MANAGEMENT OF PATENT DUCTUS ARTERIOSUS IN PREMATURE INFANTS. *Medical Perspectives-Medicni Perspektivi* **24**, 33-40.
[591] Perez-Donoso AG, Lenhof JJ, Pinney K, Labavitch JM (2016) Vessel embolism and tyloses in early stages of Pierce's disease. *Australian Journal of Grape and Wine Research* **22**, 81-86.
[592] Pilarczyk K, Marggraf G, Dudasova M, Demircioglu E, Scheer V, Jakob H, Dusse F (2015) Tracheostomy After Cardiac Surgery With Median Sternotomy and Risk of Deep Sternal Wound Infections: Is It a Matter of Timing? *Journal of Cardiothoracic and Vascular Anesthesia* **29**, 1573-1581.
[593] Powrozek T, Kowalski DM, Krawczyk P, Ramlau R, Kucharczyk T, Kalinka-Warzocha E, Knetki-Wroblewska M, Winiarczyk K, Dyszkiewicz W, Krzakowski M, Milanowski J (2014) Correlation Between TS, MTHFR, and ERCC1 Gene Polymorphisms and the Efficacy of Platinum in Combination With Pemetrexed First-Line Chemotherapy in Mesothelioma Patients. *Clinical Lung Cancer* **15**, 455-465.
[594] Punjani N, Nascimento B, Salter C, Miranda E, Terrier J, Taniguchi H, Jenkins L, Mulhall JP (2021) Predictors of Depression in Men With Peyronie's Disease Seeking Evaluation. *Journal of Sexual Medicine* **18**, 783-788.
[595] Qu X, Houser SH, Tian MR, Zhang Q, Pan J, Zhang W (2022) Effects of early preventive dental visits and its associations with dental caries experience: a cross-sectional study. *Bmc Oral Health* **22**, 9.

[596] Saida K, Nakamura T, Hiroma T, Takigiku K, Yasukochi S (2013) Preoperative left ventricular internal dimension in end-diastole as earlier identification of early patent ductus arteriosus operation and postoperative intensive care in very low birth weight infants. *Early Human Development* **89**, 821-823.

[597] Senthil K, Saravanasankar P, Bagavathshalini M, Hemmanthraj M, Sruthi RS, Cinushamasilthilakraj FXR, Shaminabegum AH (2016) AN ANALYTICAL STUDY TO EVALUATE SEVERITY OF DIABETIC RETINOPATHY AND INCIDENCE OF NEUROPATHY, NEPHROPATHY IN PATIENTS WITH TYPE 2 DIABETES MELLITUS. *Journal of Evolution of Medical and Dental Sciences-Jemds* **5**, 6875-6878.

[598] Tang JZ, Xiao DP, Wang J, Li Y, Bai HZ, Pan XB (2022) Optimizing planting dates and cultivars can enhance China's potato yield under 1.5 degrees C and 2.0 degrees C global warming. *Agricultural and Forest Meteorology* **324**, 9.

[599] Vega EA, Kutlu OC, Salehi O, James D, Alarcon SV, Herrick B, Krishnan S, Kozyreva O, Conrad C (2020) Preoperative Chemotherapy for Pancreatic Cancer Improves Survival and R0 Rate Even in Early Stage I. *Journal of Gastrointestinal Surgery* **24**, 2409-2415.

[600] Ward MJ (2021) A Novel Approach to the Symptomatic Management of Chronic Megacolon. *Case Reports in Surgery* **2021**, 3.

[601] Wu HW, Guo SW, Liu XD, Li YT, Su ZX, He QY, Liu XQ, Zhang ZW, Yu LY, Shi XH, Gao SZ, Wang H, Pan YQ, Ma CC, Liu R, Dai MH, Jin G, Liang ZY (2022) Noninvasive detection of pancreatic ductal adenocarcinoma using the methylation signature of circulating tumour DNA. *Bmc Medicine* **20**, 17.

[602] Yang F, Jin C, Hao SJ, Fu DL (2019) Drain Contamination after Distal Pancreatectomy: Incidence, Risk Factors, and Association with Postoperative Pancreatic Fistula. *Journal of Gastrointestinal Surgery* **23**, 2449-2458.

[603] Zhang JY, Zhang Z, Jin B, Zhang SY, Zhou CB, Fu JL, Wang FS (2008) Programmed death-1 up-regulation is involved in the attrition of cytomegalovirus-specific CD8(+) T cells in acute self-limited hepatitis B virus infection. *Journal of Immunology* **181**, 3741-3744.

[604] Acharya M, Banerjee S, Chatterjee A, Mukherjee A, Biswas S, Gangopadhyay G, Biswas A (2021) Predicting Long-Term Outcome of Patients of Early Parkinsonism with Acute Levodopa Challenge Test. *Neurology India* **69**, 430-434.

[605] Adams WR (2017) High-accuracy detection of early Parkinson's Disease using multiple characteristics of finger movement while typing. *Plos One* **12**, 20.

[606] Arroyo-Gallego T, Ledesma-Carbayo MJ, Butterworth I, Matarazzo M, Montero-Escribano P, Puertas-Martin V, Gray ML, Giancardo L, Sanchez-Ferro A (2018) Detecting Motor Impairment in Early Parkinson's Disease via Natural Typing Interaction With Keyboards: Validation of the neuroQWERTY Approach in an Uncontrolled At-Home Setting. *Journal of Medical Internet Research* **20**, 14.

[607] Bartl M, Dakna M, Schade S, Otte B, Wicke T, Lang E, Starke M, Ebentheuer J, Weber S, Toischer K, Schnelle M, Sixel-Döring F, Trenkwalder C, Mollenhauer B (2023) Blood Markers of Inflammation, Neurodegeneration, and Cardiovascular Risk in Early Parkinson's Disease. *Mov Disord* **38**, 68-81.

[608] Brakedal B, Tysnes OB, Skeie GO, Larsen JP, Muller B (2014) The factor structure of the UPDRS motor scores changes during early Parkinson's disease. *Parkinsonism & Related Disorders* **20**, 617-621.

[609] Byeon H (2022) Development of the best ensemble-based machine learning classifier for distinguishing hypokinetic dysarthria caused by Parkinson's disease from presbyphonia and comparison of performance measures...International Society for Gerontechnology 13th World Conference, October 24-26, 2022, Daegu, South Korea. *Gerontechnology* **21**, 1-1.

[610] Combs HL, Wyman-Chick KA, Erickson LO, York MK (2021) Development of standardized regression-based formulas to assess meaningful cognitive change in early Parkinson's disease. *Archives of Clinical Neuropsychology* **36**, 734-745.

[611] Epprecht L, Schreglmann SR, Goetze O, Woitalla D, Baumann CR, Waldvogel D (2015) Unchanged gastric emptying and visceral perception in early Parkinson's disease after a high caloric test meal. *Journal of Neurology* **262**, 1946-1953.

[612] Faust IM, Racette BA, Nielsen SS (2020) Validation of a Parkinson Disease Predictive Model in a Population-Based Study. *Parkinsons Disease* **2020**, 7.

[613] Grammatikopoulou A, Grammalidis N, Bostantjopoulou S, Katsarou Z, Acm (2019) in *12th ACM International Conference on PErvasive Technologies Related to Assistive Environments (PETRA)* Assoc Computing Machinery, Rhodes, GREECE, pp. 517-522.

[614] Greenland JC, Cutting E, Kadyan S, Bond S, Chhabra A, Williams-Gray CH (2020) Azathioprine immunosuppression and disease modification in Parkinson's disease (AZA-PD): a randomised double-blind placebo-controlled phase II trial protocol. *Bmj Open* **10**, 10.

[615] Iakovakis D, Hadjidimitriou S, Charisis V, Bostantzopoulou S, Katsarou Z, Hadjileontiadis LJ (2018) Touchscreen typing-pattern analysis for detecting fine motor skills decline in early-stage Parkinson's disease. *Scientific Reports* **8**, 13.

[616] Jeancolas L, Mangonez G, Corvol JC, Vidailhet M, Lehericy S, Benkelfat BE, Benali H, Petrovska-Delacretaz D, Int Speech Commun A (2019) in *Interspeech Conference* Isca-Int Speech Communication Assoc, Graz, AUSTRIA, pp. 3033-3037.

[617] Kwon MS, Kim Y, Lee S, Namkung J, Yun T, Yi SG, Han S, Kang M, Kim SW, Jang JY, Park T (2014) in *IEEE International Conference on Bioinformatics and Biomedicine (IEEE BIBM)* Ieee, Univ Ulster, Belfast, NORTH IRELAND.

[618] Laganas C, Iakovakis D, Hadjidimitriou S, Charisis V, Dias SB, Bostantzopoulou S, Katsarou Z, Klingelhoefer L, Reichmann H, Trivedi D, Chaudhuri KR, Hadjileontiadis LJ (2022) Parkinson's Disease Detection Based on Running Speech Data From Phone Calls. *Ieee Transactions on Biomedical Engineering* **69**, 1573-1584.

[619] Lee S, Oh SH, Park SW, Shin C, Kim J, Rhim JH, Lee JY, Choi JY (2020) Screening Patients with Early Stage Parkinson's Disease Using a Machine Learning Technique: Measuring the Amount of Iron in the Basal Ganglia. *Applied Sciences-Basel* **10**, 13.

[620] Mun JK, Youn J, Cho JW, Oh ES, Kim JS, Park S, Jang W, Park JS, Koh SB, Lee JH, Park HK, Kim HJ, Jeon BS, Shin HW, Choi SA, Kim SJ, Choi SM, Park JY, Kim JY, Chung SJ, Lee CS, Ahn TB, Kim WC, Kim HS, Cheon SM, Kim JW, Kim HT, Lee JY, Kim JS, Kim EJ, Kim JM, Lee KS, Kim JS, Kim MJ, Baik JS, Park KJ, Kim HJ, Park MY, Kang JH, Song SK, Kim YD, Yun JY, Lee HW, Song IU, Sohn YH, Lee PH, Park JH, Oh HG, Park KW, Kwon DY (2016) Weight Change Is a Characteristic Non-Motor Symptom in Drug-Naive Parkinson's Disease Patients with Non-Tremor Dominant Subtype: A Nation-Wide Observational Study. *Plos One* **11**, 9.

[621] Papadopoulos A, Iakovakis D, Klingelhoefer L, Bostantjopoulou S, Chaudhuri KR, Kyritsis K, Hadjidimitriou S, Charisis V, Hadjileontiadis LJ, Delopoulos A (2020) Unobtrusive detection of Parkinson's disease from multi-modal and in-the-wild sensor data using deep learning techniques. *Scientific Reports* **10**, 13.

[622] Parashos SA, Luo S, Biglan KM, Bodis-Wollner I, He B, Liang GS, Ross GW, Tilley BC, Shulman LM (2014) Measuring disease progression in early Parkinson disease: the National Institutes of Health Exploratory Trials in Parkinson Disease (NET-PD) experience. *JAMA Neurol* **71**, 710-716.

[623] Prashanth R, Roy SD, Mandal PK, Ghosh S (2016) High-Accuracy Detection of Early Parkinson's Disease through Multimodal Features and Machine Learning. *International Journal of Medical Informatics* **90**, 13-21.

[624] Regnault A, Boroojerdi B, Meunier J, Bani M, Morel T, Cano S (2019) Does the MDS-UPDRS provide the precision to assess progression in early Parkinson's disease? Learnings from the Parkinson's progression marker initiative cohort. *Journal of Neurology* **266**, 1927-1936.

[625] Riesenberg R, Werth J, Zhang Y, Duvvuri S, Gray D (2020) PF-06649751 efficacy and safety in early Parkinson's disease: a randomized, placebo-controlled trial. *Therapeutic Advances in Neurological Disorders* **13**, 11.

[626] Rovini E, Moschetti A, Fiorini L, Esposito D, Maremmani C, Cavallo F, Ieee (2019) in *41st Annual International Conference of the IEEE Engineering in Medicine and Biology Society (EMBC)* Ieee, Berlin, GERMANY, pp. 4318-4321.

[627] Sadikov A, Groznik V, Mozina M, Zabkar J, Nyholm D, Memedi M, Bratko I, Georgiev D (2017) Feasibility of spirography features for objective assessment of motor function in Parkinson's disease. *Artificial Intelligence in Medicine* **81**, 54-62.

[628] Seiple W, Jennings D, Rosen RB, Borchert L, Canale L, Fagan N, Gordon MF (2016) Ophthalmologic Baseline Characteristics and 2-Year Ophthalmologic Safety Profile of Pramipexole IR Compared with Ropinirole IR in Patients with Early Parkinson's Disease. *Parkinsons Disease* **2016**, 14.

[629] Shi WY, Chiao JC (2018) Contactless hand tremor detector based on an inductive sensor. *Analog Integrated Circuits and Signal Processing* **94**, 395-403.

[630] Shi WY, Chiao JC, Ieee (2015) in *IEEE MTT-S International Microwave Workshop on RF and Wireless Technologies for Biomedical and Healthcare Applications (IMWS-BIO)* Ieee, Taipei, TAIWAN, pp. 195-196.

[631] Tripathi M, Tang CC, Feigin A, De Lucia I, Nazem A, Dhawan V, Eidelberg D (2016) Automated Differential Diagnosis of Early Parkinsonism Using Metabolic Brain Networks: A Validation Study. *Journal of Nuclear Medicine* **57**, 60-66.

[632] Wang QH, Zeng W, Dai XK Gait classification for early detection and severity rating of Parkinson's disease based on hybrid signal processing and machine learning methods. *Cognitive Neurodynamics*, 24.

[633] Williamson JR, Telfer B, Mullany R, Friedl KE (2021) Detecting Parkinson's Disease from Wrist-Worn Accelerometry in the UK Biobank. *Sensors* **21**, 18.

[634] Wu WS, Lin WY, Lee MY, Ieee (2014) in *IEEE International Conference on Systems, Man, and Cybernetics (SMC)* Ieee, San Diego, CA, pp. 1181-1185.

[635] Yang X, Ye Q, Cai G, Wang Y, Cai G (2022) PD-ResNet for Classification of Parkinson's Disease From Gait. *IEEE J Transl Eng Health Med* **10**, 2200111.
[636] Yang Z, Xie Y, Dou K, Yang L, Xie A (2023) Associations of striatal dopamine transporter binding with motor and non-motor symptoms in early Parkinson's disease. *Clin Transl Sci*.
[637] Yu CY, Wu RM (2014) Application of the University Of Pennsylvania Smell Identification Test (traditional Chinese version) for detecting olfactory deficits in early Parkinson's disease in a Taiwanese cohort. *J Parkinsons Dis* **4**, 175-180.
[638] Zou H, Aggarwal V, Stebbins GT, Müller M, Cedarbaum JM, Pedata A, Stephenson D, Simuni T, Luo S (2022) Application of longitudinal item response theory models to modeling Parkinson's disease progression. *CPT Pharmacometrics Syst Pharmacol* **11**, 1382-1392.
[639] Antonini A, Bernardi L, Calandrella D, Mancini F, Plebani M (2010) Rotigotine transdermal patch in the management of Parkinson's disease (PD) and its night-time use for PD-related sleep disorders. *Functional Neurology* **25**, 21-25.
[640] Baker WL, Silver D, White CM, Kluger J, Aberle J, Patel AA, Coleman CI (2009) Dopamine agonists in the treatment of early Parkinson's disease: A meta-analysis. *Parkinsonism & Related Disorders* **15**, 287-294.
[641] Beal MF, Oakes D, Shoulson I, Henchcliffe C, Galpern WR, Haas R, Juncos JL, Nutt JG, Voss TS, Ravina B, Shults CM, Helles K, Snively V, Lew MF, Griebner B, Watts A, Gao S, Pourcher E, Bond L, Kompoliti K, Agarwal P, Sia C, Jog M, Cole L, Sultana M, Kurlan R, Richard I, Deeley C, Waters CH, Figueroa A, Arkun A, Brodsky M, Ondo WG, Hunter CB, Jimenez-Shahed J, Palao A, Miyasaki JM, Julie SO, Tetrud J, Reys L, Smith K, Singer C, Blenke A, Russell DS, Cotto C, Friedman JH, Lannon M, Zhang L, Drasby E, Kumar R, Subramanian T, Ford DS, Grimes DA, Cote D, Conway J, Siderowf AD, Evatt ML, Sommerfeld B, Lieberman AN, Okun MS, Rodriguez RL, Merritt S, Swartz CL, Martin WRW, King P, Stover N, Guthrie S, Watts RL, Ahmed A, Fernandez HH, Winters A, Mari Z, Dawson TM, Dunlop B, Feigin AS, Shannon B, Nirenberg MJ, Ogg M, Ellias SA, Thomas CA, Frei K, Bodis-Wollner I, Glazman S, Mayer T, Hauser RA, Pahwa R, Langhammer A, Ranawaya R, Derwent L, Sethi KD, Farrow B, Prakash R, Litvan I, Robinson A, Sahay A, Gartner M, Hinson VK, Markind S, Pelikan M, Perlmutter JS, Hartlein J, Molho E, Evans S, Adler CH, Duffy A, Lind M, Elmer L, Davis K, Spears J, Wilson S, Leehey MA, Hermanowicz N, Niswonger S, Shill HA, Obradov S, Rajput A, Cowper M, Lessig S, Song D, Fontaine D, Zadikoff C, Williams K, Blindauer KA, Bergholte J, Propsom CS, Stacy MA, Field J, Mihaila D, Chilton M, Uc EY, Sieren J, Simon DK, Kraics L, Silver A, Boyd JT, Hamill RW, Ingvoldstad C, Young J, Thomas K, Kostyk SK, Wojcieszek J, Pfeiffer RF, Panisset M, Beland M, Reich SG, Cines M, Zappala N, Rivest J, Zweig R, Lumina LP, Hilliard CL, Grill S, Kellermann M, Tuite P, Rolandelli S, Kang UJ, Young J, Rao J, Cook MM, Severt L, Boyar K (2014) A Randomized Clinical Trial of High-Dosage Coenzyme Q10 in Early Parkinson Disease No Evidence of Benefit. *Jama Neurology* **71**, 543-552.
[642] Blindauer K, Shoulson I, Kieburtz K, McDermott M, Gardiner I, Kamp C, Marshall F, Zhang L, Shinaman MA, Fahn S, Suchowersky O, Wooten FG, Frei K, Pathak M, Luong N, Tuite P, Schacherer R, Jennings D, Stavris K, Wojcieszek J, Elmer L, Aiken L, Rajput A, Rajput A, Ewanishin M, Shirley T, Golbe L, Caputo D, Dewey R, Estes B, DeMarcaida T, Counihan T, Deeley C, Jankovic J, Hunter C, Fernandez HH, Lannon MC, Hubble J, La Fontaine AL,

Pantella C, Derwent L, Calabrese V, Roberge P, Lou JS, Andrews P, Nieves A, Sime E, Shults C, Fontaine D, Racette B, Cooper P, Welsh M, Kawai C, Waters C, Hauser R, Gauger L, Panisset M, Hall J, O'Brien C, Judd D, Dalvi A, Schwieteren D, Mahant P, Williamson K, Christine C, Hevezi J, Kang UJ, Richman J, Kompoliti K, Jaglin J, Trugman J, Rost-Ruffner E, Grimes D, Colcher A, Reichwein S, Tarsy D, Ryan P, Bertoni J, Peterson C, Atchison P, Allen C, Curran T, Bailey S, Brocht A, Hodgeman K, Josephson L, Lenio E, O'Connell C, Rothenburgh K, Rumfola L, Watts A, Weaver C, Tariot P, Raubertas R, Chase T, Goodin T, Bianchine J, Woltering F, Mendzelevski B, Parkinson Study G, Steering C, Safety Monitoring C (2003) A controlled trial of rotigotine monotherapy in early Parkinson's disease. *Archives of Neurology* **60**, 1721-1728.

[643] Dietiker C, Kim S, Zhang Y, Christine CW (2019) Characterization of Vitamin B12 Supplementation and Correlation with Clinical Outcomes in a Large Longitudinal Study of Early Parkinson's Disease. *J Mov Disord* **12**, 91-96.

[644] Doiron M, Langlois M, Dupre N, Simard M (2018) The influence of vascular risk factors on cognitive function in early Parkinson's disease. *International Journal of Geriatric Psychiatry* **33**, 288-297.

[645] Espay AJ, Foster ED, Coffey CS, Uribe L, Caspell-Garcia CJ, Weintraub D, Parkinson's Progression Markers I (2019) Lack of independent mood-enhancing effect for dopaminergic medications in early Parkinson's disease. *Journal of the Neurological Sciences* **402**, 81-85.

[646] Fiorilli G, Quinzi F, Buonsenso A, Casazza G, Manni L, Parisi A, Di Costanzo A, Calcagno G, Soligo M, di Cagno A (2021) A Single Session of Whole-Body Electromyostimulation Increases Muscle Strength, Endurance and proNGF in Early Parkinson Patients. *International Journal of Environmental Research and Public Health* **18**, 14.

[647] Fishel SC, Hotchkiss ME, Brown SA (2020) The impact of LSVT BIG therapy on postural control for individuals with Parkinson disease: A case series. *Physiotherapy Theory and Practice* **36**, 834-843.

[648] Frequin HL, Schouten J, Verschuur CVM, Suwijn SR, Boel JA, Post B, Bloem BR, van Hilten JJ, van Laar T, Tissingh G, Munts AG, Dijk JM, Deuschl G, Lang A, Dijkgraaf MGW, de Haan RJ, de Bie RMA (2023) Levodopa Response in Patients With Early Parkinson Disease: Further Observations of the LEAP Study. *Neurology* **100**, e367-e376.

[649] Giladi N, Nicholas AP, Asgharnejad M, Dohin E, Woltering F, Bauer L, Poewe W (2016) Efficacy of Rotigotine at Different Stages of Parkinson's Disease Symptom Severity and Disability: A Post Hoc Analysis According to Baseline Hoehn and Yahr Stage. *Journal of Parkinsons Disease* **6**, 741-749.

[650] Gray R, Ives N, Rick C, Patel S, Gray A, Jenkinson C, McIntosh E, Wheatley K, Williams A, Clarke CE (2014) Long-term effectiveness of dopamine agonists and monoamine oxidase B inhibitors compared with levodopa as initial treatment for Parkinson's disease (PD MED): a large, open-label, pragmatic randomised trial. *Lancet* **384**, 1196-1205.

[651] Hacker ML, Tonascia J, Turchan M, Currie A, Heusinkveld L, Konrad PE, Davis TL, Neimat JS, Phibbs FT, Hedera P, Wang L, Shi YP, Shade DM, Sternberg AL, Drye LT, Charles D (2015) Deep brain stimulation may reduce the relative risk of clinically important worsening in early stage Parkinson's disease. *Parkinsonism & Related Disorders* **21**, 1177-1183.

[652] Hanna-Pladdy B, Pahwa R, Lyons KE (2015) Paradoxical Effect of Dopamine Medication on Cognition in Parkinson's Disease: Relationship to Side of Motor Onset. *Journal of the International Neuropsychological Society* **21**, 259-270.

[653] Hatori K, Kondo T, Mizuno Y (1997) [Levodopa loading test as an early marker of Parkinson's disease]. *Nihon Rinsho* **55**, 207-212.

[654] Hattori N, Takeda A, Takeda S, Nishimura A, Kitagawa T, Mochizuki H, Nagai M, Takahashi R (2019) Long-term, open-label, phase 3 study of rasagiline in Japanese patients with early Parkinson's disease. *Journal of Neural Transmission* **126**, 299-308.

[655] Hauser RA, Abler V, Eyal E, Eliaz RE (2016) Efficacy of rasagiline in early Parkinson's disease: a meta-analysis of data from the TEMPO and ADAGIO studies. *International Journal of Neuroscience* **126**, 942-946.

[656] Hauser RA, Schapira AHV, Barone P, Mizuno Y, Rascol O, Busse M, Debieuvre C, Fraessdorf M, Poewe W, Pramipexole ERSG (2014) Long-term safety and sustained efficacy of extended-release pramipexole in early and advanced Parkinson's disease. *European Journal of Neurology* **21**, 736-743.

[657] Hauser RA, Silver D, Choudhry A, Eyal E, Isaacson S, Investigators AS (2014) Randomized, Controlled Trial of Rasagiline as an Add-on to Dopamine Agonists in Parkinson's Disease. *Movement Disorders* **29**, 1028-1034.

[658] Hauser RA, Slawek J, Barone P, Dohin E, Surmann E, Asgharnejad M, Bauer L (2016) Evaluation of rotigotine transdermal patch for the treatment of apathy and motor symptoms in Parkinson's disease. *Bmc Neurology* **16**, 12.

[659] Hubble JP (2000) Pre-clinical studies of pramipexole: clinical relevance. *European Journal of Neurology* **7**, 15-20.

[660] Hubble JP, Koller WC, Cutler NR, Sramek JJ, Friedman J, Goetz C, Ranhosky A, Korts D, Elvin A (1995) PRAMIPEXOLE IN PATIENTS WITH EARLY PARKINSONS-DISEASE. *Clinical Neuropharmacology* **18**, 338-347.

[661] Jankovic J, Berkovich E, Eyal E, Tolosa E (2014) Symptomatic efficacy of rasagiline monotherapy in early Parkinson's disease: Post-hoc analyses from the ADAGIO trial. *Parkinsonism & Related Disorders* **20**, 640-643.

[662] Kandadai RM, Jabeen SA, Kanikannan MA, Borgohain R (2014) Safinamide for the treatment of Parkinson's disease. *Expert Review of Clinical Pharmacology* **7**, 747-759.

[663] Khlebtovsky A, Steiner I, Treves T, Djaldetti R (2019) Effect of Repeated Intravenous Amantadine Infusions in Patients with Parkinson's Disease: An Open-Label Pilot Study. *Clin Transl Sci* **12**, 586-590.

[664] Kim R, Jun JS (2020) Impact of Overweight and Obesity on Functional and Clinical Outcomes of Early Parkinson's Disease. *J Am Med Dir Assoc* **21**, 697-700.

[665] Korczyn AD, Brunt ER, Larsen JP, Nagy Z, Poewe WH, Ruggieri S, Study G (1999) A 3-year randomized trial of ropinirole and bromocriptine in early Parkinson's disease. *Neurology* **53**, 364-370.

[666] Lambert D, Waters CH (2000) Comparative tolerability of the newer generation antiparkinsonian agents. *Drugs & Aging* **16**, 55-65.

[667] Lew MF (2013) Rasagiline treatment effects on parkinsonian tremor. *International Journal of Neuroscience* **123**, 859-865.

[668] Lieberman A, Minagar A, Pinter MM (2001) The efficacy of pramipexole in the treatment of Parkinson's disease. *Reviews in Contemporary Pharmacotherapy* **12**, 59-86.

[669] MacDonald AA, Monchi O, Seergobin KN, Ganjavi H, Tamjeedi R, MacDonald PA (2013) Parkinson's disease duration determines effect of dopaminergic therapy on ventral striatum function. *Movement Disorders* **28**, 153-160.

[670] Mao ZL, Modi NB (2016) Dose-Response Analysis of the Effect of Carbidopa-Levodopa Extended-Release Capsules (IPX066) in Levodopa-Naive Patients With Parkinson Disease. *Journal of Clinical Pharmacology* **56**, 974-982.

[671] Marconi S, Zwingers T (2014) Comparative efficacy of selegiline versus rasagiline in the treatment of early Parkinson's disease. *European Review for Medical and Pharmacological Sciences* **18**, 1879-1882.

[672] Marsala SZ, Vitaliani R, Volpe D, Capozzoli F, Baroni L, Belgrado E, Borsato C, Gioulis M, Marchini C, Antonini A (2013) Rapid onset of efficacy of rasagiline in early Parkinson's disease. *Neurological Sciences* **34**, 2007-2013.

[673] Mayer JS, Neimat J, Folley BS, Bourne SK, Konrad PE, Charles D, Park S (2016) Deep brain stimulation of the subthalamic nucleus alters frontal activity during spatial working memory maintenance of patients with Parkinson's disease. *Neurocase* **22**, 369-378.

[674] Mestre TA, Shah P, Marras C, Tomlinson G, Lang AE (2014) Another face of placebo: The lessebo effect in Parkinson disease Meta-analyses. *Neurology* **82**, 1402-1409.

[675] Nackaerts E, Michely J, Heremans E, Swinnen SP, Smits-Engelsman BCM, Vandenberghe W, Grefkes C, Nieuwboer A (2018) Training for Micrographia Alters Neural Connectivity in Parkinson's Disease. *Frontiers in Neuroscience* **12**, 11.

[676] Oh YS, Kim JS, Yoo SW, Hwang EJ, Lyoo CH, Lee KS (2019) Striatal dopamine activity and myocardial (123)I-metaiodobenzylguanidine uptake in early Parkinson's disease. *Parkinsonism Relat Disord* **63**, 156-161.

[677] Ohara M, Hirata K, Hallett M, Matsubayashi T, Chen Q, Kina S, Shimano K, Hirakawa A, Yokota T, Hattori T (2023) Long-term levodopa ameliorates sequence effect in simple, but not complex walking in early Parkinson's disease patients. *Parkinsonism Relat Disord* **108**, 105322.

[678] Olszewska DA, Fasano A, Munhoz RP, Gomez CCR, Lang AE (2022) Initiating dopamine agonists rather than levodopa in early Parkinson's disease does not delay the need for deep brain stimulation. *European Journal of Neurology* **29**, 3742-3747.

[679] Omoto S, Saito M, Murakami H, Shiraishi T, Kitagawa T, Sato T, Takatsu H, Komatsu T, Sakai K, Umehara T, Mitsumura H, Iguchi Y (2022) The association between urinary pentosidine levels and cognition in drug-naive patients with Parkinson's disease. *Neurological Sciences* **43**, 6323-6328.

[680] Pachalska M, Goral-Polrola J, Jarosz P (2022) NEUROTHERAPY IN PARKINSON?S DISEASE: THE PATH FORWARD AFTER SARS-COV-2 INFECTION AND CONTRACTING COVID-19 , AND LONG COVID? *Acta Neuropsychologica* **20**, 275-290.

[681] Pagano G, Boess FG, Taylor KI, Ricci B, Mollenhauer B, Poewe W, Boulay A, Anzures-Cabrera J, Vogt A, Marchesi M, Post A, Nikolcheva T, Kinney GG, Zago WM, Ness DK, Svoboda H, Britschgi M, Ostrowitzki S, Simuni T, Marek K, Koller M, Sevigny J, Doody R, Fontoura P, Umbricht D, Bonni A, Investigators P, Prasinezumab Study G (2021) A Phase II

Study to Evaluate the Safety and Efficacy of Prasinezumab in Early Parkinson's Disease (PASADENA): Rationale, Design, and Baseline Data. *Frontiers in Neurology* **12**, 17.

[682] Pah ND, Motin MA, Kempster P, Kumar DK (2021) Detecting Effect of Levodopa in Parkinson's Disease Patients Using Sustained Phonemes. *Ieee Journal of Translational Engineering in Health and Medicine* **9**, 9.

[683] Pahwa R, Lyons KE, Hauser RA, Fahn S, Jankovic J, Pourcher E, Hsu A, O'Connell M, Kell S, Gupta S, Investigators A-P (2014) Randomized trial of IPX066, carbidopa/levodopa extended release, in early Parkinson's disease. *Parkinsonism & Related Disorders* **20**, 142-148.

[684] Palermo G, Giannoni S, Giuntini M, Belli E, Frosini D, Siciliano G, Ceravolo R (2021) Statins in Parkinson's Disease: Influence on Motor Progression. *Journal of Parkinsons Disease* **11**, 1651-1662.

[685] Pantzaris M, Loukaides G, Paraskevis D, Kostaki EG, Patrikios I (2021) Neuroaspis PLP10 (TM), a nutritional formula rich in omega-3 and omega-6 fatty acids with antioxidant vitamins including gamma-tocopherol in early Parkinson's disease: A randomized, double-blind, placebo-controlled trial. *Clinical Neurology and Neurosurgery* **210**, 7.

[686] Poewe W, Hauser RA, Lang A (2015) Effects of rasagiline on the progression of nonmotor scores of the MDS-UPDRS. *Mov Disord* **30**, 589-592.

[687] Poewe W, Hauser RA, Lang A, Investigators A (2015) Effects of Rasagiline on the Progression of Nonmotor Scores of the MDS-UPDRS. *Movement Disorders* **30**, 589-592.

[688] Rascol O, Fitzer-Attas C, Hauser R, Jankovic J, Long A, Langston JW, Melamed E, Poewe W, Stocchi F, Tolosa E, Eyal E, Weiss YM, Olanow CW (2011) A double-blind, delayed-start trial of rasagiline in Parkinson's disease (the ADAGIO study): prespecified and post-hoc analyses of the need for additional therapies, changes in UPDRS scores, and non-motor outcomes. *Lancet Neurology* **10**, 415-423.

[689] Schwarzschild MA, Ascherio A, Beal MF, Cudkowicz ME, Curhan GC, Hare JM, Hooper DC, Kieburtz KD, Macklin EA, Oakes D, Rudolph A, Shoulson I, Tennis MK, Espay AJ, Gartner M, Hung A, Bwala G, Lenehan R, Encarnacion E, Ainslie M, Castillo R, Togasaki D, Barles G, Friedman JH, Niles L, Carter JH, Murray M, Goetz CG, Jaglin J, Ahmed A, Russell DS, Cotto C, Goudreau JL, Russell D, Parashos SA, Ede P, Saint-Hilaire MH, Thomas CA, James R, Stacy MA, Johnson J, Gauger L, de Marcaida JA, Thurlow S, Isaacson SH, Carvajal L, Rao J, Cook M, Hope-Porche C, McClurg L, Grasso DL, Logan R, Orme C, Ross T, Brocht AFD, Constantinescu R, Sharma S, Venuto C, Weber J, Eaton K (2014) Inosine to Increase Serum and Cerebrospinal Fluid Urate in Parkinson Disease A Randomized Clinical Trial. *Jama Neurology* **71**, 141-150.

[690] Sklerov M, Browner N, Dayan E, Rubinow D, Frohlich F (2022) Autonomic and Depression Symptoms in Parkinson's Disease: Clinical Evidence for Overlapping Physiology. *Journal of Parkinsons Disease* **12**, 1059-1067.

[691] Smith KM, Eyal E, Weintraub D (2015) Combined rasagiline and antidepressant use in Parkinson disease in the ADAGIO study: effects on nonmotor symptoms and tolerability. *JAMA Neurol* **72**, 88-95.

[692] Smith KM, Eyal E, Weintraub D, Investigators A (2015) Combined Rasagiline and Antidepressant Use in Parkinson Disease in the ADAGIO Study Effects on Nonmotor Symptoms and Tolerability. *Jama Neurology* **72**, 88-95.

[693] So HY, Kim SR, Kim S, Park YS, Jo S, Park KW, Choi N, Lee SH, Hwang YS, Kim MS, Chung SJ (2023) Effect of Home-Based Self-Management Intervention for Community-Dwelling Patients with Early Parkinson's Disease: A Feasibility Study. *J Community Health Nurs* **40**, 133-146.

[694] Stocchi F, Investigators A (2014) Benefits of treatment with rasagiline for fatigue symptoms in patients with early Parkinson's disease. *European Journal of Neurology* **21**, 357-360.

[695] Thomas A, Bonanni L, Di Iorio A, Varanese S, Anzellotti F, D'Andreagiovanni A, Stocchi F, Onofrj M (2006) End-of-dose deterioration in non ergolinic dopamine agonist monotherapy of Parkinson's disease. *Journal of Neurology* **253**, 1633-1639.

[696] Timmermann L, Asgharnejad M, Boroojerdi B, Dohin E, Woltering F, Elmer LW (2015) Impact of 6-month earlier versus postponed initiation of rotigotine on long-term outcome: post hoc analysis of patients with early Parkinson's disease with mild symptom severity. *Expert Opinion on Pharmacotherapy* **16**, 1423-1433.

[697] Todt I, Al-Fatly B, Granert O, Kuhn AA, Krack P, Rau J, Timmermann L, Schnitzler A, Paschen S, Helmers AK, Hartmann A, Bardinet E, Schuepbach M, Barbe MT, Dembek TA, Fraix V, Kubler D, Brefel-Courbon C, Gharabaghi A, Wojtecki L, Pinsker MO, Thobois S, Damier P, Witjas T, Houeto JL, Schade-Brittinger C, Vidailhet M, Horn A, Deuschl G (2022) The Contribution of Subthalamic Nucleus Deep Brain Stimulation to the Improvement in Motor Functions and Quality of Life. *Movement Disorders* **37**, 291-301.

[698] Tramontana MG, Molinari AL, Konrad PE, Davis TL, Wylie SA, Neimat JS, May AT, Phibbs FT, Hedera P, Gill CE, Salomon RM, Wang L, Song YN, Charles D (2015) Neuropsychological Effects of Deep Brain Stimulation in Subjects with Early Stage Parkinson's Disease in a Randomized Clinical Trial. *Journal of Parkinsons Disease* **5**, 151-163.

[699] Verschuur CVM, Suwijn SR, Boel JA, Post B, Bloem BR, van Hilten JJ, van Laar T, Tissingh G, Munts AG, Deuschl G, Lang AE, Dijkgraaf MGW, de Haan RJ, de Bie RMA, Grp LS (2019) Randomized Delayed-Start Trial of Levodopa in Parkinson's Disease. *New England Journal of Medicine* **380**, 315-324.

[700] Wang CC, Wu TL, Lin FJ, Tai CH, Lin CH, Wu RM (2022) Amantadine treatment and delayed onset of levodopa-induced dyskinesia in patients with early Parkinson's disease. *European Journal of Neurology* **29**, 1044-1055.

[701] Wu CX, Guo HJ, Xu YS, Li LP, Li XY, Tang CZ, Chen DF, Zhu ML (2022) The Comparative Efficacy of Non-ergot Dopamine Agonist and Potential Risk Factors for Motor Complications and Side Effects From NEDA Use in Early Parkinson's Disease: Evidence From Clinical Trials. *Frontiers in Aging Neuroscience* **14**, 10.

[702] Yoritaka A, Kawajiri S, Yamamoto Y, Nakahara T, Ando M, Hashimoto K, Nagase M, Saito Y, Hattori N (2015) Randomized, double-blind, placebo-controlled pilot trial of reduced coenzyme Q(10) for Parkinson's disease. *Parkinsonism & Related Disorders* **21**, 911-916.

[703] Zambito Marsala S, Vitaliani R, Volpe D, Capozzoli F, Baroni L, Belgrado E, Borsato C, Gioulis M, Marchini C, Antonini A (2013) Rapid onset of efficacy of rasagiline in early Parkinson's disease. *Neurol Sci* **34**, 2007-2013.

[704] Zesiewicz TA, Chriscoe S, Jimenez T, Upward J, VanMeter S (2017) A fixed-dose, dose-response study of ropinirole prolonged release in early stage Parkinson's disease. *Neurodegenerative Disease Management* **7**, 49-59.

[705] Zhang ZX, Wang J, Chen SD, Liu CF, Zhang BR, Peng R, Sun SG, Sun XR, Zhao G, Qu QM, Li YS, Zhu SQ, Pan XP, Shao M, Wang YP (2018) Efficacy and safety of rasagiline in Chinese patients with early Parkinson's disease: a randomized, double-blind, parallel, placebo-controlled, fixed-dose study. *Translational Neurodegeneration* **7**, 9.

[706] (2022) Boxing may help with Parkinson's symptoms. *Indian Practitioner* **75**, 48-49.

[707] Adelon J, Dufour C, Foulon S, Planchon JM, Meyronnet D, Bourdeaut F, Palenzuela G, Fouyssac F, Raimbault S, De Carli E, Klein S, Pagnier A, Bertozzi AI, Rome A, David A, Chabaud S, Faure-Conter C (2021) What does a non-response to induction chemotherapy imply in high-risk medulloblastomas? *Journal of Neuro-Oncology* **153**, 425-440.

[708] Betancourt E, Wachtel J, Michaelos M, Haggerty M, Conforti J, Kritzer MF (2017) THE IMPACT OF BIOLOGICAL SEX AND SEX HORMONES ON COGNITION IN A RAT MODEL OF EARLY, PRE-MOTOR PARKINSON'S DISEASE. *Neuroscience* **345**, 297-314.

[709] Brehm N, Bez F, Carlsson T, Kern B, Gispert S, Auburger G, Cenci MA (2015) A Genetic Mouse Model of Parkinson's Disease Shows Involuntary Movements and Increased Postsynaptic Sensitivity to Apomorphine. *Molecular Neurobiology* **52**, 1152-1164.

[710] Butkovich LM, Houser MC, Chalermpalanupap T, Porter-Stransky KA, Iannitelli AF, Boles JS, Lloyd GM, Coomes AS, Eidson LN, De Sousa Rodrigues ME, Oliver DL, Kelly SD, Chang J, Bengoa-Vergniory N, Wade-Martins R, Giasson BI, Joers V, Weinshenker D, Tansey MG (2020) Transgenic Mice Expressing Human α-Synuclein in Noradrenergic Neurons Develop Locus Ceruleus Pathology and Nonmotor Features of Parkinson's Disease. *J Neurosci* **40**, 7559-7576.

[711] Casanova Y, Negro S, Slowing K, Garcia-Garcia L, Fernandez-Carballido A, Rahmani M, Barcia E (2022) Micro- and Nano-Systems Developed for Tolcapone in Parkinson's Disease. *Pharmaceutics* **14**, 15.

[712] Cisbani G, Drouin-Ouellet J, Gibrat C, Saint-Pierre M, Lagace M, Badrinarayanan S, Lavallee-Bourget MH, Charest J, Chabrat A, Boivin L, Lebel M, Bousquet M, Levesque M, Cicchetti F (2015) Cystamine/cysteamine rescues the dopaminergic system and shows neurorestorative properties in an animal model of Parkinson's disease. *Neurobiology of Disease* **82**, 430-444.

[713] Conner MR, Jang DY, Anderson BJ, Kritzer MF (2020) Biological Sex and Sex Hormone Impacts on Deficits in Episodic-Like Memory in a Rat Model of Early, Pre-motor Stages of Parkinson's Disease. *Frontiers in Neurology* **11**, 22.

[714] de Campos PS, Hasegawa K, Kumei Y, Zeredo JL (2015) Cineradiographic analysis of respiratory movements in a mouse model for early Parkinson's disease. *Respiratory Physiology & Neurobiology* **218**, 40-45.

[715] Dranka BP, Gifford A, Ghosh A, Zielonka J, Joseph J, Kanthasamy AG, Kalyanaraman B (2013) Diapocynin prevents early Parkinson's disease symptoms in the leucine-rich repeat kinase 2 (LRRK2(R1441G)) transgenic mouse. *Neuroscience Letters* **549**, 57-62.

[716] Dranka BP, Gifford A, McAllister D, Zielonka J, Joseph J, O'Hara CL, Stucky CL, Kanthasamy AG, Kalyanaraman B (2014) A novel mitochondrially-targeted apocynin derivative prevents hyposmia and loss of motor function in the leucine-rich repeat kinase 2 (LRRK2(R1441G)) transgenic mouse model of Parkinson's disease. *Neuroscience Letters* **583**, 159-164.

[717] Du L, Xu L, Liang T, Wing YK, Ke Y, Yung WH (2021) Progressive Pontine-Medullary Dysfunction Leads to REM Sleep Behavior Disorder Symptoms in a Chronic Model of Parkinson's Disease. *Nat Sci Sleep* **13**, 1723-1736.

[718] Du LD, Xu LH, Liang T, Wing YK, Ke Y, Yung H (2021) Progressive Pontine-Medullary Dysfunction Leads to REM Sleep Behavior Disorder Symptoms in a Chronic Model of Parkinson's Disease. *Nature and Science of Sleep* **13**, 1723-1736.

[719] Ekmark-Lewen S, Lindstrom V, Gumucio A, Ihse E, Behere A, Kahle PJ, Nordstrom E, Eriksson M, Erlandsson A, Bergstrom J, Ingelsson M (2018) Early fine motor impairment and behavioral dysfunction in (Thy-1)-h A30P alpha-synuclein mice. *Brain and Behavior* **8**, 14.

[720] Ginns EI, Mak SKK, Ko N, Karlgren J, Akbarian S, Chou VP, Guo Y, Lim A, Samuelsson S, LaMarca ML, Vazquez-DeRose J, Manning-Bog AB (2014) Neuroinflammation and alpha-synuclein accumulation in response to glucocerebrosidase deficiency are accompanied by synaptic dysfunction. *Molecular Genetics and Metabolism* **111**, 152-162.

[721] Gries M, Christmann A, Schulte S, Weyland M, Rommel S, Martin M, Baller M, Roth R, Schmitteckert S, Unger M, Liu Y, Sommer F, Muhlhaus T, Schroda M, Timmermans JP, Pintelon I, Rappold GA, Britschgi M, Lashuel H, Menger MD, Laschke MW, Niesler B, Schafer KH (2021) Parkinson mice show functional and molecular changes in the gut long before motoric disease onset. *Molecular Neurodegeneration* **16**, 23.

[722] Gu PS, Moon M, Choi JG, Oh MS (2017) Mulberry fruit ameliorates Parkinson's-disease-related pathology by reducing alpha-synuclein and ubiquitin levels in a 1-methyl-4-phenyl-1,2,3,6-tetrahydropyridine/probenecid model. *Journal of Nutritional Biochemistry* **39**, 15-21.

[723] Hsieh TH, Huang YZ, Rotenberg A, Pascual-Leone A, Chiang YH, Wang JY, Chen JJJ (2015) Functional Dopaminergic Neurons in Substantia Nigra are Required for Transcranial Magnetic Stimulation-Induced Motor Plasticity. *Cerebral Cortex* **25**, 1806-1814.

[724] Hussein A, Tielemans A, Baxter MG, Benson DL, Huntley GW (2022) Cognitive deficits and altered cholinergic innervation in young adult male mice carrying a Parkinson's disease Lrrk2(G2019S)knockin mutation. *Experimental Neurology* **355**, 13.

[725] Jalali-Nadoushan M, Roghani M (2013) Alpha-lipoic acid protects against 6-hydroxydopamine-induced neurotoxicity in a rat model of hemi-parkinsonism. *Brain Research* **1505**, 68-74.

[726] Jenner P (2014) An overview of adenosine A2A receptor antagonists in Parkinson's disease. *Int Rev Neurobiol* **119**, 71-86.

[727] Jiao FJ, Wang QZ, Zhang P, Bu LL, Yan JG, Tian B (2017) Expression signatures of long non-coding RNA in the substantia nigra of pre-symptomatic mouse model of Parkinson's disease. *Behavioural Brain Research* **331**, 123-130.

[728] Johnson ME, Zhou XF, Bobrovskaya L (2019) The effects of rotenone on TH, BDNF and BDNF-related proteins in the brain and periphery: Relevance to early Parkinson's disease. *Journal of Chemical Neuroanatomy* **97**, 23-32.

[729] Kojovic M, Kassavetis P, Bologna M, Parees I, Rubio-Agusti I, Beraredelli A, Edwards MJ, Rothwell JC, Bhatia KP (2015) Transcranial magnetic stimulation follow-up study in early Parkinson's disease: A decline in compensation with disease progression? *Movement Disorders* **30**, 1098-1106.

[730] Kryukova EV, Shelukhina IV, Kolacheva AA, Alieva AK, Shadrina MI, Slominsky PA, Kasheverov IE, Utkin YN, Ugrumov MV, Tsetlin VI (2017) Possible Involvement of Neuronal Nicotinic Acetylcholine Receptors in Compensatory Brain Mechanisms at Early Stages of Parkinson's Disease. *Biochemistry Moscow-Supplement Series B-Biomedical Chemistry* **11**, 363-370.

[731] Kucinski A, Paolone G, Bradshaw M, Albin RL, Sarter M (2013) Modeling Fall Propensity in Parkinson's Disease: Deficits in the Attentional Control of Complex Movements in Rats with Cortical-Cholinergic and Striatal-Dopaminergic Deafferentation. *Journal of Neuroscience* **33**, 16522-16539.

[732] Kuo YM, Nwankwo EI, Nussbaum RL, Rogers J, Maccecchini ML (2019) Translational inhibition of α-synuclein by Posiphen normalizes distal colon motility in transgenic Parkinson mice. *Am J Neurodegener Dis* **8**, 1-15.

[733] Kurnik M, Thor P (2015) The non-motor complications in Parkinson's disease- what can we learn from animal models? *Folia Med Cracov* **55**, 69-84.

[734] Kurtenbach S, Wewering S, Hatt H, Neuhaus EM, Lubbert H (2013) Olfaction in Three Genetic and Two MPTP-Induced Parkinson's Disease Mouse Models. *Plos One* **8**, 9.

[735] Kuter K, Kratochwil M, Berghauzen-Maciejewska K, Glowacka U, Sugawa MD, Ossowska K, Dencher NA (2016) Adaptation within mitochondrial oxidative phosphorylation supercomplexes and membrane viscosity during degeneration of dopaminergic neurons in an animal model of early Parkinson's disease. *Biochimica Et Biophysica Acta-Molecular Basis of Disease* **1862**, 741-753.

[736] Kuter K, Kratochwil M, Marx SH, Hartwig S, Lehr S, Sugawa MD, Dencher NA (2016) Native DIGE proteomic analysis of mitochondria from substantia nigra and striatum during neuronal degeneration and its compensation in an animal model of early Parkinson's disease. *Archives of Physiology and Biochemistry* **122**, 238-256.

[737] Kuter K, Olech Ł, Głowacka U (2018) Prolonged Dysfunction of Astrocytes and Activation of Microglia Accelerate Degeneration of Dopaminergic Neurons in the Rat Substantia Nigra and Block Compensation of Early Motor Dysfunction Induced by 6-OHDA. *Mol Neurobiol* **55**, 3049-3066.

[738] Lin XM, Shi M, Masilamoni JG, Dator R, Movius J, Aro P, Smith Y, Zhang J (2015) Proteomic profiling in MPTP monkey model for early Parkinson disease biomarker discovery. *Biochimica Et Biophysica Acta-Proteins and Proteomics* **1854**, 779-787.

[739] Mallet D, Dufourd T, Decourt M, Carcenac C, Bossu P, Verlin L, Fernagut PO, Benoit-Marand M, Spalletta G, Barbier EL, Carnicella S, Sgambato V, Fauvelle F, Boulet S (2022) A metabolic biomarker predicts Parkinson's disease at the early stages in patients and animal models. *Journal of Clinical Investigation* **132**, 17.

[740] Marsova M, Poluektova E, Odorskaya M, Ambaryan A, Revishchin A, Pavlova G, Danilenko V (2020) Protective effects of Lactobacillus fermentum U-21 against paraquat-induced oxidative stress in Caenorhabditis elegans and mouse models. *World Journal of Microbiology & Biotechnology* **36**, 10.

[741] Marsova M, Poluektova E, Odorskaya M, Ambaryan A, Revishchin A, Pavlova G, Danilenko V (2020) Protective effects of Lactobacillus fermentum U-21 against paraquat-induced oxidative stress in Caenorhabditis elegans and mouse models. *World Journal of Microbiology & Biotechnology* **36**, 104.

[742] Mourre C, Manrique C, Camon J, Aidi-Knani S, Deltheil T, Turle-Lorenzo N, Guiraudie-Capraz G, Amalric M (2017) Changes in SK channel expression in the basal ganglia after partial nigrostriatal dopamine lesions in rats: Functional consequences. *Neuropharmacology* **113**, 519-532.
[743] Natale G, Pignataro A, Marino G, Campanelli F, Calabrese V, Cardinale A, Pelucchi S, Marcello E, Gardoni F, Viscomi MT, Picconi B, Ammassari-Teule M, Calabresi P, Ghiglieri V (2021) Transcranial Magnetic Stimulation Exerts "Rejuvenation" Effects on Corticostriatal Synapses after Partial Dopamine Depletion. *Movement Disorders* **36**, 2254-2263.
[744] Niu YY, Guo XY, Chen YC, Wang CE, Gao JQ, Yang WL, Kang Y, Si W, Wang H, Yang SH, Li SH, Ji WZ, Li XJ (2015) Early Parkinson's disease symptoms in alpha-synuclein transgenic monkeys. *Human Molecular Genetics* **24**, 2308-2317.
[745] Nuber S, Tadros D, Fields J, Overk CR, Ettle B, Kosberg K, Mante M, Rockenstein E, Trejo M, Masliah E (2014) Environmental neurotoxic challenge of conditional alpha-synuclein transgenic mice predicts a dopaminergic olfactory-striatal interplay in early PD. *Acta Neuropathologica* **127**, 477-494.
[746] Okano M, Takahata K, Sugimoto J, Muraoka S (2019) Selegiline Recovers Synaptic Plasticity in the Medial Prefrontal Cortex and Improves Corresponding Depression-Like Behavior in a Mouse Model of Parkinson's Disease. *Frontiers in Behavioral Neuroscience* **13**, 15.
[747] Phillips KA, Ross CN, Spross J, Cheng CJ, Izquierdo A, Biju KC, Chen C, Li SL, Tardif SD (2017) Behavioral phenotypes associated with MPTP induction of partial lesions in common marmosets (Callithrix jacchus). *Behavioural Brain Research* **325**, 51-62.
[748] Poirier AA, Cote M, Bourque M, Morissette M, Di Paolo T, Soulet D (2016) Neuroprotective and immunomodulatory effects of raloxifene in the myenteric plexus of a mouse model of Parkinson's disease. *Neurobiology of Aging* **48**, 61-71.
[749] Renaud J, Bassareo V, Beaulieu J, Pinna A, Schlich M, Lavoie C, Murtas D, Simola N, Martinoli MG (2018) Dopaminergic neurodegeneration in a rat model of long-term hyperglycemia: preferential degeneration of the nigrostriatal motor pathway. *Neurobiology of Aging* **69**, 117-128.
[750] Revishchin A, Moiseenko L, Kust N, Bazhenova N, Teslia P, Panteleev D, Kovalzon V, Pavlova G (2016) Effects of striatal transplantation of cells transfected with GDNF gene without pre- and pro-regions in mouse model of Parkinson's disease. *Bmc Neuroscience* **17**, 15.
[751] Rowland SL, Riggs JM, Gilfillan S, Bugatti M, Vermi W, Kolbeck R, Unanue ER, Sanjuan MA, Colonna M (2014) Early, transient depletion of plasmacytoid dendritic cells ameliorates autoimmunity in a lupus model. *Journal of Experimental Medicine* **211**, 1977-1991.
[752] Sedaghat R, Roghani M, Khalili M (2014) Neuroprotective Effect of Thymoquinone, the Nigella Sativa Bioactive Compound, in 6-Hydroxydopamine-Induced Hemi-Parkinsonian Rat Model. *Iranian Journal of Pharmaceutical Research* **13**, 227-234.
[753] Shi ZD, Lee K, Yang DP, Amin S, Verma N, Li QV, Zhu ZR, Soh CL, Kumar R, Evans T, Chen SB, Huangfu DW (2017) Genome Editing in hPSCs Reveals GATA6 Haploinsufficiency and a Genetic Interaction with GATA4 in Human Pancreatic Development. *Cell Stem Cell* **20**, 675-+.
[754] Stanojlovic M, Pallais JP, Kotz CM (2021) Inhibition of Orexin/Hypocretin Neurons Ameliorates Elevated Physical Activity and Energy Expenditure in the A53T Mouse Model of Parkinson's Disease. *International Journal of Molecular Sciences* **22**, 22.

[755] Threlfell S, Mohammadi AS, Ryan BJ, Connor-Robson N, Platt NJ, Anand R, Serres F, Sharp T, Bengoa-Vergniory N, Wade-Martins R, Ewing A, Cragg SJ, Brimblecombe KR (2021) Striatal Dopamine Transporter Function Is Facilitated by Converging Biology of alpha-Synuclein and Cholesterol. *Frontiers in Cellular Neuroscience* **15**, 12.

[756] Tremblay M, Silveira MM, Kaur S, Hosking JG, Adams WK, Baunez C, Winstanley CA (2017) Chronic D(2/3) agonist ropinirole treatment increases preference for uncertainty in rats regardless of baseline choice patterns. *Eur J Neurosci* **45**, 159-166.

[757] Wang Q, Liu Z, Wang Y, Li J, Lu G, Jing Z, Liu Y, Guo Y (2018) [Effects of Xiusanzhen treatment on ultrastructure of olfactory bulb and GFAP expression in mice with Parkinson's disease]. *Zhongguo Zhen Jiu* **38**, 1093-1097.

[758] Xia Y, Ye SZ, Shi J, Huang HJ (2018) Relationship Between the Anxious Symptoms and the Neurotransmitter in Parkinson's Mice with Different Dosages of MPTP. *Brazilian Archives of Biology and Technology* **61**, 9.

[759] Yan YP, Ren SC, Duan YC, Lu CY, Niu YY, Wang ZB, Inglis B, Ji WZ, Zheng Y, Si W (2021) Gut microbiota and metabolites of alpha-synuclein transgenic monkey models with early stage of Parkinson's disease. *Npj Biofilms and Microbiomes* **7**, 9.

[760] Zare K, Eidi A, Roghani M, Rohani AH (2015) The neuroprotective potential of sinapic acid in the 6-hydroxydopamine-induced hemi-parkinsonian rat. *Metabolic Brain Disease* **30**, 205-213.

[761] Zhang SZ, Wang SJ, Shi XZ, Feng XZ (2020) Polydatin alleviates parkinsonism in MPTP-model mice by enhancing glycolysis in dopaminergic neurons. *Neurochemistry International* **139**, 11.

[762] Zhou JH, Li JC, Papaneri AB, Cui GH (2023) AJ76 and UH232 as potential agents for diagnosing early-stage Parkinson's disease. *Neuropharmacology* **226**, 10.

[763] Zhou JH, Li JC, Papaneri AB, Kobzar NP, Cui GH (2021) Dopamine Neuron Challenge Test for early detection of Parkinson's disease. *Npj Parkinsons Disease* **7**, 8.

[764] Erro R, Picillo M, Amboni M, Moccia M, Vitale C, Longo K, Pellecchia MT, Santangelo G, Martinez-Martin P, Chaudhuri KR, Barone P (2015) Nonmotor predictors for levodopa requirement in de novo patients with Parkinson's disease. *Mov Disord* **30**, 373-378.
